# Plant-Mediated Female Transcriptomic Changes Post-Mating in a Tephritid Fruit Fly, *Bactrocera tryoni*

**DOI:** 10.1093/gbe/evx257

**Published:** 2017-12-06

**Authors:** Nagalingam Kumaran, Chloé A van der Burg, Yujia Qin, Stephen L Cameron, Anthony R Clarke, Peter J Prentis

**Affiliations:** 1 School of Earth, Environmental, and Biological Sciences, Queensland University of Technology (QUT), Brisbane, Queensland, Australia; 2 School of Biomedical Sciences, Queensland University of Technology (QUT), Brisbane, Queensland, Australia; 3 Institute of Health and Biomedical Innovation, Queensland University of Technology (QUT), Brisbane, Queensland, Australia; 4 Department of Entomology, College of Plant Protection, China Agricultural University, Beijing, People’s Republic of China; 5 Department of Entomology, Purdue University, West Lafayette, IN47907, USA; 6 Institute of Future Environments, Queensland University of Technology (QUT), Brisbane, Queensland, Australia

**Keywords:** polyandry, sterile insect technique, methyl eugenol, zingerone, Tephritidae, indirect genetic effects

## Abstract

Female post-mating behaviors are regulated by complex factors involving males, females, and the environment. In insects, plant secondary compounds that males actively forage for, may indirectly modify female behaviors by altering male behavior and physiology. In the tephritid fruit fly, *Bactrocera tryoni*, females mated with males previously fed on plant-derived phenylpropanoids (=“lures” based on usage in tephritid literature), have longer mating refractoriness, greater fecundity, and reduced longevity than females mated with non-lure fed males. This system thus provides a model for studying transcriptional changes associated with those post-mating behaviors, as the genes regulating the phenotypic changes are likely to be expressed at a greater magnitude than in control females. We performed comparative transcriptome analyses using virgin *B. tryoni* females, females mated with control males (control-mated), and females mated with lure-fed males (lure-mated). We found 331 differentially expressed genes (DEGs) in control-mated females and 80 additional DEGs in lure-mated females. Although DEGs in control-mated females are mostly immune response genes and chorion proteins, as reported in *Drosophila* species, DEGs in lure-mated females are *titin*-like muscle proteins, histones, sperm, and testis expressed proteins which have not been previously reported. While transcripts regulating mating (e.g., *lingerer*) did not show differential expression in either of the mated female classes, the odorant binding protein *Obp56a* was down-regulated. The exclusively enriched or suppressed genes in lure-mated females, novel transcripts such as *titin* and histones, and several taxa-specific transcripts reported here can shed more light on post-mating transcriptional changes, and this can help understand factors possibly regulating female post-mating behaviors.

## Introduction

Mating can induce profound physiological changes and behavioral switches in females, including changes in oviposition patterns, mating refractoriness, and longevity (Thornhill and Alcock, 1983; Fowler and Partridge, 1989; Miyatake et al. 1999). These changes have a wide range of fitness impacts, and hence evolutionary implications (Andersson 1994; Elgar 1998; Arnqvist and Nilsson 2000; Jennions and Petrie 2000; Mays and Hill 2004): the intensity and tendency of these changes have been linked to sexual selection (Birkhead and Pizzari 2002), kin selection (Hughes et al. 2008), sexual conflict (Parker 1979; Chapman et al. 2003; Parker 2006), and speciation (Martin and Hosken 2003; Ritchie 2007). For example, reduced female remating receptivity is considered to directly benefit the male partner; whereas for females, reception of quality sperm from the first male confers indirect genetic benefits (e.g., in quality offspring) in mate selection scenarios (Arnqvist and Nilsson 2000; Fedorka and Mousseau 2002).

Female post-mating changes can be mediated by a complex of factors including the male, the female herself, and the environment (e.g., resource availability, operational sex ratio) (Thornhill and Alcock 1983; Andersson 1994; Aluja et al. 2009). In insects, plants with which they interact may also play a significant role in mediating those behaviors, additional to their “simple” role as a food source. For example, male euglossine bees utilize secondary plant compounds from orchids for their sex pheromone communication, and the plant compounds thus indirectly modify female behaviors (Landolt and Phillips 1997; Zimmermann et al. 2009; Weber et al. 2016). Similarly, in frugivorous fruit flies of the genus *Bactrocera* (Diptera: Tephritidae), plant secondary compounds such as methyl eugenol, raspberry ketone, and zingerone modify sexual calling and mediate male mating success (Shelly 2010). These compounds are known commonly as “male lures” or simply “lures” in the tephritid literature, because it is the males which predominantly respond to and feed upon these compounds, and the chemicals are used as lures in monitoring and pest management: this generic terminology is used in this paper hereafter.

Unlike the condition-dependent preferences and sexual selection recorded in many systems (Andersson 1986; Cotton et al. 2006), female preference for lure-fed males in *Bactrocera* is not due to specific conditions as lures do not provide any direct dietary benefits (Raghu et al. 2002). Further, lures are not an essential component of mating in *Bactrocera*, but they do very commonly modify mate selection (Raghu 2004; Shelly 2010; Kumaran et al. 2014a). The lure-mediated male mating success is apparent in several *Bactrocera* species (Shelly and Villalobos 1995; Shelly and Nishida 2004; Wee et al. 2007; Shelly et al. 2010, Obra and Resilva 2013; Haq et al. 2014), although not ubiquitous across all species (Raghu and Clarke 2003; Shelly 2017). Although there are well documented direct, lure-induced male behavioral changes in most species studied, female changes after mating with a lure-fed male are far less studied and the results are contradictory. Shelly (2000) and Shelly and Nishimoto (2016) found no evidence for female post-mating changes in *Bactrocera dorsalis* and *Zeugodacus curcurbitae*, respectively, after they had mated with lure-fed males. However, in *B. tryoni*, mating with a lure-fed male indirectly suppressed female receptivity for remating and induced greater life-time fecundity over a shorter period (Kumaran et al. 2013).

The mechanisms mediating the female post-mating changes in *B. tryoni* are not known; but they must be indirect through modified male pheromone compounds and/or other lure-modified male physiological traits (Hee and Tan 1998; Kumaran et al. 2014a, 2014b). Lures induce expression of numerous energy metabolic genes and pathways in males, and empirical evidence suggests that lure-fed males become physically fitter than non-lure fed males (Kumaran et al. 2014b). Additionally, several accessory gland protein (Acp) transcripts are differentially expressed in lure-fed *B. tryoni* compared with non-lure fed males (Kumaran et al. 2014b), and this could possibly mediated the female post-mating phenotypic changes observed. Generally, Acps or sperm that males transfer to females during copulation are regarded as the proximate mechanisms mediating phenotypic changes occurring in females after mating (Chen et al. 1988; Herndon and Wolfner 1995; Wolf et al. 1998; Chapman et al. 2003; Gillott 2003; Wigby et al. 2009; Sitnik et al. 2016). For instance, in knock-down studies using *D. melanogaster*, it was confirmed that female receptivity for remating is regulated by male produced sex peptide (*Acp70a*) (Chapman et al. 2003; Liu and Kubli 2003), and in tephritid fruit flies, *B. tryoni* and *Ceratitis capitata*, Acps were found to induce sexual inhibition (Radhakrishnan and Taylor 2008) and oviposition (Jang 1995; Scolari et al. 2012).

Transcriptome profiling of females post-mating offers insights to understand the mechanisms regulating female post-mating behaviors (e.g., Lawniczak and Begun 2004; McGraw et al. 2004; Bono et al. 2011). For example, McGraw et al. (2008) identified >2,500 genes and Lawniczak and Begun (2004) found 38 differentially expressed genes (DEGs) in *D. melanogaster* females post-mating. Among the DEGs were several genes of known function, including *Odorant binding protein 99a*, *immune response genes*, *yolk proteins*, and *chorion proteins*. Studying transcriptional changes exclusively in the reproductive tracts of *Drosophila mojavensis*, Bono et al. (2011) found 12 female-origin genes out of 18 DEGs including *Obp93A* and immune response transcripts. In female *C. capitata* post-mating, 34 DEGs including *Obp19d* and chemoreception transcripts were recorded (Gomulski et al. 2012), and in *Ostrinia nibulalis* 978 DEGs including *peptidases*, *immune response* genes and *hormone receptors* were recorded (Al-Wathiqui et al. 2014). Despite such transcriptome studies, female post-mating changes are still poorly understood because of significant variation in the genes regulated, often low levels of differential expression in those genes which are differentially expressed, and/or the low number of functional genes regulated. The varied results and lack of information on functional genes necessitates that more studies in different systems are needed.

The *B. tryoni*-lure system, where lures regulate many genes in males (Kumaran et al. 2014b) and strongly promote female post-mating effects (Kumaran et al. 2013), provides a model system to capture deeper and behavior-specific transcriptional changes in females post-mating. Since lures modify refractoriness, fecundity and longevity in *B. tryoni*, it is likely that genes regulating those phenotypic changes will be expressed at a greater magnitude. To this end, we used RNA-seq analyses to carry out comparative transcriptome analysis of *B. tryoni* virgin females with females mated with non-lure fed males (=control-mated) and lure-fed males (=lure-mated). Mapping of genetic changes in females post-mating, especially in a pest species, is not only of importance to develop deeper understanding of mechanisms influencing female post-mating behaviors, but may also help with the pest management options, such as the Sterile Insect Technique (Klassen 2005).

## Materials and Methods

### Mating in B. tryoni

*Bactrocera tryoni* is a dusk mating species. In combined male and female aggregations, males commence rapid wing movements associated with dispersion of a pheromone at dusk when light intensity drops below a critical level, which elicits female response (Tychsen 1978; Ekanayake et al. 2017). Receptive females are subsequently mounted by males and copulation ensues. Males are mostly polygynous, whereas females were regarded as monandrous until recent empirical evidence confirmed that multiple mating, although not inevitable, is prevalent (Song et al. 2007; Radhakrishnan et al. 2009; Chinajariyawong et al. 2010). Although polyandry and polygyny is prevalent, mating occurs only once a day given the strict mating window.

### Insect Source and Experimental Groups

*Bactrocera tryoni* were obtained as pupae from the rearing facility at the [Queensland Government] Department of Agriculture Fisheries and Forestry, Brisbane. Adults emerging from pupae were provided with water, sugar, and protein hydrolysate *ad libitum* and maintained at 27 °C and 70% Relative Humidity in a room illuminated with natural light, in addition to fluorescent lighting between 0700 and 1,600 h every day.

Flies were sexed within 2–3 days of emergence, when still sexually immature, and the sexes were then housed separately in Perspex cages (30 × 30 × 30 cm). Three groups of females were maintained: mature virgin females (=“virgin females” hereafter), females mated with control males (=“control-mated females” hereafter) and females mated with males previously fed on the plant-derived secondary compound zingerone [4-(3-methoxy-4-hydroxyphenyl)-butan-2-one] (=“lure-mated females” hereafter). To obtain zingerone-fed males, flies (14 days old) were provided with 1.5 mL of zingerone (10 µg/µl of 95% ethanol, Sigma-Aldrich, CHEME, GmbH, Germany, >96% purity) on a cotton wick placed on an inverted petri dish for 2 h from 0800 to 1000 h on the day of mating. Our previous observations showed that males process zingerone within 3 h after feeding (Kumaran et al. 2014a). The concentrations, dilutions and presentation methods are based on previous studies on other *Bactrocera* flies (Shelly and Villalobos 1995; Hee and Tan 1998; Kumaran et al. 2013). To obtain mated females 50 females (14 days old) were housed in Perspex cages (30 × 30 × 30 cm) and either 50 control or lure-fed males were released at 1500 h, approximately 2 h before mating commences. Once mating commenced between 1700 and 1730 h, the mating pairs were transferred to new cages while ensuring that the flies remain paired. For virgin females, 50 females without males were maintained. Two cages per female group were maintained.

### RNA Isolation, Library Preparation, Assembly, and Annotation

From each of three groups (virgin females, control-mated females, and lure-mated females), totally 40 females were collected approximately 10–12 h after mating and snap-frozen in liquid nitrogen. Females were mated only once given the strict mating window. Although females in the cages were not monitored individually, our previous studies showed that lures do not modify copula duration (Kumaran et al. 2013). Total RNA from whole body was extracted using TRIzol and purified with a Qiagen RNeasy kit following the manufacturer’s instructions. Two replicates, each with RNA from 20 females pooled together, were maintained for each of the female groups. The quality of RNA was tested on 1.5% agarose gel as well as on a Agilent Bioanalyzer 2100, and only samples that had a RNA integrity number of >8 were used. Detailed methodology on RNA isolation, library construction, assembly and annotation are presented in our previous studies (Arthofer et al. 2014; Kumaran et al. 2014b; van der Burg et al. 2016; Stewart et al. 2017). Low quality leading and trailing bases (<Q30) were trimmed and adapter sequences removed from each sequence read using Trimmomatic (Bolger et al. 2014). The final transcriptome was assess for completeness using BUSCO (Simão et al. 2015). The raw sequence files are deposited in the Sequence Read Archive accessions SRR5927915 (virgin 1), SRR5927916 (virgin 2), SRR5927913 (normal-mated 1), SRR5927914 (normal-mated 2), SRR5927911 (lure-mated 1), and SRR5927912 (lure-mated 2) in NCBI.

### Analysis of Differentially Expressed Genes

To determine differentially expressed genes (DEGs), sequencing reads were mapped to contigs using bowtie2 and Fragments Per kb per Million fragments (FPKM) values were used to determine expression levels and differential gene expression for all the comparisons *viz*., virgin versus control-mated, virgin versus lure-mated, and control-mated versus lure-mated using the trinity platform (Haas et al. 2013) using version 3.4.0 of the BioConductor package edgeR (Robinson et al. 2010). Transcripts with an FPKM value of zero were excluded from downstream analysis. A False discovery rate (FDR) of ≤0.001 and log two fold change of ≥2 was used to determine statistically significant differential expression in virgin versus control-mated versus lure-mated females. We undertook gene set enrichment analysis to determine whether particular Gene Ontology (GO) categories were overrepresented in up- and down-regulated DEGs using GOSeq (Young et al. 2010).

## Results

### Sequence Assembly Statistics

The total number of reads generated for all libraries was 490,218, 344. The number of reads generated per library was as follows: lure-mated 1 = 74,256, 386; lure-mated 2 = 53,599,704; control-mated 1 = 59,549,082; control-mated 2 = 90,701,413; virgin 1 = 66,654,335; virgin 2 = 65,411,462. The raw reads are deposited in the SRA accessions SRR5927912-SRR5927916 under BioProject PRJNA397485 and BioSample SAMN07459442 in NCBI. The total number of contigs generated in the combined assembly (before reducing redundancy and bias with CD-HIT) was 108,313. After CD-HIT, 67,492 contigs (transcripts) remained with a total of 58,291 trinity genes. The average contig length was 765.3 bp, the contig N50 was 1,420 and the percentage GC content was 38.8%. Our transcriptome was largely complete with >95% of the 1,658 core insect genes present as full length copies in the assembly (BUSCO benchmarking = C: 95.5%[S: 63.1%, D: 32.4%], F: 2.3%, M: 2.2%, n: 1,658).

### DEGs in Control-Mated Females Versus Virgin Females

**Fig. 1.**
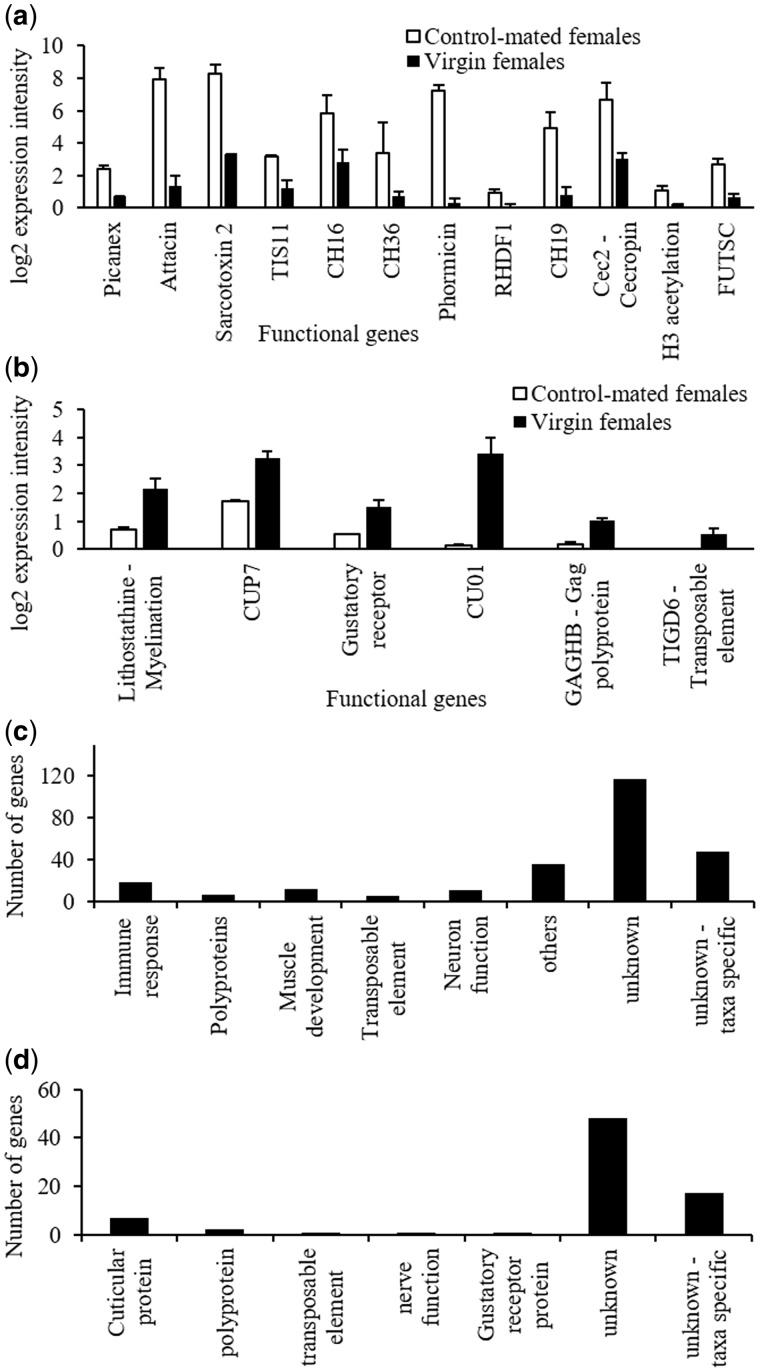
—Differential expression in control-mated females compared with virgin females. (*a*) Example up-regulated transcripts and their expression levels; (*b*) Example down-regulated functional transcripts and their expression levels; (*c*) Up-regulated genes within functional classes differentially expressed; and (*d*) Down-regulated genes within functional classes differentially expressed.

Three-hundred and thirty-one genes were differentially expressed (DEGs) (254 up-regulated and 77 down-regulated) in control-mated females when compared with virgin females (supplementary tables S1 and S2, Supplementary Material online). Among the functional transcripts up-regulated, immune response transcripts were highly enriched followed by genes encoding chorion proteins, transposable elements, *titin*-like muscle proteins and histone proteins (fig. 1*a*). The most significantly down-regulated genes in mated females encoded cuticle proteins, gustatory receptor transcript, polyprotein and a transposable element (fig. 1*b*). The majority of DEGs (165 out of 254 up-regulated and 60 out of 77 down-regulated) had unknown functions; and approximately 30% of the up-regulated and 25% of the down-regulated genes are known only from tephritid fruit flies (*B. dorsalis* and/or *C. capitata*) (fig. 1*c* and *d*). The difference in expression intensity in DEGs was up to 11 fold for up- and down-regulated transcripts (supplementary fig. S1, Supplementary Material online).

Gene Ontology (GO) analysis revealed that up-regulated DEGs were represented in 194 biological processes (supplementary table S3, Supplementary Material online), 33 molecular function (supplementary table S4, Supplementary Material online) and 51 cellular component GO terms (supplementary table S5, Supplementary Material online). Most significantly enriched GO terms corresponded with DEGs and comprised mostly of defence (immune) response transcripts and muscle functions (table 1). The suppressed DEGs were represented in 98 GO terms in control-mated females when compared with virgin females (supplementary table S6, Supplementary Material online). Most significantly suppressed GO terms included transcripts encoding metabolic processes, biosynthetic processes, transporter activities, and catalytic activities (table 2).

**Table 1:** Most Significant Up-Regulated GO Terms in Control-Mated Females Compared with Virgin Females (full list appended as supplementary file, Supplementary Material online)

GO Id.	GO Term	Total Numbers in GO Term	Numbers Differentially Expressed	*P* Value
*Biological Processes*				
GO: 0007062	Sister chromatid cohesion	35	10	0
GO: 0007076	Mitotic chromosome condensation	37	10	0
GO: 0007522	Visceral muscle development	19	10	0
GO: 0016203	Muscle attachment	37	10	0
GO: 0042742	Defense response to bacterium	184	19	2.97E-17
GO: 0009617	Response to bacterium	200	19	1.26E-16
GO: 0045087	Innate immune response	257	21	7.23E-16
GO: 0045214	Sarcomere organization	60	14	5.93E-15
GO: 0098542	Defense response to other organism	241	19	1.08E-14
GO: 0051707	Response to other organism	342	21	3.47E-14
*Molecular function*				
GO: 0008307	Structural constituent of muscle	40	12	1.69E-12
GO: 0003779	Actin binding	195	15	1.63E-09
GO: 0008092	Cytoskeletal protein binding	395	17	1.6E-07
GO: 0005198	Structural molecule activity	520	17	2.59E-07
GO: 0016709	Oxidoreductase activity	11	3	0.000172
GO: 0001872	(1→3)-beta-D-glucan binding	5	2	0.000343
GO: 0043914	NADPH: sulfur oxidoreductase activity	3	2	0.001285
GO: 0004521	Endoribonuclease activity	137	5	0.001533
GO: 0004540	Ribonuclease activity	156	5	0.002875
GO: 0004523	RNA-DNA hybrid ribonuclease activity	105	4	0.003011
*Cellular Processes*				
GO: 0000794	Condensed nuclear chromosome	31	10	0
GO: 0005859	Muscle myosin complex	23	11	0
GO: 0005863	Striated muscle myosin thick filament	17	10	0
GO: 0016460	Myosin II complex	28	11	0
GO: 0031674	I band	23	11	0
GO: 0032982	Myosin filament	30	11	0
GO: 0036379	Myofilament	26	11	0
GO: 0005576	Extracellular region	790	29	1.67E-15
GO: 0030017	Sarcomere	40	13	1.93E-14
GO: 0044449	Contractile fiber part	122	17	6.96E-12

**Table 2:** Most Significant Down-Regulated GO Terms in Control-Mated Females Compared with Virgin Females (full list appended as supplementary file, Supplementary Material online)

GO Id.	GO Term	Total Numbers in GO Term	Numbers Differentially Expressed	*P* Value
*Biological Processes*				
GO: 0051246	Regulation of protein metabolic process	807	0	0.000975
GO: 0031323	Regulation of cellular metabolic process	2,804	9	0.00132
GO: 0032268	Regulation of cellular protein metabolic process	756	0	0.001448
GO: 0044248	Cellular catabolic process	870	0	0.002026
GO: 0044711	Single-organism biosynthetic process	903	0	0.002672
GO: 0080090	Regulation of primary metabolic process	2,660	9	0.002726
GO: 0009056	Catabolic process	1,118	1	0.003689
GO: 0019538	Protein metabolic process	2,370	8	0.004911
GO: 0033036	Macromolecule localization	901	1	0.005483
GO: 0006811	Ion transport	784	0	0.006089
*Molecular Function*				
GO: 0003674	Molecular function	11,752	62	7.42E-07
GO: 0022892	Substrate-specific transporter activity	883	0	0.002882
GO: 0005215	Transporter activity	1,072	1	0.005253
GO: 0022891	Transmembrane transporter activity	791	0	0.006853
GO: 0015075	Ion transmembrane transporter activity	696	0	0.012545
GO: 0003824	Catalytic activity	5,755	28	0.015134
GO: 0022857	Transmembrane transporter activity	915	1	0.020015
GO: 0043167	Ion binding	4,881	25	0.021984
GO: 0005509	Calcium ion binding	444	0	0.026681
GO: 0005488	Binding	8,977	54	0.037584
*Cellular Component*				
GO: 0016020	Membrane	3,753	12	0.000259
GO: 0043227	Membrane-bounded organelle	5,567	23	0.000416
GO: 0044464	Cell part	10,245	57	0.00056
GO: 0043231	Intracellular membrane-bounded organelle	5,261	23	0.002085
GO: 0005886	Plasma membrane	1,655	3	0.002407
GO: 0031982	Vesicle	992	1	0.003442
GO: 0005737	Cytoplasm	2,727	10	0.004576
GO: 0031988	Membrane-bounded vesicle	933	1	0.005007
GO: 0005634	Nucleus	3,432	13	0.005385
GO: 0005739	Mitochondrion	763	0	0.006402

### DEGs in Lure-Mated Females Versus Virgin Females

**Fig. 2.**
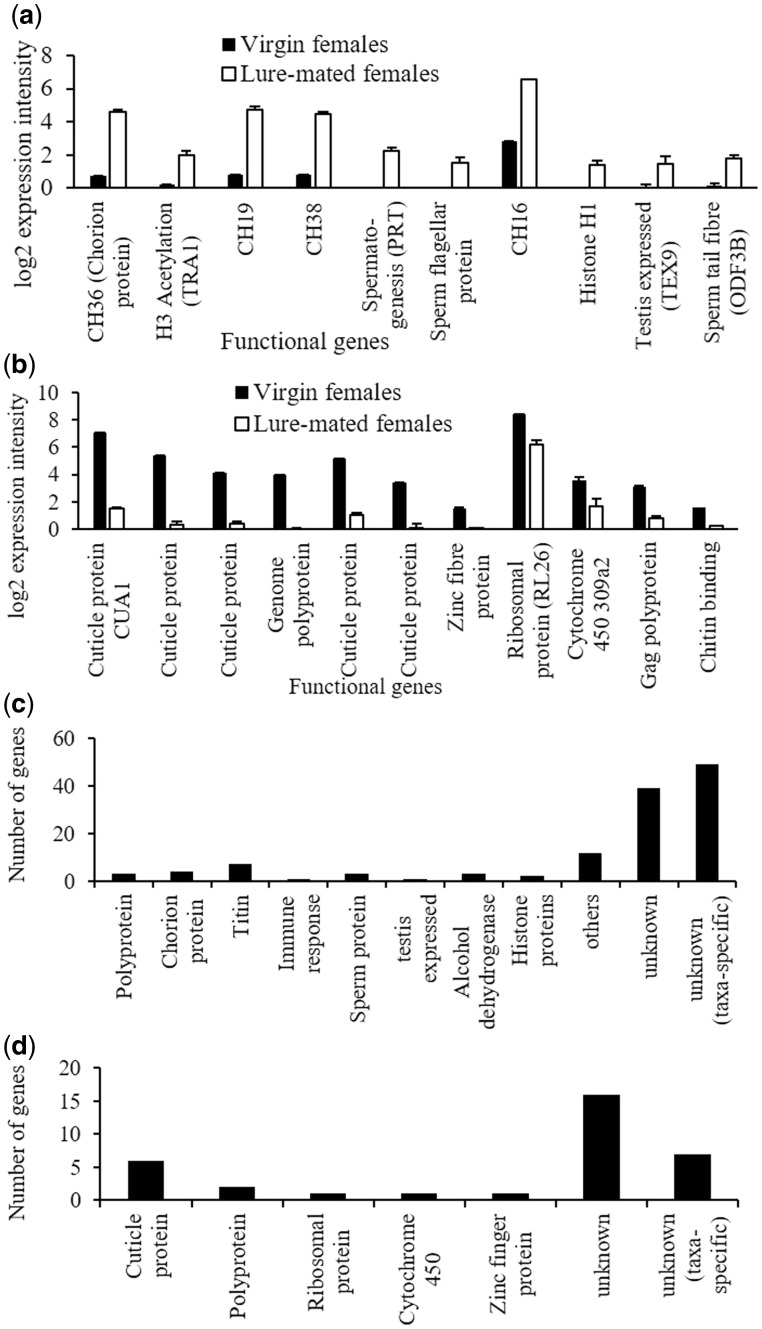
—Differential expression in lure-mated females compared with virgin females. (*a*) Example up-regulated transcripts and their expression levels; (*b*) Example down-regulated functional transcripts and their expression levels; (*c*) Up-regulated genes within functional classes differentially expressed; and (*d*) Down-regulated genes within functional classes differentially expressed.

We found 124 genes up-regulated and 34 genes down-regulated in lure-mated females when compared with virgin females (supplementary tables S7 and S8, Supplementary Material online), with several fold difference in expression intensities (supplementary fig. S2, Supplementary Material online). The DEGs were dissimilar to that of control-mated females, with 67 new up-regulated transcripts identified. In addition to genes encoding chorion and *titin* proteins, DEGs in lure-mated females comprised testis expressed proteins, sperm proteins, and a different set of histone proteins (fig. 2*a*). Further, fewer immune response genes were over-expressed in lure-mated females than in control-mated females: of the 124 up-regulated genes, only two genes were immune related compared with 16 (out of 254) in control-mated females. Additionally, 13 genes were switched off only in lure-mated females: these were not differentially expressed in control-mated females. The down-regulated transcripts that encoded proteins with known functions were cuticle proteins, polyproteins, ribosomal protein (*RL26*), and *cytochrome 450 309a2* (fig. 2*b*). Several taxa-specific transcripts with unknown functions were also differentially expressed in lure-mated females (fig. 2*c* and *d*).

One-hundred and seventy-nine GO terms in the biological processes category were significantly enriched (*P* < 0.05) with muscle development, DNA packaging, and chromosome condensation being the most significantly enriched terms (table 3 and supplementary table S9, Supplementary Material online). In the cellular component category, 38 GO terms showed significant enrichment and the most significantly enriched terms were muscle filament, sarcomere, myosin filament, and condensed chromosome (table 3 and supplementary table S10, Supplementary Material online). There were 28 GO terms enriched in the molecular function category, which included muscle structural constituents, actin binding, and enzymatic activities (table 3 and supplementary table S11, Supplementary Material online). Forty GO terms were down-regulated in lure-mated females with regulation of metabolic processes, signal transduction, and negative regulation of biological processes being the most significantly down-regulated GO terms (table 4 and supplementary table S12, Supplementary Material online).

**Table 3:** Most Significantly Up-Regulated GO Terms in Lure-Mated Females Compared with Virgin Females

GO Id.	GO Term	Total Numbers in GO Term	Numbers Differentially Expressed	*P* Value
*Biological processes*				
GO: 0007522	Visceral muscle development	19	8	2.38E-12
GO: 0030261	Chromosome condensation	54	9	2.06E-11
GO: 0006323	DNA packaging	55	9	2.19E-11
GO: 0016203	Muscle attachment	37	8	1.26E-10
GO: 0007062	Sister chromatid cohesion	35	8	1.34E-10
GO: 0007076	Mitotic chromosome condensation	37	8	1.58E-10
GO: 0007520	Myoblast fusion	56	8	7.51E-10
GO: 0007519	Skeletal muscle tissue development	54	8	8.58E-10
GO: 0035206	Regulation of hemocyte proliferation	54	8	1.35E-09
GO: 0000768	Syncytium formation by plasma membrane fusion	65	8	1.56E-09
*Cellular Component*				
GO: 0005863	Striated muscle myosin thick filament	17	8	0
GO: 0030017	Sarcomere	40	10	0
GO: 0005859	Muscle myosin complex	23	8	4.85E-11
GO: 0031674	I band	23	8	5.07E-11
GO: 0032982	Myosin filament	30	8	5.75E-11
GO: 0036379	Myofilament	26	8	7.57E-11
GO: 0000794	Condensed nuclear chromosome	31	8	1.47E-10
GO: 0016460	Myosin II complex	28	8	2.15E-10
GO: 0000793	Condensed chromosome	39	8	4.00E-10
GO: 0044449	Contractile fiber part	122	12	3.58E-09
*Molecular Function*				
GO: 0008307	Structural constituent of muscle	40	8	1.57E-08
GO: 0003779	Actin binding	195	10	1.39E-07
GO: 0005198	Structural molecule activity	520	10	4.41E-06
GO: 0016763	Transferase activity, transferring pentosyl groups	27	3	1.73E-05
GO: 0008092	Cytoskeletal protein binding	395	10	1.91E-05
GO: 0003796	Lysozyme activity	12	2	0.000157
GO: 0004568	Chitinase activity	29	2	0.001118
GO: 0017061	S-methyl-5-thioadenosine phosphorylase activity	3	1	0.004963
GO: 0002060	Purine nucleobase binding	3	1	0.005099
GO: 0004731	Purine-nucleoside phosphorylase activity	3	1	0.005099

**Table 4:** Most Significant GO Terms Down-Regulated in Lure-Mated Females Compared with Virgin Females

GO Id.	GO Term	Total Numbers in GO Term	Numbers Differentially Expressed	*P* Value
*Biological Processes*				
GO: 0031323	Regulation of cellular metabolic process	2,804	1	0.000548
GO: 0060255	Regulation of macromolecule metabolic process	2,773	1	0.000557
GO: 0080090	Regulation of primary metabolic process	2,660	1	0.00085
GO: 0007165	Signal transduction	1,883	0	0.00187
GO: 0048519	Negative regulation of biological process	2,116	1	0.002111
GO: 0019222	Regulation of metabolic process	3,216	3	0.002131
GO: 0048523	Negative regulation of cellular process	1,911	1	0.004281
GO: 0010468	Regulation of gene expression	2,204	1	0.006921
GO: 0009653	Anatomical structure morphogenesis	1,407	0	0.008501
GO: 0051171	Regulation of nitrogen compound metabolic process	2,127	1	0.008567
*Cellular Component*				
GO: 0016020	Membrane	3,753	4	0.006526
GO: 0043227	Membrane-bounded organelle	5,567	8	0.006829
GO: 0043231	Intracellular membrane-bounded organelle	5,261	8	0.016174
GO: 0044425	Membrane part	3,809	5	0.038189
GO: 0005886	Plasma membrane	1,655	1	0.048329
*Molecular Function*				
GO: 0043169	Cation binding	3,480	3	0.004204
GO: 0046872	Metal ion binding	3,420	3	0.004734
GO: 0043167	Ion binding	4,881	8	0.019285

### DEGs in Lure-Mated Females Versus Control-Mated Females

When lure-mated females were compared with control-mated females, 89 DEGs were detected: 70 up-regulated and 19 down-regulated (supplementary tables S13 and S14, Supplementary Material online). The expression intensity was low with up to 7-fold difference (supplementary figs. S3 and S4, Supplementary Material online), compared with 11-fold difference recorded in control-mated versus virgin or lure-mated versus virgin categories. Testis expressed, sperm, farnesol dehydrogenase, reverse transcriptase, and histone 1 to histone 5 linker genes were up-regulated (supplementary fig. S5*a*, Supplementary Material online); odorant binding (*Obp56A*), occluding homology, binding, nuclear transport, and transposable elements were the down-regulated transcripts (supplementary fig. S5*b*, Supplementary Material online). A number of differentially expressed transcripts with unknown function were detected and most of these were taxa-specific, having no significant BLAST hit to any other species in the current databases (supplementary fig. S5*c* and *d*, Supplementary Material online).

**Fig. 3.**
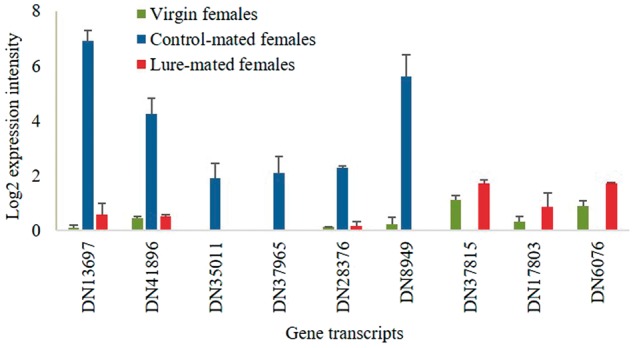
—The functional transcripts showed reversal in expression pattern in lure-mated females. There were six genes enriched after control-mating but suppressed in lure-mated females, and there were three genes suppressed after control-mating but enriched in lure-mated females.

Most of the DEGs in lure-mated females (e.g., sperm proteins, testis expressed proteins) were only differentially expressed (67 out of 70 up-regulated and 13 out of 19 down-regulated) in lure-mated females; those genes were not differentially expressed in control-mated females when compared with virgin females. There were 54 unknown function transcripts up-regulated only in lure-mated females, and 31 of them were specific to tephritids (supplementary fig. S4, Supplementary Material online). The genes suppressed only in lure-mated females included *Obp56A*, occudin homology domain, carboxylate reductase, and binding nuclear protein, with nine unknown function transcripts (supplementary fig. S4, Supplementary Material online). The remaining six down-regulated genes and the three up-regulated genes showed reversal in expression, that is, those six genes were up-regulated and the three genes down-regulated, respectively, in control-mated females (fig. 3).

One-hundred and twenty-six GO terms were enriched within biological processes category with metabolic processes, biosynthetic processes, and cellular metabolic compound salvage being the most significantly enriched terms (table 5 and supplementary table S15, Supplementary Material online). Within cellular component category, six GO terms showed significant enrichment with the most significant being nucleosome, DNA bending, and DNA packing complex (table 5). There were 37 GO terms enriched within molecular function category with transferase activity, transferring pentosyl groups, and enzymatic activities most significantly enriched among other GO terms (table 5 and supplementary table S16, Supplementary Material online). Thirteen GO terms with nine biological processes, three cellular component, and one molecular function category were significantly down-regulated in lure-mated females (table 6).

**Table 5:** Most Significant Up-Regulated GO Terms in Lure-Mated Females Compared with Control-Mated Females (full list appended as supplementary file, Supplementary Material online)

GO Id.	GO Term	Total Numbers in GO Term	Numbers Differentially Expressed	*P* Value
*Biological Processes*				
GO: 0043101	Purine-containing compound salvage	15	2	0.00013
GO: 0046128	Purine ribonucleoside metabolic process	120	3	0.000338
GO: 0042278	Purine nucleoside metabolic process	126	3	0.000389
GO: 0072522	Purine-containing compound biosynthetic process	128	3	0.000416
GO: 0009119	Ribonucleoside metabolic process	142	3	0.000558
GO: 0009116	Nucleoside metabolic process	156	3	0.00073
GO: 0043094	Cellular metabolic compound salvage	38	2	0.000847
GO: 1901657	Glycosyl compound metabolic process	170	3	0.000932
GO: 0019523	L-idonate metabolic process	1	1	0.001442
GO: 0046176	Aldonic acid catabolic process	1	1	0.001442
*Molecular Function*				
GO: 0016763	Transferase activity, transferring pentosyl groups	27	2	0.000447
GO: 0017061	S-methyl-5-thioadenosine phosphorylase activity	3	1	0.00338
GO: 0002060	Purine nucleobase binding	3	1	0.003383
GO: 0004731	Purine-nucleoside phosphorylase activity	3	1	0.003383
GO: 0042301	Phosphate ion binding	4	1	0.004507
GO: 0002054	Nucleobase binding	4	1	0.004648
GO: 0008422	Beta-glucosidase activity	9	1	0.010515
GO: 0004553	Hydrolase activity, hydrolyzing O-glycosyl compounds	147	2	0.012497
GO: 0003796	Lysozyme activity	12	1	0.013456
GO: 0016798	Hydrolase activity, acting on glycosyl bonds	162	2	0.015102
*Cellular Component*				
GO: 0000786	Nucleosome	17	2	0.000192
GO: 1990104	DNA bending complex	17	2	0.000192
GO: 0044815	DNA packaging complex	22	2	0.000352
GO: 0032993	Protein-DNA complex	33	2	0.0007
GO: 0045261	Proton-transporting ATP synthase complex, catalytic core F(1)	12	1	0.01361
GO: 0033178	Proton-transporting two-sector ATPase complex, catalytic domain	33	1	0.037039

**Table 6:** Most Significant Down-Regulated GO Terms in Lure-Mated Females Compared with Control-Mated Females (full list appended as supplementary file, Supplementary Material online)

GO Id.	GO Term	Total Numbers in GO Term	Numbers Differentially Expressed	*P* Value
GO: 0050789	Regulation of biological process (BP)	5,387	1	0.00232
GO: 0050794	Regulation of cellular process (BP)	5,044	1	0.00422
GO: 0065007	Biological regulation (BP)	5,805	2	0.007141
GO: 0005515	Protein binding (MF)	3,223	0	0.009313
GO: 0019222	Regulation of metabolic process (BP)	3,216	0	0.010203
GO: 0044464	Cell part (CC)	10,245	8	0.014812
GO: 0031323	Regulation of cellular metabolic process (BP)	2,804	0	0.020083
GO: 0060255	Regulation of macromolecule metabolic process (BP)	2,773	0	0.020886
GO: 0080090	Regulation of primary metabolic process (BP)	2,660	0	0.025073
GO: 0032502	Developmental process (BP)	3,787	1	0.030784
GO: 0005575	Cellular_component (CC)	11,673	11	0.036444
GO: 0044424	Intracellular part (CC)	8,928	7	0.041381
GO: 0044767	Single-organism developmental process (BP)	3,523	1	0.0436

Across all three conditions (virgin, control-mated, and lure-mated), 434 genes were differentially expressed (supplementary fig. S6, Supplementary Material online); among, 80,214 and 83 genes were uniquely up-regulated in virgin, control-mated and lure-mated females respectively. There were 69 DEGs found in both lure-mated and control-mated females with 47 up-regulated and 20 down-regulated transcripts. Up-regulated functional genes included *titin* like muscle proteins, chorion proteins, Nesprin1, replicase polyprotein, and H3 acetylation. Down-regulated genes included adult cuticle protein, genome polyprotein, zinc finger protein, and chitin binding protein.

## Discussion

### Summary

Our study has revealed 331 DEGs in control-mated females when compared with virgin females. Up-regulated genes were mostly transcripts governing immune response functions, as previously documented in *D. melanogaster* and *D. mojavensis* (Lawniczak and Begun 2004; McGraw et al. 2004; Bono et al. 2011). Within tephritids, post-mating up-regulation of several immune genes (*cecropin, sapecin, attacin, defensin*, and *diptericin*) was reported in *B. dorsalis* (Wei et al. 2016; Zheng et al. 2016), but none were reported for *C. capitata* (Gomulski et al. 2012). It is possible that the female immune system has responded to the reception of Acps, sperm, and associated contaminants from males. Genes encoding chorion proteins were the next dominant transcripts up-regulated in mated females, and this result contradicts McGraw et al. (2008) who found suppression of CH36 and CH38 in *D. melanogaster*.

Among the DEGs in lure-mated females, 50% (67 up-regulated and 13 down-regulated) were unique transcripts differentially expressed only in lure-mated females, and these were not differentially expressed when control-mated females were compared with virgin females. This suggests that mating with zingerone-fed males is indirectly modulating the expression pattern of a set of genes not observed in previous studies. Some of the unique transcripts in lure-mated females encode histone proteins, testis expressed proteins and sperm proteins, with very few immune response genes and no immune response GO terms. Such an observation may indicate that mating with lure-fed males does not elicit a strong immune response in females and may help to explain female preference for lure-fed males as “immunity” can be under sexual selection (Lawniczak et al. 2007). While it is unknown if lures reduced contamination in male Acps or ejaculates, lure-fed males are physically fitter than unfed males (Kumaran et al. 2014b), and it is yet to be investigated whether lures help male *B. tryoni* to burn unwanted fats and associated contaminations. With respect to sperm and testis expressed genes, expression is possibly from the sperm stored in the spermatheca as *B. tryoni* females store sperm for at least 15 days after mating (Perez‐Staples et al. 2007) and this observation perhaps explains the reduced refractoriness observed in lure-mated females (Kumaran et al. 2013). However, the reason for the expression of male-biased testis expressed genes in mated females remains unclear.

We *a priori* expected DEGs detected in both control-mated and lure-mated females (78 out of 158 in lure-mated females) to differ in their expression intensities (i.e., with higher expression in lure-mated females), which we hoped would shed light on the female specific genes mediating fecundity and refractoriness. However, no difference in expression intensity was observed except for nine genes which, paradoxically, showed reversal in expression (fig. 3). Overall, our results indicate the possibility of the previously unobserved, but differentially expressed genes to be involved in mediating fecundity and refractoriness. It is highly likely that the DEGs found in the mated females were regulated exclusively by mating; however, the possibility that the lure-fed males could have modified the close-range courtship behaviors (e.g., harassment and intrasex competition) and underlying transcription factors in females (during precopulatory interactions) cannot be excluded.

The number of DEGs detected following mating (331 from control-mated females plus 80 new DEGs from lure-mated females) were substantially greater than the 32 transcripts reported in *C. capitata* (Gomulski et al. 2012) and the 83 in *B. dorsalis* (Zheng et al. 2016), but only approximately one-fifth of the number reported in *Drosophila* (McGraw et al. 2004). Although the magnitude of DEGs we detected was greater (up to an 11-fold expression difference) compared with a 2-fold differences reported in *D. melanogaster* and *C. capitata*, these differences can be attributed to analytical methodology used (RNA-seq vs. microarray) rather than a difference among species. In addition, although our replicates are pooled samples of 20 females, it is possible that two replicates still could have underestimated the number of DEGs in *B. tryoni* (Schurch et al. 2016). Despite the methodological differences, the additional unique transcripts recorded in *B. tryoni* can help understand the female factors possibly mediating the post-mating changes. In addition to the already reported immune genes, sperm proteins, and testis expressed genes, our study has revealed expression of several *titin* like muscle proteins and histone proteins and we elaborate on these below.

### Transcript Homologues Regulating Mating and/or Remating

Mating response homologues *takeout, Ca(2+)/calmodulin-responsive adenylate cyclase, protein yellow, ejaculatory bulb specific protein, lingerer, sarah, fruitless*, and *sex peptide receptor* did not show differential expression in either control-mated or lure-mated females. We expected these genes to be suppressed in lure-mated females at a greater magnitude compared with control-mated females, since we found greater refractoriness in lure-mated females (Kumaran et al. 2013). It is possible that these genes are only transcribed during the narrow temporal window of dusk, when copulation occurs in *B. tryoni* (Ekanayake et al. 2017). The other possible explanation is that there are sex specific differences in the expression of these genes, with greater representation of mating transcripts in males compared with females: most of the mating related genes listed above were over-represented in male *B. tryoni* (Kumaran et al. 2014b). These mating response homologues were also not reported as DEGs in female *Drosophila* and other species which supports the sex specific expression hypothesis (McGraw et al. 2008; Gomulski et al. 2012; Zheng et al. 2016).

Odorant binding protein *Obp56a* was down-regulated in mated *B. tryoni* females, as noticed in *D. melanogaster* (McGraw et al. 2004), with greater suppression in lure-mated females (supplementary fig. S8, Supplementary Material online). It is possible that down regulation of odorant binding proteins suppress female attraction or receptiveness towards courting males, ultimately controlling remating frequency.

#### Titin-*like Muscle Proteins*

We recorded several other transcripts up-regulated after mating that are unique to this study, including *titin*-like proteins and histone proteins. *Titin* (=connectin) was not reported previously either in *Drosophila* or tephritids, although a few muscle related proteins were differentially expressed in *Ostrinia nubilalis* (Al-Wathiqui et al. 2014). *Titin* is found primarily in skeletal muscles and is involved in sarcomere related functions (Greaser 2001). It contains a protein kinase domain positioned to sense mechanical load and it is found that the kinase domain interacts with the zinc-finger proteins to respond to mechanical stimuli in humans (Lange et al. 2005). The role of these muscle proteins in mated female *B. tryoni* is not known; given that males remain mounted on females during several hours of copulation (Kumaran et al. 2014b), perhaps those proteins were activated to hold the male weight. Since there was no previous record in insects, it is also possible that *titin* encode differential functions unlike in humans.

### Histone Proteins

There were two transcripts encoding histone protein analogues (H1 and H5 linker) overexpressed only in lure-mated females, and a H3 acetylation transcript was overexpressed in both female types. There was a greater expression of H3 acetylation in lure-mated females, which along with the expression of H1 and H5 linker, suggests possible epigenetic changes (histone modifications) post-mating in *B. tryoni*. Differential expression of histone transcripts has not been reported previously in any model organisms or tephritids; however, targeted epigenetic studies have identified histone modifications in females post-mating (Zhou et al. 2014). Histones function to package DNA into nucleosomes and it is a main protein within chromatin. Since DNA wraps around histones, they play a role in gene regulation by altering chromatin structure (Grunstein 1997). Acetylation of histone H3 occurs at several different lysine positions in the histone tail and is performed by the enzymes called histone acetyltransferases (HATs) (Bannister and Kouzarides 2011). For instance, H3 acetylation localized to discrete sites in the mammalian genome mediate distinct chromatin functions that dictate transgene expression or silencing (Yan and Boyd 2006). Epigenetic variations are reported to mediate individual differences in behavior and such variations have often been traced through several generations (Jensen 2013; Dias et al. 2015). In *B. tryoni* males, the “lure foraging” trait was found to be passed onto offspring sired by males fed on phytochemicals (Kumaran and Clarke 2014) suggesting possible phytochemical mediated epigenetic changes.

### Taxa-Specific Transcripts

There were a great number of transcripts with unknown functions and most of these are predicted proteins that have only previously been identified within other tephritid fruit fly genomes. The reason for the failure to assign functions is perhaps because female specific transcriptome resources are generally lacking for most tephritid species, or that they are restricted to this group for which genomic resources have only recently become available. The functional role of taxa-specific transcripts needs to be investigated as they may be involved in mediating post-mating behaviors such as oviposition and remating.

### Female-Specific Factors of Post-mating Behaviors

Although Acps are the proximate mechanisms of female postmating changes, we strongly believe that females are also likely to control their post-mating patterns to some extent for the following reasons: 1) behaviorally, females choose whether to mate or not, and with whom in most of the species (Andersson and Simmons 2006); and 2) mating, egg production, and oviposition is physiologically costly (Baer and Schmid‐Hempel 2001; Colegrave et al. 2002; Wigby and Chapman 2005), thus selection should act on females to utilise their resources optimally to maximize reproductive success. This hypothesis does not compete with the effect of male Acps in mediating female behaviors, but insist additional factors need to be studied to fully understand complex female post-mating behaviors (Immonen and Ritchie 2012). The transcriptomic changes found in *B. tryoni* and other systems will be highly useful resources for future studies targeting female-specific factors.

## Conclusion

This study presents a wide range of functional transcripts differentially regulated in mated females. Transcripts such as those encoding *titin*-like muscle proteins, histone proteins and a number of unknown genes in mated females suggest their possible role in mediating post-mating changes and warrants further research on female-specific genetic changes. Exclusively enriched or suppressed genes in lure-mated females and taxa-specific transcripts suggest complex factors, additional to male Acps and sperm, contribute to female post-mating behaviors. Further targeted studies on differentially expressed genes, not regulated by male Acps, could shed more light on understanding the evolutionary implications of post-mating changes and female factors mediating those changes.

## Supplementary Material

Supplementary data are available at *Genome Biology and Evolution* online.

## Supplementary Material

Supplementary Figures and Tables
