# Supplementary figures and images for: Plant-Mediated Female Transcriptomic Changes Post-Mating in a Tephritid Fruit Fly, Bactrocera tryoni
Source: Genome Biol Evol. 2017 Dec 6;10(1):94–107. doi: 10.1093/gbe/evx257 (PMC5765559; doi:10.1093/gbe/evx257)

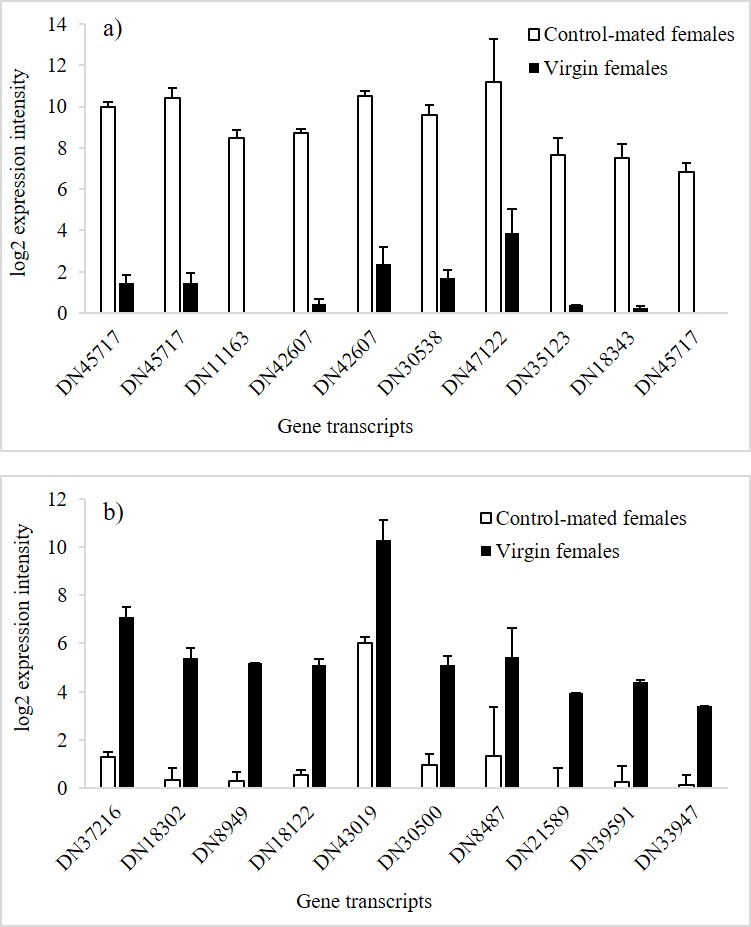

Supplement: Supplementary Figures and Tables [file evx257_supp.zip › Supplementary Figure S1.jpg]

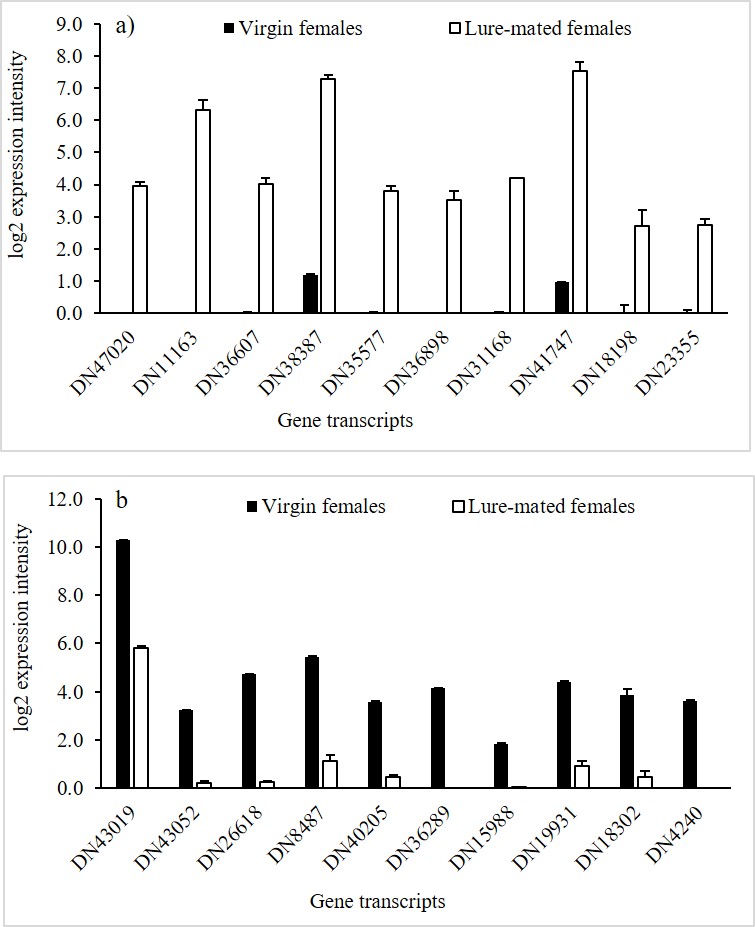

Supplement: Supplementary Figures and Tables [file evx257_supp.zip › Supplementary Figure S2.jpg]

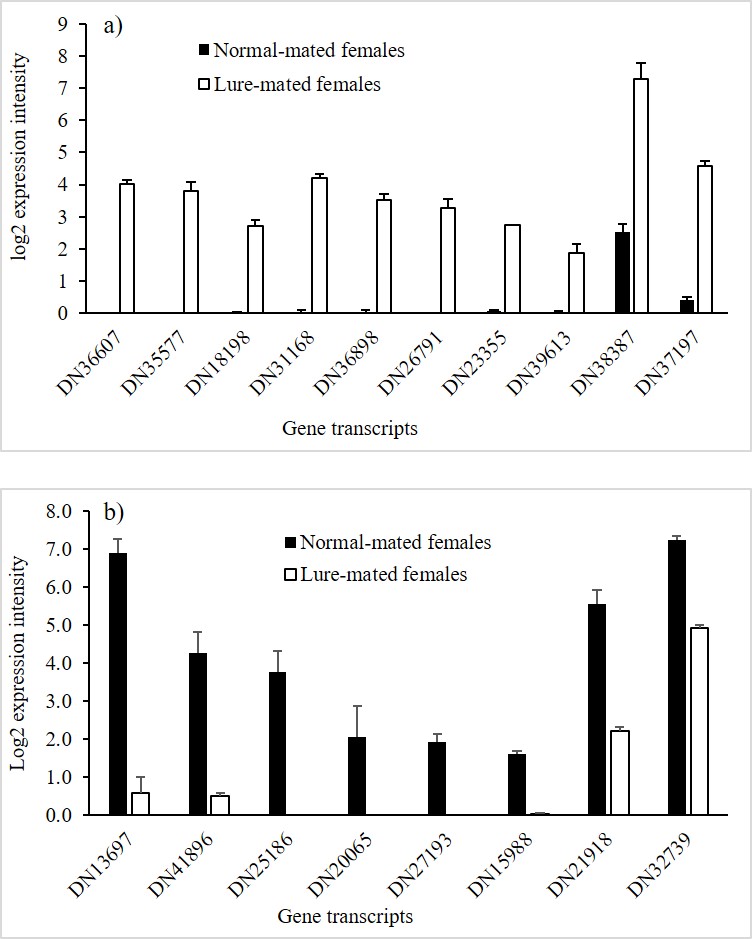

Supplement: Supplementary Figures and Tables [file evx257_supp.zip › Supplementary Figure S3.jpg]

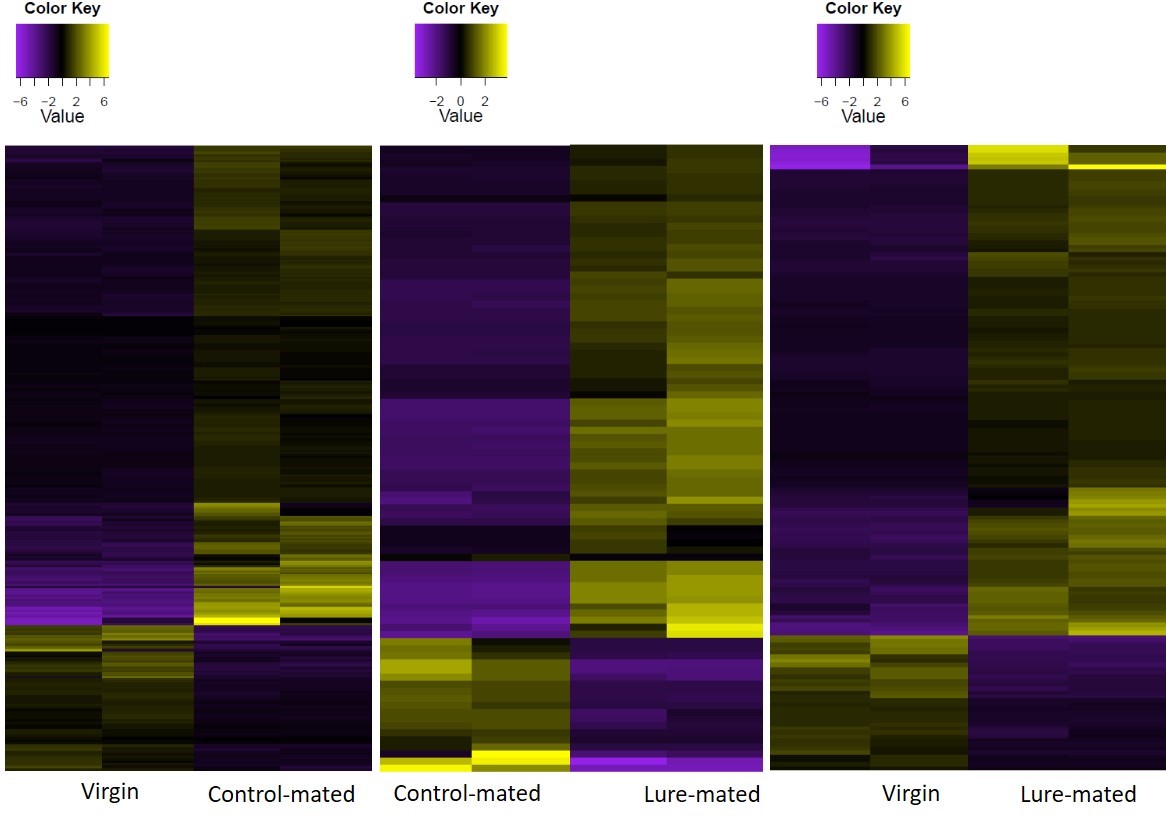

Supplement: Supplementary Figures and Tables [file evx257_supp.zip › Supplementary Figure S4.jpg]

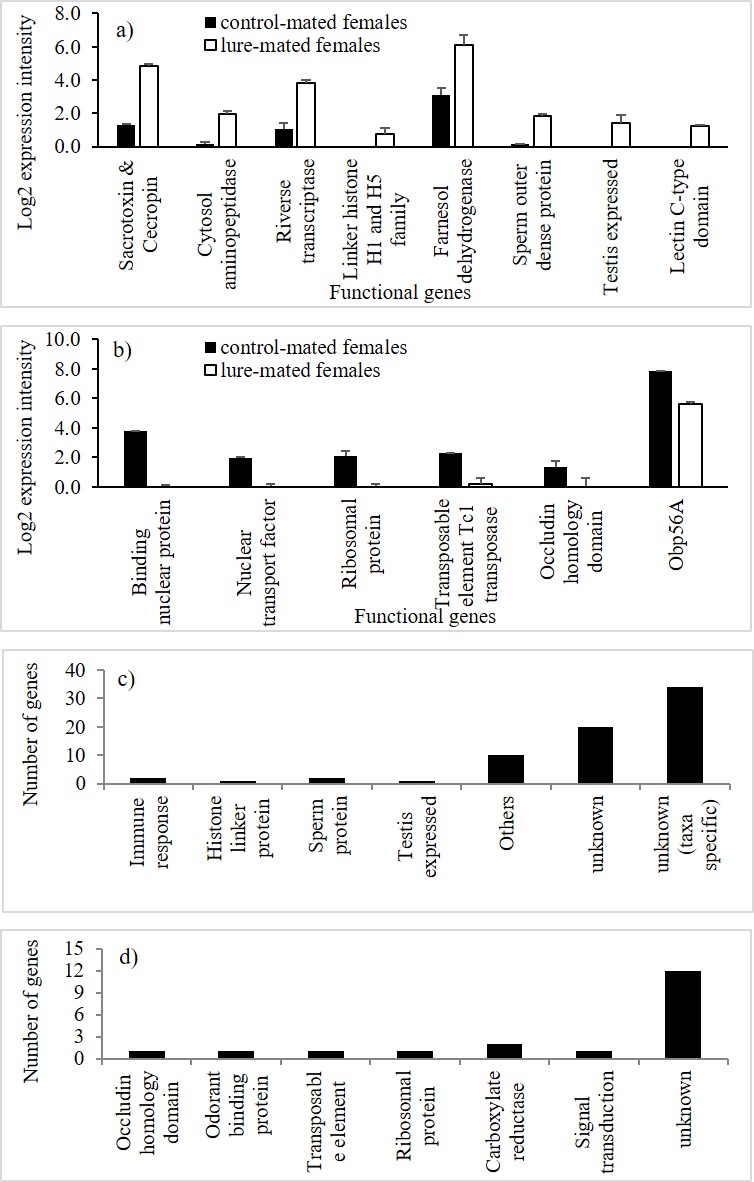

Supplement: Supplementary Figures and Tables [file evx257_supp.zip › Supplementary Figure S5.jpg]

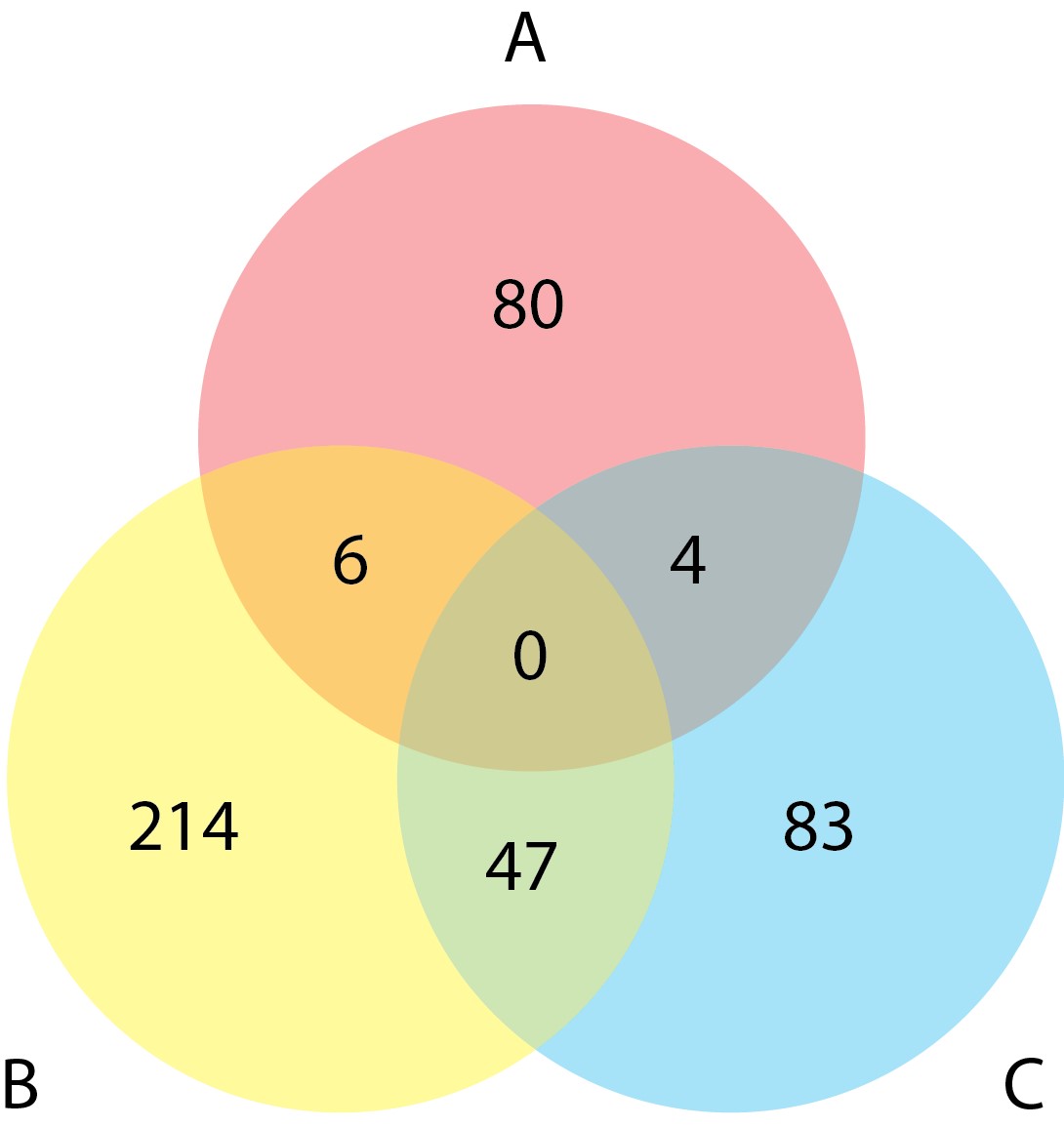

Supplement: Supplementary Figures and Tables [file evx257_supp.zip › Supplementary Figure S6.jpg]
