# Supplementary material for: Plant-Mediated Female Transcriptomic Changes Post-Mating in a Tephritid Fruit Fly, Bactrocera tryoni
Source: Genome Biol Evol. 2017 Dec 6;10(1):94–107. doi: 10.1093/gbe/evx257 (PMC5765559; doi:10.1093/gbe/evx257)
Supplement: Supplementary Figures and Tables [file evx257_supp.zip › Supplementary tables.doc]

Table S1. Gene transcript up-regulated in control-mated females when compared with virgin females

| **ID** | **P Value** | **FDR** | **Functions** | **Reference** |
| --- | --- | --- | --- | --- |
| TRINITY_DN11163_c0_g1 | 4.70E-101 | 3.86E-96 | Unknown | Unknown |
| TRINITY_DN45717_c0_g1 | 6.09E-95 | 2.50E-90 | Attacin | *D. melanogaster* |
| TRINITY_DN42607_c0_g2 | 1.34E-93 | 3.67E-89 | Unknown | Unknown |
| TRINITY_DN35850_c0_g1 | 8.25E-70 | 1.70E-65 | Unknown | Unknown |
| TRINITY_DN30458_c0_g1 | 1.82E-66 | 3.00E-62 | Defensin | *A. mellifera* |
| TRINITY_DN28993_c0_g1 | 4.19E-63 | 5.74E-59 | Unknown | Unknown |
| TRINITY_DN32301_c0_g1 | 5.05E-60 | 5.94E-56 | Diptericin | *D. melanogaster* |
| TRINITY_DN45717_c0_g4 | 2.42E-58 | 2.49E-54 | Unknown | Unknown |
| TRINITY_DN41706_c1_g1 | 7.55E-58 | 6.90E-54 | PGPLB/PGRP – peptidoglycan recognition protein (immune related) | *D. melanogaster* |
| TRINITY_DN37596_c0_g1 | 1.42E-52 | 1.17E-48 | Defensin | *Stomoxys* |
| TRINITY_DN13697_c0_g1 | 2.42E-50 | 1.66E-46 | Unknown | Unknown |
| TRINITY_DN45717_c0_g3 | 2.54E-46 | 1.49E-42 | Attacin | *D. melanogaster* |
| TRINITY_DN34577_c0_g1 | 3.51E-46 | 1.92E-42 | Unknown | Unknown |
| TRINITY_DN57009_c0_g1 | 3.51E-45 | 1.80E-41 | Unknown | Unknown |
| TRINITY_DN21246_c0_g1 | 1.01E-42 | 4.87E-39 | Unknown | Unknown |
| TRINITY_DN30538_c0_g1 | 2.38E-41 | 1.03E-37 | Sapecin | *Sarcophaga* |
| TRINITY_DN35123_c0_g1 | 2.77E-40 | 1.14E-36 | Cecropin | *C. capitata* |
| TRINITY_DN18343_c0_g1 | 8.77E-40 | 3.44E-36 | Unknown | Unknown |
| TRINITY_DN24138_c0_g1 | 3.57E-39 | 1.34E-35 | Unknown | Unknown |
| TRINITY_DN28001_c0_g3 | 8.42E-38 | 3.01E-34 | Unknown | Unknown |
| TRINITY_DN35674_c1_g1 | 1.17E-35 | 3.86E-32 | Unknown | Unknown |
| TRINITY_DN33903_c0_g1 | 5.21E-35 | 1.65E-31 | Sarcotoxin | *Sarcophaga* |
| TRINITY_DN42607_c0_g1 | 7.52E-35 | 2.29E-31 | Unknown | Unknown |
| TRINITY_DN16791_c0_g1 | 1.12E-34 | 3.28E-31 | Unknown | Unknown |
| TRINITY_DN1676_c0_g1 | 4.32E-34 | 1.22E-30 | Unknown | Unknown |
| TRINITY_DN47162_c0_g1 | 1.02E-31 | 2.72E-28 | Unknown | Unknown |
| TRINITY_DN41896_c0_g2 | 2.31E-30 | 5.75E-27 | Unknown | Unknown |
| TRINITY_DN37726_c0_g1 | 3.82E-30 | 9.24E-27 | Unknown | Unknown |
| TRINITY_DN31428_c1_g1 | 2.70E-26 | 5.69E-23 | Unknown | Unknown |
| TRINITY_DN45801_c0_g1 | 3.00E-26 | 6.16E-23 | Ataxin | *B. dorsalis* |
| TRINITY_DN42906_c0_g8 | 1.08E-25 | 2.16E-22 | Unknown | Unknown |
| TRINITY_DN36289_c0_g4 | 3.83E-25 | 7.50E-22 | Unknown | Unknown |
| TRINITY_DN8949_c0_g1 | 1.59E-24 | 2.97E-21 | Unknown | Unknown |
| TRINITY_DN40151_c0_g2 | 1.05E-23 | 1.84E-20 | Unknown | Unknown |
| TRINITY_DN25531_c0_g2 | 1.56E-23 | 2.68E-20 | Unknown | Unknown |
| TRINITY_DN28376_c0_g1 | 5.27E-22 | 8.84E-19 | TC1 Transposable element | *Caenorhabditis* |
| TRINITY_DN44156_c1_g1 | 5.41E-22 | 8.89E-19 | Unknown | Unknown |
| TRINITY_DN41053_c1_g2 | 2.19E-21 | 3.53E-18 | Unknown | Unknown |
| TRINITY_DN41904_c0_g3 | 2.68E-21 | 4.23E-18 | Unknown | Unknown |
| TRINITY_DN46263_c0_g1 | 3.11E-21 | 4.82E-18 | Titin | *D. melanogaster* |
| TRINITY_DN46861_c1_g2 | 3.32E-21 | 5.06E-18 | Unknown | Unknown |
| TRINITY_DN42965_c2_g1 | 3.92E-21 | 5.86E-18 | Attacin | Unknown |
| TRINITY_DN39259_c0_g1 | 6.16E-21 | 9.05E-18 | Unknown | Unknown |
| TRINITY_DN25682_c0_g2 | 1.31E-20 | 1.89E-17 | Unknown | Unknown |
| TRINITY_DN35156_c0_g3 | 2.32E-19 | 3.23E-16 | Unknown | Unknown |
| TRINITY_DN58548_c0_g1 | 2.60E-19 | 3.56E-16 | Unknown | Unknown |
| TRINITY_DN34788_c0_g2 | 4.27E-19 | 5.76E-16 | Unknown | Unknown |
| TRINITY_DN40753_c0_g3 | 1.37E-18 | 1.81E-15 | Unknown | Unknown |
| TRINITY_DN47676_c0_g1 | 1.58E-18 | 2.03E-15 | Unknown | Unknown |
| TRINITY_DN46869_c0_g1 | 2.54E-18 | 3.21E-15 | Titin | *Mus* |
| TRINITY_DN44456_c2_g1 | 2.98E-18 | 3.71E-15 | FBCD1 Fibrinogen D-domain containing protein | *D. melanogaster* |
| TRINITY_DN19930_c0_g2 | 2.22E-17 | 2.73E-14 | Unknown | Unknown |
| TRINITY_DN42725_c1_g3 | 3.54E-17 | 4.28E-14 | Unknown | Unknown |
| TRINITY_DN45882_c4_g1 | 4.78E-17 | 5.70E-14 | Unknown | Unknown |
| TRINITY_DN37892_c0_g1 | 3.49E-16 | 4.04E-13 | Unknown | Unknown |
| TRINITY_DN27922_c0_g1 | 3.84E-16 | 4.39E-13 | Unknown | Unknown |
| TRINITY_DN26091_c0_g1 | 4.41E-16 | 4.96E-13 | Unknown | Unknown |
| TRINITY_DN43773_c0_g1 | 1.15E-15 | 1.27E-12 | Nitric Oxide Synthase | *D. melanogaster* |
| TRINITY_DN46908_c0_g1 | 1.92E-15 | 2.08E-12 | Titin | DN46908 |
| TRINITY_DN40151_c0_g4 | 2.19E-15 | 2.34E-12 | Unknown | Unknown |
| TRINITY_DN46996_c0_g3 | 2.35E-15 | 2.48E-12 | Unknown | Unknown |
| TRINITY_DN37536_c0_g1 | 2.98E-15 | 3.10E-12 | Unknown | Unknown |
| TRINITY_DN35011_c0_g3 | 3.66E-15 | 3.76E-12 | Unknown | Unknown |
| TRINITY_DN45128_c0_g2 | 3.77E-15 | 3.82E-12 | Unknown | Unknown |
| TRINITY_DN2210_c0_g2 | 8.24E-15 | 8.16E-12 | YI31B polyprotein | Saccharomyces |
| TRINITY_DN42496_c1_g1 | 1.44E-14 | 1.39E-11 | Unknown | Unknown |
| TRINITY_DN425_c0_g2 | 1.72E-14 | 1.65E-11 | Unknown | Unknown |
| TRINITY_DN47197_c1_g2 | 2.64E-14 | 2.50E-11 | Nespirin | *B. dorsalis* |
| TRINITY_DN32664_c0_g1 | 4.46E-14 | 4.17E-11 | Unknown | Unknown |
| TRINITY_DN41227_c0_g1 | 4.63E-14 | 4.27E-11 | Unknown | Unknown |
| TRINITY_DN46861_c1_g1 | 5.05E-14 | 4.61E-11 | Unknown | Unknown |
| TRINITY_DN47197_c1_g1 | 6.98E-14 | 6.31E-11 | SYNE1 - Nesprin | *Mus* |
| TRINITY_DN44156_c0_g1 | 7.97E-14 | 7.12E-11 | Unknown | Unknown |
| TRINITY_DN44600_c0_g1 | 1.47E-13 | 1.28E-10 | Titin | *D. melanogaster* |
| TRINITY_DN30449_c0_g1 | 2.08E-13 | 1.80E-10 | Pecanex protein | *D. melanogaster* |
| TRINITY_DN45025_c0_g1 | 4.35E-13 | 3.73E-10 | Unknown | Unknown |
| TRINITY_DN46996_c0_g1 | 5.63E-13 | 4.73E-10 | Unknown | Unknown |
| TRINITY_DN44600_c0_g2 | 6.88E-13 | 5.71E-10 | Titin | *D. melanogaster* |
| TRINITY_DN40180_c0_g2 | 8.11E-13 | 6.60E-10 | Unknown | Unknown |
| TRINITY_DN37965_c0_g1 | 9.11E-13 | 7.34E-10 | Unknown | Unknown |
| TRINITY_DN20441_c0_g1 | 9.41E-13 | 7.51E-10 | Unknown | Unknown |
| TRINITY_DN44749_c0_g2 | 1.25E-12 | 9.85E-10 | Unknown | Unknown |
| TRINITY_DN43758_c0_g1 | 1.30E-12 | 1.02E-09 | Unknown | Unknown |
| TRINITY_DN37004_c0_g1 | 1.34E-12 | 1.04E-09 | EST6 Venm carboxylesterase | *Apis* |
| TRINITY_DN27743_c0_g1 | 1.43E-12 | 1.10E-09 | Unknown | Unknown |
| TRINITY_DN40623_c0_g1 | 1.50E-12 | 1.15E-09 | Unknown | Unknown |
| TRINITY_DN43042_c1_g5 | 1.81E-12 | 1.36E-09 | Cecropin | Unknown |
| TRINITY_DN47200_c0_g1 | 2.24E-12 | 1.67E-09 | Unknown | Unknown |
| TRINITY_DN38649_c1_g1 | 2.29E-12 | 1.70E-09 | Unknown | Unknown |
| TRINITY_DN45697_c1_g1 | 2.88E-12 | 2.08E-09 | Titin | *D. melanogaster* |
| TRINITY_DN47122_c0_g1 | 2.88E-12 | 2.08E-09 | Unknown | Unknown |
| TRINITY_DN38468_c0_g1 | 3.51E-12 | 2.47E-09 | Pecanex protein | *D. melanogaster* |
| TRINITY_DN43042_c1_g3 | 4.19E-12 | 2.92E-09 | Unknown | Unknown |
| TRINITY_DN46224_c0_g1 | 4.83E-12 | 3.34E-09 | Unknown | Unknown |
| TRINITY_DN38331_c0_g1 | 7.43E-12 | 5.05E-09 | CH19, Chorion protein | *D. grimshawi* |
| TRINITY_DN40440_c0_g1 | 7.65E-12 | 5.16E-09 | Unknown | Unknown |
| TRINITY_DN43042_c1_g6 | 9.14E-12 | 6.06E-09 | Unknown | Unknown |
| TRINITY_DN41684_c0_g1 | 1.03E-11 | 6.70E-09 | BGBP3 | *D. melanogaster* |
| TRINITY_DN37449_c0_g1 | 1.47E-11 | 9.51E-09 | Unknown | Unknown |
| TRINITY_DN45882_c1_g1 | 1.51E-11 | 9.67E-09 | Unknown | Unknown |
| TRINITY_DN47020_c0_g1 | 1.65E-11 | 1.05E-08 | Unknown | Unknown |
| TRINITY_DN43690_c0_g1 | 2.42E-11 | 1.53E-08 | Unknown | Unknown |
| TRINITY_DN38010_c0_g1 | 2.51E-11 | 1.58E-08 | Unknown | Unknown |
| TRINITY_DN41998_c0_g1 | 2.59E-11 | 1.60E-08 | Unknown | Unknown |
| TRINITY_DN47233_c0_g1 | 4.01E-11 | 2.42E-08 | Titin | *D. melanogaster* |
| TRINITY_DN43042_c1_g2 | 4.03E-11 | 2.42E-08 | Cecropin | *D. melanogaster* |
| TRINITY_DN44948_c0_g1 | 4.26E-11 | 2.54E-08 | Twitchin | *Caenorhabditis* |
| TRINITY_DN37002_c0_g1 | 4.61E-11 | 2.73E-08 | Protein encore | *D. melanogaster* |
| TRINITY_DN32586_c0_g1 | 7.31E-11 | 4.21E-08 | Pecanex protein | *D. melanogaster* |
| TRINITY_DN45572_c0_g1 | 7.32E-11 | 4.21E-08 | Unknown | Unknown |
| TRINITY_DN25164_c0_g1 | 7.93E-11 | 4.50E-08 | BGBP3 | *Sophophora* |
| TRINITY_DN68178_c0_g1 | 9.03E-11 | 5.08E-08 | Unknown | Unknown |
| TRINITY_DN41533_c0_g1 | 9.75E-11 | 5.45E-08 | Unknown | Unknown |
| TRINITY_DN64841_c0_g1 | 1.08E-10 | 6.00E-08 | Unknown | Unknown |
| TRINITY_DN43042_c1_g8 | 1.20E-10 | 6.64E-08 | Cecropin | Unknown |
| TRINITY_DN28913_c0_g1 | 1.22E-10 | 6.70E-08 | Unknown | Unknown |
| TRINITY_DN32774_c0_g1 | 1.34E-10 | 7.30E-08 | Unknown | Unknown |
| TRINITY_DN34724_c0_g1 | 1.70E-10 | 8.90E-08 | Protein encore | *D. melanogaster* |
| TRINITY_DN37750_c0_g2 | 2.13E-10 | 1.11E-07 | Unknown | Unknown |
| TRINITY_DN44128_c0_g1 | 2.20E-10 | 1.14E-07 | Putative 115kDa protein | *D. melanogaster* |
| TRINITY_DN43682_c0_g1 | 2.58E-10 | 1.32E-07 | HR4 Hormone receptor | *D. melanogaster* |
| TRINITY_DN47133_c1_g1 | 2.71E-10 | 1.38E-07 | Unknown | Unknown |
| TRINITY_DN46012_c0_g1 | 2.81E-10 | 1.42E-07 | TRA1 – H3 acetylation | *D. melanogaster* |
| TRINITY_DN47020_c0_g2 | 2.81E-10 | 1.42E-07 | Replicase polyprotein | Cripavirus |
| TRINITY_DN15826_c0_g1 | 2.94E-10 | 1.48E-07 | Unknown | Unknown |
| TRINITY_DN44950_c0_g1 | 3.22E-10 | 1.60E-07 | Unknown | Unknown |
| TRINITY_DN45168_c2_g1 | 3.34E-10 | 1.65E-07 | Unknown | Unknown |
| TRINITY_DN43212_c0_g1 | 3.67E-10 | 1.81E-07 | Pikachurin - Peptide cross linking | *Mus* |
| TRINITY_DN21782_c0_g1 | 4.29E-10 | 2.10E-07 | Unknown | Unknown |
| TRINITY_DN30097_c0_g1 | 4.49E-10 | 2.18E-07 | Unknown | Unknown |
| TRINITY_DN44729_c0_g2 | 4.90E-10 | 2.37E-07 | Pecanex protein | *C. capitata* |
| TRINITY_DN46843_c0_g3 | 5.00E-10 | 2.41E-07 | Zinc finger protein | *H. sapiens* |
| TRINITY_DN1401_c0_g1 | 5.69E-10 | 2.71E-07 | Apobec complementation factor | *Pongo* |
| TRINITY_DN57818_c0_g1 | 5.70E-10 | 2.71E-07 | Unknown | Unknown |
| TRINITY_DN41053_c1_g1 | 6.65E-10 | 3.14E-07 | Titin | *D. melanogaster* |
| TRINITY_DN43148_c0_g1 | 6.75E-10 | 3.16E-07 | Unknown | Unknown |
| TRINITY_DN46700_c0_g1 | 8.39E-10 | 3.90E-07 | Unknown | Unknown |
| TRINITY_DN68166_c0_g1 | 9.07E-10 | 4.19E-07 | Unknown | Unknown |
| TRINITY_DN39469_c0_g1 | 9.33E-10 | 4.29E-07 | Unknown | Unknown |
| TRINITY_DN43468_c0_g1 | 9.90E-10 | 4.52E-07 | Unknown | Unknown |
| TRINITY_DN3575_c0_g1 | 1.02E-09 | 4.63E-07 | Unknown | Unknown |
| TRINITY_DN41898_c0_g1 | 1.09E-09 | 4.94E-07 | Unknown | Unknown |
| TRINITY_DN28933_c0_g1 | 1.63E-09 | 7.27E-07 | Protein encore | *C. capitata* |
| TRINITY_DN47243_c1_g2 | 1.86E-09 | 8.24E-07 | Unknown | Unknown |
| TRINITY_DN43795_c1_g1 | 2.03E-09 | 8.93E-07 | Unknown | Unknown |
| TRINITY_DN47243_c0_g2 | 2.15E-09 | 9.42E-07 | Unknown | Unknown |
| TRINITY_DN31826_c0_g1 | 2.98E-09 | 1.29E-06 | Unknown | Unknown |
| TRINITY_DN46996_c0_g2 | 3.12E-09 | 1.34E-06 | Unknown | Unknown |
| TRINITY_DN40534_c0_g1 | 3.15E-09 | 1.34E-06 | UDB31 | *B. dorsalis* |
| TRINITY_DN45756_c0_g2 | 3.20E-09 | 1.36E-06 | Unknown | Unknown |
| TRINITY_DN36567_c0_g1 | 4.54E-09 | 1.90E-06 | Unknown | Unknown |
| TRINITY_DN35047_c0_g1 | 4.60E-09 | 1.91E-06 | Unknown | Unknown |
| TRINITY_DN43042_c1_g7 | 4.61E-09 | 1.91E-06 | Cecropin | Unknown |
| TRINITY_DN13156_c0_g2 | 4.72E-09 | 1.94E-06 | Unknown | Unknown |
| TRINITY_DN15798_c0_g1 | 5.37E-09 | 2.20E-06 | Unknown | Unknown |
| TRINITY_DN63866_c0_g1 | 6.08E-09 | 2.47E-06 | Unknown | Unknown |
| TRINITY_DN44372_c0_g2 | 6.15E-09 | 2.49E-06 | Unknown | Unknown |
| TRINITY_DN42496_c2_g1 | 6.47E-09 | 2.61E-06 | Unknown | Unknown |
| TRINITY_DN38768_c0_g1 | 6.64E-09 | 2.66E-06 | Unknown | Unknown |
| TRINITY_DN30173_c0_g1 | 6.84E-09 | 2.72E-06 | Unknown | Unknown |
| TRINITY_DN40539_c0_g1 | 8.32E-09 | 3.26E-06 | Zinc finger protein | *C. capitata* |
| TRINITY_DN39279_c0_g1 | 9.02E-09 | 3.48E-06 | Tramtract protein | *C. capitata* |
| TRINITY_DN32562_c0_g1 | 9.87E-09 | 3.79E-06 | Gag polyprotein | *C. capitata* |
| TRINITY_DN5668_c0_g1 | 1.06E-08 | 4.04E-06 | Unknown | Unknown |
| TRINITY_DN32788_c0_g1 | 1.08E-08 | 4.13E-06 | Unknown | Unknown |
| TRINITY_DN43503_c0_g1 | 1.26E-08 | 4.76E-06 | Unknown | Unknown |
| TRINITY_DN15276_c0_g2 | 1.33E-08 | 5.00E-06 | Unknown | Unknown |
| TRINITY_DN43351_c0_g1 | 1.35E-08 | 5.05E-06 | Unknown | Unknown |
| TRINITY_DN46289_c2_g2 | 1.41E-08 | 5.28E-06 | Unknown | Unknown |
| TRINITY_DN45952_c0_g4 | 1.45E-08 | 5.39E-06 | Unknown | Unknown |
| TRINITY_DN43542_c0_g1 | 1.62E-08 | 5.98E-06 | Unknown | Unknown |
| TRINITY_DN40558_c0_g1 | 1.68E-08 | 6.21E-06 | Unknown | Unknown |
| TRINITY_DN41169_c0_g1 | 1.93E-08 | 7.05E-06 | Unknown | Unknown |
| TRINITY_DN45697_c0_g1 | 2.09E-08 | 7.55E-06 | Unknown | Unknown |
| TRINITY_DN36449_c0_g1 | 2.28E-08 | 8.08E-06 | Unknown | Unknown |
| TRINITY_DN26024_c0_g1 | 2.80E-08 | 9.79E-06 | Titin | *D. melanogaste; B. dorsalis* |
| TRINITY_DN36941_c0_g1 | 2.81E-08 | 9.79E-06 | Unknown | Unknown |
| TRINITY_DN34345_c0_g2 | 2.89E-08 | 1.00E-05 | Unknown | Unknown |
| TRINITY_DN47243_c1_g1 | 3.01E-08 | 1.04E-05 | Muscle M-line protein | *B. dorsalis* |
| TRINITY_DN36962_c0_g1 | 3.03E-08 | 1.04E-05 | Glucuronosyltransferase | *B. dorsalis* |
| TRINITY_DN43594_c10_g3 | 3.79E-08 | 1.29E-05 | Unknown | Unknown |
| TRINITY_DN39098_c0_g1 | 3.99E-08 | 1.34E-05 | Unknown | Unknown |
| TRINITY_DN42716_c0_g1 | 4.38E-08 | 1.47E-05 | Unknown | Unknown |
| TRINITY_DN36686_c0_g2 | 4.50E-08 | 1.50E-05 | Polyprotein | *D. melanogaster* |
| TRINITY_DN47258_c4_g1 | 4.74E-08 | 1.58E-05 | Unknown | Unknown |
| TRINITY_DN33359_c0_g1 | 4.97E-08 | 1.65E-05 | Unknown | Unknown |
| TRINITY_DN32259_c0_g1 | 5.00E-08 | 1.65E-05 | Zinc finger protein | *B. dorsalis* |
| TRINITY_DN44711_c0_g6 | 5.03E-08 | 1.66E-05 | Unknown | Unknown |
| TRINITY_DN37623_c0_g1 | 5.27E-08 | 1.73E-05 | Unknown | Unknown |
| TRINITY_DN39130_c0_g1 | 5.59E-08 | 1.82E-05 | Unknown | Unknown |
| TRINITY_DN36326_c0_g2 | 5.75E-08 | 1.87E-05 | Unknown | Unknown |
| TRINITY_DN18819_c0_g2 | 5.99E-08 | 1.94E-05 | Unknown | Unknown |
| TRINITY_DN41370_c0_g1 | 6.20E-08 | 2.00E-05 | Unknown | Unknown |
| TRINITY_DN2074_c0_g1 | 6.23E-08 | 2.00E-05 | Piggybac transposable element (PGBD4) | *H. sapiens* |
| TRINITY_DN24288_c0_g2 | 6.31E-08 | 2.02E-05 | Unknown | Unknown |
| TRINITY_DN39379_c0_g1 | 6.45E-08 | 2.06E-05 | Unknown | Unknown |
| TRINITY_DN36980_c0_g1 | 7.29E-08 | 2.31E-05 | Unknown | Unknown |
| TRINITY_DN31924_c0_g1 | 7.48E-08 | 2.37E-05 | Unknown | Unknown |
| TRINITY_DN1493_c0_g1 | 8.85E-08 | 2.77E-05 | Unknown | Unknown |
| TRINITY_DN39658_c0_g1 | 9.79E-08 | 3.05E-05 | Unknown | Unknown |
| TRINITY_DN34598_c0_g1 | 9.95E-08 | 3.09E-05 | Unknown | Unknown |
| TRINITY_DN46319_c2_g2 | 1.03E-07 | 3.18E-05 | Unknown | Unknown |
| TRINITY_DN46557_c0_g1 | 1.08E-07 | 3.32E-05 | Unknown | Unknown |
| TRINITY_DN42114_c2_g1 | 1.12E-07 | 3.43E-05 | Unknown | Unknown |
| TRINITY_DN36185_c0_g1 | 1.13E-07 | 3.47E-05 | Unknown | Unknown |
| TRINITY_DN28218_c0_g1 | 1.19E-07 | 3.60E-05 | Unknown | Unknown |
| TRINITY_DN28218_c0_g2 | 1.22E-07 | 3.69E-05 | Unknown | Unknown |
| TRINITY_DN27705_c0_g1 | 1.25E-07 | 3.76E-05 | Unknown | Unknown |
| TRINITY_DN39630_c0_g1 | 1.26E-07 | 3.77E-05 | Unknown | Unknown |
| TRINITY_DN38077_c0_g1 | 1.38E-07 | 4.09E-05 | Unknown | Unknown |
| TRINITY_DN34229_c0_g1 | 1.38E-07 | 4.09E-05 | Unknown | Unknown |
| TRINITY_DN69188_c0_g1 | 1.46E-07 | 4.29E-05 | Unknown | Unknown |
| TRINITY_DN41020_c2_g3 | 1.58E-07 | 4.63E-05 | Unknown | Unknown |
| TRINITY_DN5917_c0_g1 | 1.62E-07 | 4.72E-05 | Unknown | Unknown |
| TRINITY_DN46544_c0_g1 | 1.78E-07 | 5.16E-05 | Unknown | Unknown |
| TRINITY_DN42496_c2_g2 | 1.89E-07 | 5.47E-05 | Unknown | Unknown |
| TRINITY_DN6402_c0_g2 | 1.99E-07 | 5.73E-05 | Unknown | Unknown |
| TRINITY_DN23905_c0_g1 | 2.20E-07 | 6.27E-05 | Unknown | Unknown |
| TRINITY_DN46639_c0_g1 | 2.39E-07 | 6.77E-05 | Unknown | Unknown |
| TRINITY_DN19307_c0_g2 | 2.43E-07 | 6.88E-05 | Unknown | Unknown |
| TRINITY_DN32061_c0_g2 | 2.86E-07 | 8.06E-05 | Unknown | Unknown |
| TRINITY_DN45922_c0_g2 | 2.94E-07 | 8.23E-05 | Unknown | Unknown |
| TRINITY_DN31261_c0_g2 | 3.02E-07 | 8.42E-05 | Unknown | Unknown |
| TRINITY_DN50104_c0_g1 | 3.12E-07 | 8.67E-05 | Pecanex protein | *D. melanogaster* |
| TRINITY_DN47243_c0_g1 | 3.19E-07 | 8.84E-05 | Titin | *B. dorsalis* |
| TRINITY_DN44156_c1_g2 | 3.21E-07 | 8.85E-05 | Unknown | Unknown |
| TRINITY_DN46777_c0_g6 | 3.41E-07 | 9.37E-05 | Unknown | Unknown |
| TRINITY_DN44016_c0_g1 | 3.53E-07 | 9.63E-05 | Unknown | Unknown |
| TRINITY_DN32061_c0_g1 | 3.74E-07 | 0.000102 | Unknown | Unknown |
| TRINITY_DN45128_c0_g1 | 3.82E-07 | 0.000103 | Unknown | Unknown |
| TRINITY_DN45658_c0_g2 | 3.82E-07 | 0.000103 | TIS11 | *D. melanogaster* |
| TRINITY_DN42913_c0_g2 | 4.04E-07 | 0.000109 | Unknown | Unknown |
| TRINITY_DN37488_c0_g1 | 4.32E-07 | 0.000116 | Unknown | Unknown |
| TRINITY_DN38265_c0_g1 | 4.36E-07 | 0.000116 | Unknown | Unknown |
| TRINITY_DN42716_c0_g2 | 4.50E-07 | 0.00012 | Unknown | Unknown |
| TRINITY_DN17914_c0_g1 | 4.59E-07 | 0.000122 | Unknown | Unknown |
| TRINITY_DN5956_c0_g1 | 4.73E-07 | 0.000125 | Polyprotein | Unknown |
| TRINITY_DN6604_c0_g1 | 4.76E-07 | 0.000126 | Unknown | Unknown |
| TRINITY_DN44480_c0_g3 | 4.82E-07 | 0.000126 | Unknown | Unknown |
| TRINITY_DN37623_c0_g3 | 5.02E-07 | 0.00013 | Unknown | Unknown |
| TRINITY_DN19603_c0_g1 | 5.19E-07 | 0.000134 | Unknown | Unknown |
| TRINITY_DN40661_c0_g2 | 5.38E-07 | 0.000139 | Unknown | Unknown |
| TRINITY_DN44566_c0_g1 | 5.46E-07 | 0.00014 | Protein methionine sulfoxide oxidase | *D. melanogaster* |
| TRINITY_DN7415_c0_g1 | 5.84E-07 | 0.000149 | Unknown | Unknown |
| TRINITY_DN45192_c0_g17 | 5.98E-07 | 0.000153 | Unknown | Unknown |
| TRINITY_DN45453_c1_g5 | 6.10E-07 | 0.000155 | Retrotransposable element | *D. melanogaster* |
| TRINITY_DN42969_c0_g2 | 6.50E-07 | 0.000165 | Unknown | Unknown |
| TRINITY_DN29185_c0_g2 | 6.55E-07 | 0.000166 | Unknown | Unknown |
| TRINITY_DN38273_c0_g1 | 6.69E-07 | 0.000168 | Unknown | Unknown |
| TRINITY_DN39570_c0_g1 | 6.69E-07 | 0.000168 | Unknown | Unknown |
| TRINITY_DN38436_c0_g1 | 6.77E-07 | 0.00017 | TCB1 Transposable element | *Caenorhabditis* |
| TRINITY_DN27080_c0_g1 | 6.83E-07 | 0.000171 | Unknown | Unknown |
| TRINITY_DN42400_c0_g2 | 6.90E-07 | 0.000172 | Unknown | Unknown |

Table S2. Gene transcripts down-regulated in control-mated females when compared with virgin females

| **ID** | **P Value** | **FDR** | **Functions** | **Reference** |
| --- | --- | --- | --- | --- |
| TRINITY_DN37216_c0_g1 | 5.56E-52 | 4.16E-48 | CUA1 Cuticular protein | *D. melanogaster* |
| TRINITY_DN18302_c0_g2 | 8.06E-50 | 5.10E-46 | CUA1 Cuticular protein | *D. melanogaster* |
| TRINITY_DN39591_c0_g1 | 1.08E-41 | 4.92E-38 | CUCA1 Cuticular protein | *D. melanogaster* |
| TRINITY_DN8949_c0_g4 | 1.67E-37 | 5.71E-34 | unknown | unknown |
| TRINITY_DN21290_c0_g4 | 2.44E-33 | 6.69E-30 | unknown | unknown |
| TRINITY_DN43019_c1_g1 | 1.85E-31 | 4.75E-28 | unknown | unknown |
| TRINITY_DN40205_c0_g1 | 5.25E-30 | 1.23E-26 | unknown | unknown |
| TRINITY_DN18302_c0_g1 | 1.19E-27 | 2.71E-24 | unknown | unknown |
| TRINITY_DN30500_c0_g1 | 1.41E-27 | 3.12E-24 | CUA1 Cuticular protein | *D. melanogaster* |
| TRINITY_DN43052_c0_g1 | 1.91E-27 | 4.13E-24 | unknown | unknown |
| TRINITY_DN8487_c0_g2 | 4.37E-25 | 8.35E-22 | unknown | unknown |
| TRINITY_DN33947_c0_g1 | 2.42E-24 | 4.42E-21 | CU01 Cuticular protein | *Blaberus* |
| TRINITY_DN21589_c0_g1 | 1.02E-23 | 1.82E-20 | W8AND4 polyprotein | *Hepatovirus* |
| TRINITY_DN19931_c0_g1 | 4.11E-20 | 5.82E-17 | unknown | unknown |
| TRINITY_DN4240_c0_g1 | 1.39E-18 | 1.81E-15 | unknown | unknown |
| TRINITY_DN33682_c0_g2 | 3.43E-16 | 4.03E-13 | unknown | unknown |
| TRINITY_DN18122_c0_g2 | 1.21E-15 | 1.32E-12 | unknown | unknown |
| TRINITY_DN21650_c0_g1 | 4.79E-15 | 4.80E-12 | unknown | unknown |
| TRINITY_DN33285_c0_g1 | 1.37E-14 | 1.34E-11 | unknown | unknown |
| TRINITY_DN39893_c0_g1 | 1.33E-13 | 1.18E-10 | Polymerase acidic protein | *C. capitata* |
| TRINITY_DN24536_c0_g1 | 5.63E-13 | 4.73E-10 | unknown | unknown |
| TRINITY_DN26618_c0_g3 | 7.18E-13 | 5.90E-10 | unknown | unknown |
| TRINITY_DN37815_c0_g2 | 2.31E-12 | 1.70E-09 | unknown | unknown |
| TRINITY_DN1401_c0_g2 | 3.16E-12 | 2.26E-09 | unknown | unknown |
| TRINITY_DN39100_c0_g2 | 3.22E-12 | 2.29E-09 | unknown | unknown |
| TRINITY_DN39931_c0_g2 | 6.50E-12 | 4.45E-09 | unknown | unknown |
| TRINITY_DN39522_c0_g1 | 7.79E-12 | 5.21E-09 | unknown | unknown |
| TRINITY_DN26618_c0_g2 | 1.01E-11 | 6.65E-09 | unknown | unknown |
| TRINITY_DN29822_c0_g1 | 2.56E-11 | 1.59E-08 | unknown | unknown |
| TRINITY_DN39841_c0_g1 | 3.36E-11 | 2.06E-08 | unknown | unknown |
| TRINITY_DN41455_c0_g2 | 3.66E-11 | 2.23E-08 | unknown | unknown |
| TRINITY_DN46485_c0_g1 | 7.00E-11 | 4.09E-08 | unknown | unknown |
| TRINITY_DN7797_c0_g2 | 7.01E-11 | 4.09E-08 | unknown | unknown |
| TRINITY_DN26618_c0_g1 | 7.62E-11 | 4.35E-08 | unknown | unknown |
| TRINITY_DN34586_c0_g1 | 1.63E-10 | 8.82E-08 | unknown | unknown |
| TRINITY_DN23077_c0_g1 | 1.65E-10 | 8.85E-08 | unknown | unknown |
| TRINITY_DN27572_c0_g1 | 1.66E-10 | 8.85E-08 | unknown | unknown |
| TRINITY_DN24767_c0_g1 | 1.67E-10 | 8.85E-08 | unknown | unknown |
| TRINITY_DN34103_c0_g2 | 1.70E-10 | 8.90E-08 | unknown | unknown |
| TRINITY_DN52293_c0_g1 | 6.71E-10 | 3.15E-07 | unknown | unknown |
| TRINITY_DN40836_c0_g1 | 1.15E-09 | 5.18E-07 | CUP7 Pupal cuticular protein | *D. melanogaster* |
| TRINITY_DN34299_c0_g1 | 1.73E-09 | 7.67E-07 | unknown | unknown |
| TRINITY_DN41771_c0_g1 | 2.26E-09 | 9.83E-07 | unknown | unknown |
| TRINITY_DN42328_c0_g1 | 3.12E-09 | 1.34E-06 | unknown | unknown |
| TRINITY_DN42686_c0_g1 | 3.36E-09 | 1.42E-06 | unknown | unknown |
| TRINITY_DN23750_c0_g1 | 3.77E-09 | 1.58E-06 | unknown | unknown |
| TRINITY_DN35108_c0_g2 | 6.67E-09 | 2.66E-06 | unknown | unknown |
| TRINITY_DN45904_c0_g6 | 7.49E-09 | 2.96E-06 | unknown | unknown |
| TRINITY_DN42053_c0_g1 | 7.73E-09 | 3.04E-06 | unknown | unknown |
| TRINITY_DN9831_c0_g2 | 8.88E-09 | 3.46E-06 | GAGHB Gag polyprotein | *D. melanogaster* |
| TRINITY_DN31500_c0_g1 | 9.02E-09 | 3.48E-06 | unknown | unknown |
| TRINITY_DN61836_c0_g1 | 1.92E-08 | 7.03E-06 | unknown | unknown |
| TRINITY_DN38482_c0_g1 | 2.06E-08 | 7.49E-06 | Lithostathine - Myelination | *Mus* |
| TRINITY_DN18063_c0_g1 | 2.11E-08 | 7.60E-06 | unknown | unknown |
| TRINITY_DN39522_c0_g5 | 2.16E-08 | 7.76E-06 | unknown | unknown |
| TRINITY_DN41472_c0_g1 | 2.25E-08 | 8.05E-06 | unknown | unknown |
| TRINITY_DN15878_c0_g1 | 2.26E-08 | 8.05E-06 | unknown | unknown |
| TRINITY_DN43432_c0_g2 | 2.35E-08 | 8.28E-06 | unknown | unknown |
| TRINITY_DN10519_c0_g1 | 2.43E-08 | 8.53E-06 | TIGD6 Transposable element | *B. dorsalis* |
| TRINITY_DN42650_c0_g1 | 2.96E-08 | 1.02E-05 | unknown | unknown |
| TRINITY_DN40577_c0_g1 | 3.44E-08 | 1.17E-05 | unknown | unknown |
| TRINITY_DN16762_c0_g2 | 3.90E-08 | 1.32E-05 | unknown | unknown |
| TRINITY_DN17803_c0_g2 | 7.99E-08 | 2.51E-05 | unknown | unknown |
| TRINITY_DN17803_c0_g1 | 7.99E-08 | 2.51E-05 | unknown | unknown |
| TRINITY_DN37091_c0_g1 | 1.14E-07 | 3.47E-05 | unknown | unknown |
| TRINITY_DN43334_c1_g1 | 1.17E-07 | 3.56E-05 | unknown | unknown |
| TRINITY_DN12954_c0_g1 | 1.30E-07 | 3.87E-05 | unknown | unknown |
| TRINITY_DN43361_c0_g1 | 1.39E-07 | 4.11E-05 | unknown | unknown |
| TRINITY_DN28650_c0_g1 | 1.91E-07 | 5.52E-05 | unknown | unknown |
| TRINITY_DN37187_c0_g1 | 2.00E-07 | 5.73E-05 | GR39B, Gustatory receptor | *D. melanogaster* |
| TRINITY_DN33376_c0_g1 | 2.22E-07 | 6.33E-05 | unknown | unknown |
| TRINITY_DN39648_c0_g2 | 2.89E-07 | 8.11E-05 | unknown | unknown |
| TRINITY_DN30267_c0_g2 | 3.48E-07 | 9.53E-05 | unknown | unknown |
| TRINITY_DN6076_c0_g1 | 3.72E-07 | 0.000101 | unknown | unknown |
| TRINITY_DN9446_c0_g1 | 4.80E-07 | 0.000126 | unknown | unknown |
| TRINITY_DN39522_c0_g4 | 4.93E-07 | 0.000129 | unknown | unknown |
| TRINITY_DN37772_c0_g1 | 5.03E-07 | 0.00013 | unknown | unknown |
| TRINITY_DN41883_c0_g5 | 7.01E-07 | 0.000174 | unknown | unknown |

Table S3. Biological process GO term upregulated in control-mated females when compared with virgin females

| **GO Id** | **GO Function** | **Total numbers in category** | **Number differentially expressed** | **P value** |
| --- | --- | --- | --- | --- |
| GO:0007062 | sister chromatid cohesion | 35 | 10 | 0 |
| GO:0007076 | mitotic chromosome condensation | 37 | 10 | 0 |
| GO:0007522 | visceral muscle development | 19 | 10 | 0 |
| GO:0016203 | muscle attachment | 37 | 10 | 0 |
| GO:0042742 | defense response to bacterium | 184 | 19 | 2.97E-17 |
| GO:0009617 | response to bacterium | 200 | 19 | 1.26E-16 |
| GO:0045087 | innate immune response | 257 | 21 | 7.23E-16 |
| GO:0045214 | sarcomere organization | 60 | 14 | 5.93E-15 |
| GO:0098542 | defense response to other organism | 241 | 19 | 1.08E-14 |
| GO:0051707 | response to other organism | 342 | 21 | 3.47E-14 |
| GO:0006955 | immune response | 354 | 21 | 1.96E-13 |
| GO:0043207 | response to external biotic stimulus | 390 | 21 | 3.71E-13 |
| GO:0009607 | response to biotic stimulus | 393 | 21 | 4.19E-13 |
| GO:0031032 | actomyosin structure organization | 101 | 14 | 7.83E-13 |
| GO:0051704 | multi-organism process | 763 | 27 | 1.73E-12 |
| GO:0007525 | somatic muscle development | 117 | 13 | 2.22E-11 |
| GO:0006952 | defense response | 435 | 21 | 2.81E-11 |
| GO:0002376 | immune system process | 481 | 21 | 6.03E-11 |
| GO:0007519 | skeletal muscle tissue development | 54 | 10 | 1.95E-10 |
| GO:0007520 | myoblast fusion | 56 | 10 | 2.01E-10 |
| GO:0035206 | regulation of hemocyte proliferation | 54 | 10 | 2.8E-10 |
| GO:0030261 | chromosome condensation | 54 | 10 | 3.09E-10 |
| GO:0006323 | DNA packaging | 55 | 10 | 3.48E-10 |
| GO:0000768 | syncytium formation by plasma membrane fusion | 65 | 10 | 6.95E-10 |
| GO:0006949 | syncytium formation | 66 | 10 | 7.67E-10 |
| GO:0014706 | striated muscle tissue development | 66 | 10 | 1.41E-09 |
| GO:0030036 | actin cytoskeleton organization | 227 | 17 | 1.84E-09 |
| GO:0060537 | muscle tissue development | 74 | 10 | 3.31E-09 |
| GO:0030029 | actin filament-based process | 253 | 17 | 4.69E-09 |
| GO:0007517 | muscle organ development | 134 | 11 | 2.1E-08 |
| GO:0007498 | mesoderm development | 94 | 10 | 2.46E-08 |
| GO:0019731 | antibacterial humoral response | 16 | 5 | 2.49E-08 |
| GO:0061061 | muscle structure development | 225 | 13 | 2.9E-08 |
| GO:0007010 | cytoskeleton organization | 597 | 22 | 3.63E-08 |
| GO:0009605 | response to external stimulus | 795 | 22 | 8.15E-08 |
| GO:0071103 | DNA conformation change | 114 | 10 | 1.1E-07 |
| GO:0051276 | chromosome organization | 258 | 13 | 2.14E-07 |
| GO:0050830 | defense response to Gram-positive bacterium | 59 | 6 | 1.3E-06 |
| GO:0007029 | endoplasmic reticulum organization | 31 | 5 | 1.38E-06 |
| GO:0048646 | anatomical structure formation involved in morphogenesis | 685 | 20 | 2.18E-06 |
| GO:0051301 | cell division | 332 | 13 | 7.09E-06 |
| GO:0006996 | organelle organization | 1805 | 31 | 3.48E-05 |
| GO:0048036 | central complex development | 11 | 3 | 5.81E-05 |
| GO:0040011 | Locomotion | 780 | 17 | 6.61E-05 |
| GO:0007399 | nervous system development | 186 | 8 | 6.87E-05 |
| GO:0034114 | regulation of heterotypic cell-cell adhesion | 11 | 3 | 8.34E-05 |
| GO:0034116 | positive regulation of heterotypic cell-cell adhesion | 11 | 3 | 8.34E-05 |
| GO:0002238 | response to molecule of fungal origin | 3 | 2 | 9.98E-05 |
| GO:0032491 | detection of molecule of fungal origin | 3 | 2 | 9.98E-05 |
| GO:0019730 | antimicrobial humoral response | 71 | 5 | 0.000125 |
| GO:1902589 | single-organism organelle organization | 1348 | 25 | 0.000147 |
| GO:0048499 | synaptic vesicle membrane organization | 14 | 3 | 0.000151 |
| GO:0002682 | regulation of immune system process | 431 | 12 | 0.000202 |
| GO:0016200 | synaptic target attraction | 19 | 3 | 0.000287 |
| GO:0050808 | synapse organization | 254 | 8 | 0.000299 |
| GO:0006959 | humoral immune response | 90 | 5 | 0.000338 |
| GO:0048790 | maintenance of presynaptic active zone structure | 15 | 3 | 0.000375 |
| GO:1903047 | mitotic cell cycle process | 627 | 14 | 0.000451 |
| GO:0007317 | regulation of pole plasm oskar mRNA localization | 24 | 3 | 0.000627 |
| GO:0060281 | regulation of oocyte development | 24 | 3 | 0.000627 |
| GO:0007219 | Notch signaling pathway | 105 | 5 | 0.000627 |
| GO:0006950 | response to stress | 1510 | 23 | 0.000706 |
| GO:0050918 | positive chemotaxis | 25 | 3 | 0.000756 |
| GO:0050793 | regulation of developmental process | 1298 | 20 | 0.000779 |
| GO:0007416 | synapse assembly | 160 | 6 | 0.00082 |
| GO:0071688 | striated muscle myosin thick filament assembly | 21 | 5 | 0.000952 |
| GO:0007528 | neuromuscular junction development | 119 | 5 | 0.001088 |
| GO:0030717 | karyosome formation | 25 | 3 | 0.001151 |
| GO:0048856 | anatomical structure development | 2123 | 29 | 0.001242 |
| GO:0019417 | sulfur oxidation | 3 | 2 | 0.001285 |
| GO:0030047 | actin modification | 3 | 2 | 0.001285 |
| GO:0060386 | synapse assembly involved in innervation | 3 | 2 | 0.001285 |
| GO:0031033 | myosin filament organization | 24 | 5 | 0.001328 |
| GO:0031034 | myosin filament assembly | 24 | 5 | 0.001328 |
| GO:2000331 | regulation of terminal button organization | 24 | 3 | 0.00151 |
| GO:0009595 | detection of biotic stimulus | 10 | 2 | 0.001516 |
| GO:0098581 | detection of external biotic stimulus | 10 | 2 | 0.001516 |
| GO:0048513 | organ development | 1015 | 17 | 0.001619 |
| GO:0042127 | regulation of cell proliferation | 505 | 11 | 0.001621 |
| GO:0045785 | positive regulation of cell adhesion | 79 | 4 | 0.001947 |
| GO:0046331 | lateral inhibition | 135 | 5 | 0.002233 |
| GO:0022409 | positive regulation of cell-cell adhesion | 39 | 3 | 0.002265 |
| GO:0016043 | cellular component organization | 3216 | 38 | 0.002296 |
| GO:0051239 | regulation of multicellular organismal process | 1323 | 19 | 0.002333 |
| GO:1902875 | regulation of embryonic pattern specification | 41 | 3 | 0.002357 |
| GO:0045168 | cell-cell signaling involved in cell fate commitment | 137 | 5 | 0.002416 |
| GO:0034110 | regulation of homotypic cell-cell adhesion | 40 | 3 | 0.002495 |
| GO:0070995 | NADPH oxidation | 6 | 2 | 0.002513 |
| GO:0030042 | actin filament depolymerization | 6 | 2 | 0.002694 |
| GO:0071840 | cellular component organization or biogenesis | 3250 | 38 | 0.002703 |
| GO:0001941 | postsynaptic membrane organization | 43 | 3 | 0.002749 |
| GO:0060052 | neurofilament cytoskeleton organization | 15 | 2 | 0.002782 |
| GO:0048134 | germ-line cyst formation | 18 | 3 | 0.003028 |
| GO:0007294 | germarium-derived oocyte fate determination | 19 | 3 | 0.003341 |
| GO:0030716 | oocyte fate determination | 21 | 3 | 0.003783 |
| GO:0009888 | tissue development | 524 | 11 | 0.004094 |
| GO:0008039 | synaptic target recognition | 48 | 3 | 0.004731 |
| GO:0097090 | presynaptic membrane organization | 49 | 3 | 0.004884 |
| GO:0032502 | developmental process | 3787 | 41 | 0.005186 |
| GO:0007282 | cystoblast division | 24 | 3 | 0.005198 |
| GO:0022402 | cell cycle process | 800 | 14 | 0.00595 |
| GO:0031339 | negative regulation of vesicle fusion | 1 | 1 | 0.006063 |
| GO:2000241 | regulation of reproductive process | 58 | 3 | 0.006862 |
| GO:0030241 | skeletal muscle myosin thick filament assembly | 19 | 4 | 0.006892 |
| GO:0042692 | muscle cell differentiation | 38 | 3 | 0.006988 |
| GO:0043954 | cellular component maintenance | 50 | 3 | 0.00719 |
| GO:0051124 | synaptic growth at neuromuscular junction | 56 | 3 | 0.007392 |
| GO:0016032 | viral process | 173 | 5 | 0.007771 |
| GO:0044403 | symbiosis, encompassing mutualism through parasitism | 174 | 5 | 0.007923 |
| GO:0006739 | NADP metabolic process | 15 | 2 | 0.008024 |
| GO:0044764 | multi-organism cellular process | 177 | 5 | 0.008447 |
| GO:0051261 | protein depolymerization | 14 | 2 | 0.008686 |
| GO:0007267 | cell-cell signaling | 434 | 8 | 0.008908 |
| GO:0050807 | regulation of synapse organization | 174 | 5 | 0.010112 |
| GO:0044700 | single organism signaling | 442 | 8 | 0.010215 |
| GO:0023052 | Signaling | 443 | 8 | 0.010358 |
| GO:0045995 | regulation of embryonic development | 111 | 4 | 0.010687 |
| GO:0061158 | 3'-UTR-mediated mRNA destabilization | 2 | 1 | 0.011629 |
| GO:0044419 | interspecies interaction between organisms | 192 | 5 | 0.01177 |
| GO:0048731 | system development | 506 | 9 | 0.012658 |
| GO:0045955 | negative regulation of calcium ion-dependent exocytosis | 2 | 1 | 0.012824 |
| GO:0043578 | nuclear matrix organization | 6 | 2 | 0.013217 |
| GO:0090286 | cytoskeletal anchoring at nuclear membrane | 6 | 2 | 0.013217 |
| GO:0090292 | nuclear matrix anchoring at nuclear membrane | 6 | 2 | 0.013217 |
| GO:0006259 | DNA metabolic process | 1506 | 17 | 0.01334 |
| GO:0002221 | pattern recognition receptor signaling pathway | 28 | 2 | 0.013631 |
| GO:0022407 | regulation of cell-cell adhesion | 74 | 3 | 0.013693 |
| GO:0016322 | neuron remodeling | 22 | 2 | 0.01445 |
| GO:0007293 | germarium-derived egg chamber formation | 51 | 3 | 0.014983 |
| GO:0002758 | innate immune response-activating signal transduction | 30 | 2 | 0.015818 |
| GO:0042551 | neuron maturation | 24 | 2 | 0.016261 |
| GO:0051457 | maintenance of protein location in nucleus | 8 | 2 | 0.017005 |
| GO:0007274 | neuromuscular synaptic transmission | 69 | 3 | 0.017146 |
| GO:0075732 | viral penetration into host nucleus | 37 | 2 | 0.018846 |
| GO:1902594 | multi-organism nuclear import | 37 | 2 | 0.018846 |
| GO:0046496 | nicotinamide nucleotide metabolic process | 28 | 2 | 0.019467 |
| GO:0045103 | intermediate filament-based process | 17 | 2 | 0.019701 |
| GO:0045104 | intermediate filament cytoskeleton organization | 17 | 2 | 0.019701 |
| GO:0019362 | pyridine nucleotide metabolic process | 29 | 2 | 0.020541 |
| GO:0007310 | oocyte dorsal/ventral axis specification | 87 | 3 | 0.021404 |
| GO:0008154 | actin polymerization or depolymerization | 27 | 2 | 0.021518 |
| GO:0061058 | regulation of peptidoglycan recognition protein signaling pathway | 4 | 1 | 0.021912 |
| GO:0061060 | negative regulation of peptidoglycan recognition protein signaling pathway | 4 | 1 | 0.021912 |
| GO:0072595 | maintenance of protein localization in organelle | 12 | 2 | 0.021982 |
| GO:0019043 | establishment of viral latency | 42 | 2 | 0.023494 |
| GO:0075713 | establishment of integrated proviral latency | 42 | 2 | 0.023494 |
| GO:0019800 | peptide cross-linking via chondroitin 4-sulfate glycosaminoglycan | 4 | 1 | 0.023898 |
| GO:0075733 | intracellular transport of virus | 42 | 2 | 0.023984 |
| GO:1902583 | multi-organism intracellular transport | 42 | 2 | 0.023984 |
| GO:0044766 | multi-organism transport | 43 | 2 | 0.025118 |
| GO:0046794 | transport of virus | 43 | 2 | 0.025118 |
| GO:1902579 | multi-organism localization | 43 | 2 | 0.025118 |
| GO:0008356 | asymmetric cell division | 59 | 3 | 0.025613 |
| GO:0039694 | viral RNA genome replication | 17 | 2 | 0.028155 |
| GO:0039703 | RNA replication | 17 | 2 | 0.028155 |
| GO:0002218 | activation of innate immune response | 35 | 2 | 0.029274 |
| GO:0006935 | Chemotaxis | 97 | 3 | 0.029804 |
| GO:0072524 | pyridine-containing compound metabolic process | 38 | 2 | 0.030197 |
| GO:0044033 | multi-organism metabolic process | 20 | 2 | 0.033452 |
| GO:0006809 | nitric oxide biosynthetic process | 5 | 1 | 0.034261 |
| GO:0061157 | mRNA destabilization | 6 | 1 | 0.034917 |
| GO:0043624 | cellular protein complex disassembly | 39 | 2 | 0.035191 |
| GO:0048053 | R1/R6 development | 6 | 1 | 0.035357 |
| GO:0048750 | compound eye corneal lens morphogenesis | 6 | 1 | 0.035357 |
| GO:0045920 | negative regulation of exocytosis | 5 | 1 | 0.035374 |
| GO:0007309 | oocyte axis specification | 101 | 3 | 0.035892 |
| GO:0006733 | oxidoreduction coenzyme metabolic process | 42 | 2 | 0.035952 |
| GO:2001222 | regulation of neuron migration | 3 | 1 | 0.03765 |
| GO:0048469 | cell maturation | 40 | 2 | 0.038252 |
| GO:0030155 | regulation of cell adhesion | 170 | 4 | 0.038458 |
| GO:0010927 | cellular component assembly involved in morphogenesis | 176 | 5 | 0.038545 |
| GO:0048813 | dendrite morphogenesis | 255 | 5 | 0.038688 |
| GO:1900425 | negative regulation of defense response to bacterium | 7 | 1 | 0.038725 |
| GO:0050779 | RNA destabilization | 7 | 1 | 0.041235 |
| GO:0019079 | viral genome replication | 26 | 2 | 0.041574 |
| GO:0031282 | regulation of guanylate cyclase activity | 7 | 1 | 0.042349 |
| GO:0031284 | positive regulation of guanylate cyclase activity | 7 | 1 | 0.042349 |
| GO:0010840 | regulation of circadian sleep/wake cycle, wakefulness | 6 | 1 | 0.042913 |
| GO:0010841 | positive regulation of circadian sleep/wake cycle, wakefulness | 6 | 1 | 0.042913 |
| GO:0030260 | entry into host cell | 56 | 2 | 0.043039 |
| GO:0044409 | entry into host | 56 | 2 | 0.043039 |
| GO:0046718 | viral entry into host cell | 56 | 2 | 0.043039 |
| GO:0051806 | entry into cell of other organism involved in symbiotic interaction | 56 | 2 | 0.043039 |
| GO:0051828 | entry into other organism involved in symbiotic interaction | 56 | 2 | 0.043039 |
| GO:0052126 | movement in host environment | 56 | 2 | 0.043039 |
| GO:0052192 | movement in environment of other organism involved in symbiotic interaction | 56 | 2 | 0.043039 |
| GO:0001709 | cell fate determination | 166 | 4 | 0.043683 |
| GO:0002757 | immune response-activating signal transduction | 50 | 2 | 0.043724 |
| GO:0040017 | positive regulation of locomotion | 121 | 3 | 0.045874 |
| GO:0019076 | viral release from host cell | 60 | 2 | 0.045979 |
| GO:0007154 | cell communication | 579 | 8 | 0.046842 |
| GO:0043241 | protein complex disassembly | 45 | 2 | 0.046973 |
| GO:0007263 | nitric oxide mediated signal transduction | 8 | 1 | 0.047454 |
| GO:0007015 | actin filament organization | 182 | 4 | 0.047794 |

Table S4. Molecular function GO term upregulated in control-mated females when compared with virgin females

|  | GO Function | Total numbers in category | Number differentially expressed | P value |
| --- | --- | --- | --- | --- |
| GO:0008307 | structural constituent of muscle | 40 | 12 | 1.69E-12 |
| GO:0003779 | actin binding | 195 | 15 | 1.63E-09 |
| GO:0008092 | cytoskeletal protein binding | 395 | 17 | 1.6E-07 |
| GO:0005198 | structural molecule activity | 520 | 17 | 2.59E-07 |
| GO:0016709 | oxidoreductase activity | 11 | 3 | 0.000172 |
| GO:0001872 | (1->3)-beta-D-glucan binding | 5 | 2 | 0.000343 |
| GO:0043914 | NADPH:sulfur oxidoreductase activity | 3 | 2 | 0.001285 |
| GO:0004521 | endoribonuclease activity | 137 | 5 | 0.001533 |
| GO:0004540 | ribonuclease activity | 156 | 5 | 0.002875 |
| GO:0004523 | RNA-DNA hybrid ribonuclease activity | 105 | 4 | 0.003011 |
| GO:0001530 | lipopolysaccharide binding | 15 | 2 | 0.003744 |
| GO:0001871 | pattern binding | 15 | 2 | 0.004603 |
| GO:0030247 | polysaccharide binding | 15 | 2 | 0.004603 |
| GO:0008329 | signaling pattern recognition receptor activity | 19 | 2 | 0.005421 |
| GO:0038187 | pattern recognition receptor activity | 19 | 2 | 0.005421 |
| GO:0016891 | endoribonuclease activity, producing 5'-phosphomonoesters | 120 | 4 | 0.006138 |
| GO:0071949 | FAD binding | 13 | 2 | 0.006505 |
| GO:0016893 | endonuclease activity, active with either ribo- or deoxyribonucleic acids | 128 | 4 | 0.007679 |
| GO:0042803 | protein homodimerization activity | 403 | 8 | 0.007917 |
| GO:0051015 | actin filament binding | 47 | 3 | 0.013265 |
| GO:0005521 | lamin binding | 7 | 2 | 0.014507 |
| GO:0035925 | mRNA 3'-UTR AU-rich region binding | 3 | 1 | 0.018249 |
| GO:0004517 | nitric-oxide synthase activity | 3 | 1 | 0.021198 |
| GO:0004040 | amidase activity | 4 | 1 | 0.021912 |
| GO:0015020 | glucuronosyltransferase activity | 39 | 2 | 0.022887 |
| GO:0001078 | RNA polymerase II core promoter proximal region sequence-specific DNA binding transcription factor activity involved in negative regulation of transcription | 41 | 2 | 0.025333 |
| GO:0050660 | flavin adenine dinucleotide binding | 96 | 3 | 0.027 |
| GO:0001227 | RNA polymerase II transcription - negative regulation of transcription | 51 | 2 | 0.036898 |
| GO:0003968 | RNA-directed RNA polymerase activity | 20 | 2 | 0.037575 |
| GO:0046983 | protein dimerization activity | 571 | 8 | 0.043419 |
| GO:0003887 | DNA-directed DNA polymerase activity | 129 | 3 | 0.044997 |
| GO:0001786 | phosphatidylserine binding | 7 | 1 | 0.048209 |
| GO:0017048 | Rho GTPase binding | 23 | 3 | 0.049818 |

Table S5. Cellular component GO term upregulated in control-mated females when compared with virgin females

|  | GO Function | Total numbers in category | Number differentially expressed | P value |
| --- | --- | --- | --- | --- |
| GO:0000794 | condensed nuclear chromosome | 31 | 10 | 0 |
| GO:0005859 | muscle myosin complex | 23 | 11 | 0 |
| GO:0005863 | striated muscle myosin thick filament | 17 | 10 | 0 |
| GO:0016460 | myosin II complex | 28 | 11 | 0 |
| GO:0031674 | I band | 23 | 11 | 0 |
| GO:0032982 | myosin filament | 30 | 11 | 0 |
| GO:0036379 | Myofilament | 26 | 11 | 0 |
| GO:0005576 | extracellular region | 790 | 29 | 1.67E-15 |
| GO:0030017 | Sarcomere | 40 | 13 | 1.93E-14 |
| GO:0044449 | contractile fiber part | 122 | 17 | 6.96E-12 |
| GO:0016459 | myosin complex | 70 | 12 | 1.52E-11 |
| GO:0000793 | condensed chromosome | 39 | 10 | 1.84E-11 |
| GO:0030018 | Z disc | 75 | 11 | 1.53E-09 |
| GO:0000228 | nuclear chromosome | 70 | 10 | 2.02E-09 |
| GO:0005875 | microtubule associated complex | 344 | 14 | 8.83E-07 |
| GO:0005694 | Chromosome | 241 | 12 | 2.66E-06 |
| GO:0048788 | presynaptic cytoskeletal matrix assembled at active zones | 7 | 3 | 1.36E-05 |
| GO:0030863 | cortical cytoskeleton | 24 | 3 | 0.000492 |
| GO:0044423 | virion part | 54 | 4 | 0.000658 |
| GO:0019028 | viral capsid | 17 | 3 | 0.000751 |
| GO:0033646 | host intracellular part | 56 | 4 | 0.000939 |
| GO:0042600 | Chorion | 8 | 2 | 0.001028 |
| GO:0044430 | cytoskeletal part | 865 | 16 | 0.001503 |
| GO:0033643 | host cell part | 65 | 4 | 0.001733 |
| GO:0044217 | other organism part | 65 | 4 | 0.001733 |
| GO:0031672 | A band | 33 | 5 | 0.001835 |
| GO:0048786 | presynaptic active zone | 42 | 3 | 0.002569 |
| GO:0042734 | presynaptic membrane | 44 | 3 | 0.002695 |
| GO:0030312 | external encapsulating structure | 15 | 2 | 0.003694 |
| GO:0030430 | host cell cytoplasm | 43 | 3 | 0.0055 |
| GO:0044456 | synapse part | 376 | 8 | 0.005968 |
| GO:0043025 | neuronal cell body | 181 | 5 | 0.006616 |
| GO:0031430 | M band | 21 | 4 | 0.008372 |
| GO:0005578 | proteinaceous extracellular matrix | 125 | 4 | 0.010005 |
| GO:0097060 | synaptic membrane | 182 | 5 | 0.010123 |
| GO:0030424 | Axon | 160 | 5 | 0.010388 |
| GO:0044297 | cell body | 204 | 5 | 0.010526 |
| GO:0030895 | apolipoprotein B mRNA editing enzyme complex | 2 | 1 | 0.010968 |
| GO:0034993 | SUN-KASH complex | 6 | 2 | 0.013217 |
| GO:0005640 | nuclear outer membrane | 8 | 2 | 0.016179 |
| GO:0031594 | neuromuscular junction | 77 | 3 | 0.016525 |
| GO:0005783 | endoplasmic reticulum | 426 | 7 | 0.021258 |
| GO:0044174 | host cell endosome | 4 | 1 | 0.021551 |
| GO:0044184 | host cell late endosome | 4 | 1 | 0.021551 |
| GO:0072494 | host multivesicular body | 4 | 1 | 0.021551 |
| GO:0033647 | host intracellular organelle | 41 | 2 | 0.023422 |
| GO:0033648 | host intracellular membrane-bounded organelle | 41 | 2 | 0.023422 |
| GO:0042025 | host cell nucleus | 41 | 2 | 0.023422 |
| GO:0031012 | extracellular matrix | 164 | 4 | 0.027446 |
| GO:0005614 | interstitial matrix | 5 | 1 | 0.02983 |
| GO:0055036 | virion membrane | 7 | 1 | 0.038108 |

Table S6. GO terms down-regulated in control-mated females when compared with virgin females (BP, Biological processes; MF, Molecular functions; CC, Cellular component)

|  | Cate-gory | GO Function | Total numbers in category | Number differentially expressed | P value |
| --- | --- | --- | --- | --- | --- |
| GO:0003674 | MF | molecular function | 11752 | 62 | 7.42E-07 |
| GO:0016020 | CC | membrane | 3753 | 12 | 0.000259 |
| GO:0043227 | CC | membrane-bounded organelle | 5567 | 23 | 0.000416 |
| GO:0044464 | CC | cell part | 10245 | 57 | 0.00056 |
| GO:0051246 | BP | regulation of protein metabolic process | 807 | 0 | 0.000975 |
| GO:0031323 | BP | regulation of cellular metabolic process | 2804 | 9 | 0.00132 |
| GO:0032268 | BP | regulation of cellular protein metabolic process | 756 | 0 | 0.001448 |
| GO:0044248 | BP | cellular catabolic process | 870 | 0 | 0.002026 |
| GO:0043231 | CC | intracellular membrane-bounded organelle | 5261 | 23 | 0.002085 |
| GO:0005886 | CC | plasma membrane | 1655 | 3 | 0.002407 |
| GO:0044711 | BP | single-organism biosynthetic process | 903 | 0 | 0.002672 |
| GO:0080090 | BP | regulation of primary metabolic process | 2660 | 9 | 0.002726 |
| GO:0022892 | MF | substrate-specific transporter activity | 883 | 0 | 0.002882 |
| GO:0031982 | CC | vesicle | 992 | 1 | 0.003442 |
| GO:0009056 | BP | catabolic process | 1118 | 1 | 0.003689 |
| GO:0005737 | CC | cytoplasm | 2727 | 10 | 0.004576 |
| GO:0019538 | BP | protein metabolic process | 2370 | 8 | 0.004911 |
| GO:0031988 | CC | membrane-bounded vesicle | 933 | 1 | 0.005007 |
| GO:0005215 | MF | transporter activity | 1072 | 1 | 0.005253 |
| GO:0005634 | CC | nucleus | 3432 | 13 | 0.005385 |
| GO:0033036 | BP | macromolecule localization | 901 | 1 | 0.005483 |
| GO:0006811 | BP | ion transport | 784 | 0 | 0.006089 |
| GO:0005739 | CC | mitochondrion | 763 | 0 | 0.006402 |
| GO:0022891 | MF | substrate-specific transmembrane transporter activity | 791 | 0 | 0.006853 |
| GO:0006629 | BP | lipid metabolic process | 696 | 0 | 0.007544 |
| GO:0043230 | CC | extracellular organelle | 635 | 0 | 0.007825 |
| GO:1903561 | NA | NA | 635 | 0 | 0.007825 |
| GO:0008104 | BP | protein localization | 857 | 1 | 0.007925 |
| GO:0031226 | CC | intrinsic component of plasma membrane | 706 | 0 | 0.008023 |
| GO:0065010 | CC | extracellular membrane-bounded organelle | 629 | 0 | 0.008238 |
| GO:0070062 | CC | extracellular vesicular exosome | 629 | 0 | 0.008238 |
| GO:0044267 | BP | cellular protein metabolic process | 1818 | 6 | 0.009667 |
| GO:0071702 | BP | organic substance transport | 1054 | 2 | 0.009838 |
| GO:0044765 | BP | single-organism transport | 1937 | 6 | 0.01006 |
| GO:0005887 | CC | integral component of plasma membrane | 664 | 0 | 0.010961 |
| GO:0051716 | BP | cellular response to stimulus | 1081 | 2 | 0.011488 |
| GO:1901575 | BP | organic substance catabolic process | 929 | 1 | 0.011698 |
| GO:0043412 | BP | macromolecule modification | 1539 | 5 | 0.011704 |
| GO:0030529 | CC | ribonucleoprotein complex | 584 | 0 | 0.011927 |
| GO:0044699 | BP | single-organism process | 8413 | 48 | 0.012423 |
| GO:0015075 | MF | ion transmembrane transporter activity | 696 | 0 | 0.012545 |
| GO:0051179 | BP | localization | 2714 | 11 | 0.012682 |
| GO:0042592 | BP | homeostatic process | 614 | 0 | 0.013102 |
| GO:0051726 | BP | regulation of cell cycle | 499 | 0 | 0.013776 |
| GO:0006810 | BP | transport | 2397 | 9 | 0.014224 |
| GO:0019222 | BP | regulation of metabolic process | 3216 | 15 | 0.015009 |
| GO:0003824 | MF | catalytic activity | 5755 | 28 | 0.015134 |
| GO:0044237 | BP | cellular metabolic process | 6540 | 34 | 0.016254 |
| GO:0060255 | BP | regulation of macromolecule metabolic process | 2773 | 12 | 0.018295 |
| GO:0006464 | BP | cellular protein modification process | 1435 | 5 | 0.019935 |
| GO:0036211 | BP | protein modification process | 1435 | 5 | 0.019935 |
| GO:0022857 | MF | transmembrane transporter activity | 915 | 1 | 0.020015 |
| GO:0044424 | CC | intracellular part | 8928 | 53 | 0.021274 |
| GO:0043167 | MF | ion binding | 4881 | 25 | 0.021984 |
| GO:0044712 | BP | single-organism catabolic process | 585 | 0 | 0.022693 |
| GO:0031399 | BP | regulation of protein modification process | 457 | 0 | 0.02302 |
| GO:0044425 | CC | membrane part | 3809 | 17 | 0.023167 |
| GO:1902578 | BP | single-organism localization | 2083 | 8 | 0.02435 |
| GO:0009889 | BP | regulation of biosynthetic process | 2100 | 8 | 0.025846 |
| GO:0051130 | BP | positive regulation of cellular component organization | 478 | 0 | 0.026443 |
| GO:0005509 | MF | calcium ion binding | 444 | 0 | 0.026681 |
| GO:0031326 | BP | regulation of cellular biosynthetic process | 2089 | 8 | 0.02716 |
| GO:0042325 | BP | regulation of phosphorylation | 448 | 0 | 0.027344 |
| GO:0010604 | BP | positive regulation of macromolecule metabolic process | 1150 | 3 | 0.030033 |
| GO:0045184 | BP | establishment of protein localization | 635 | 1 | 0.033136 |
| GO:0070647 | BP | protein modification by small protein conjugation or removal | 386 | 0 | 0.033399 |
| GO:1902494 | CC | catalytic complex | 695 | 1 | 0.033434 |
| GO:0044255 | BP | cellular lipid metabolic process | 519 | 0 | 0.033456 |
| GO:1901576 | BP | organic substance biosynthetic process | 2877 | 12 | 0.033794 |
| GO:0051247 | BP | positive regulation of protein metabolic process | 432 | 0 | 0.034869 |
| GO:0051093 | BP | negative regulation of developmental process | 447 | 0 | 0.034901 |
| GO:0006812 | BP | cation transport | 528 | 0 | 0.035648 |
| GO:0008152 | BP | metabolic process | 7511 | 42 | 0.035825 |
| GO:0009987 | BP | cellular process | 9329 | 56 | 0.036043 |
| GO:0035556 | BP | intracellular signal transduction | 662 | 1 | 0.037429 |
| GO:0005488 | MF | binding | 8977 | 54 | 0.037584 |
| GO:0030234 | MF | enzyme regulator activity | 419 | 0 | 0.038994 |
| GO:0015031 | BP | protein transport | 606 | 1 | 0.039552 |
| GO:0032270 | BP | positive regulation of cellular protein metabolic process | 411 | 0 | 0.040183 |
| GO:0051234 | BP | establishment of localization | 2455 | 11 | 0.040381 |
| GO:0010556 | BP | regulation of macromolecule biosynthetic process | 1990 | 8 | 0.040493 |
| GO:0044431 | CC | Golgi apparatus part | 423 | 0 | 0.042088 |
| GO:2001141 | BP | regulation of RNA biosynthetic process | 1801 | 7 | 0.043259 |
| GO:0044249 | BP | cellular biosynthetic process | 2805 | 12 | 0.043547 |
| GO:1903506 | NA | NA | 1799 | 7 | 0.043555 |
| GO:0048522 | BP | positive regulation of cellular process | 2084 | 9 | 0.043925 |
| GO:2000112 | BP | regulation of cellular macromolecule biosynthetic process | 1969 | 8 | 0.04428 |
| GO:0070727 | BP | cellular macromolecule localization | 403 | 0 | 0.0447 |
| GO:0006355 | BP | regulation of transcription, DNA-templated | 1792 | 7 | 0.044967 |
| GO:0008324 | MF | cation transmembrane transporter activity | 490 | 0 | 0.046669 |
| GO:0051171 | BP | regulation of nitrogen compound metabolic process | 2127 | 9 | 0.047278 |
| GO:0071704 | BP | organic substance metabolic process | 6970 | 39 | 0.04736 |
| GO:1901700 | BP | response to oxygen-containing compound | 424 | 0 | 0.047606 |
| GO:0009058 | BP | biosynthetic process | 2958 | 13 | 0.048219 |
| GO:0051241 | BP | negative regulation of multicellular organismal process | 399 | 0 | 0.048358 |
| GO:0031325 | BP | positive regulation of cellular metabolic process | 1263 | 4 | 0.049036 |

Table S7. Genes up-regulated in lure-mated females when compared with virgin females

|  | PValue | FDR | function | reference |
| --- | --- | --- | --- | --- |
| TRINITY_DN47020_c0_g2 | 2.70E-39 | 2.26E-34 | polyprotein | B dorsalis, c. capitata |
| TRINITY_DN11163_c0_g1 | 4.77E-29 | 1.33E-24 | unknown | unknown |
| TRINITY_DN36607_c0_g1 | 9.81E-29 | 2.06E-24 | unknown | unknown |
| TRINITY_DN38387_c0_g1 | 5.34E-26 | 8.95E-22 | lysozyme | B dorsalis, D melano |
| TRINITY_DN35577_c0_g1 | 7.11E-25 | 8.51E-21 | unknown | C capitata, D melano |
| TRINITY_DN36898_c0_g1 | 1.23E-23 | 1.29E-19 | unknown | B dorsalis |
| TRINITY_DN31168_c0_g1 | 1.63E-22 | 1.37E-18 | Unknown | unknown |
| TRINITY_DN41747_c0_g1 | 1.57E-21 | 1.20E-17 | Unknown | B dorsalis |
| TRINITY_DN18198_c0_g2 | 8.16E-21 | 5.70E-17 | trehalose transporter | B dorsalis |
| TRINITY_DN23355_c0_g1 | 1.05E-20 | 6.75E-17 | S-methyl-5'-thioadenosine phosphorylase | B dorsalis, D melano |
| TRINITY_DN26791_c0_g1 | 4.22E-19 | 2.53E-15 | Unknown | B dorsalis, c. capitata |
| TRINITY_DN37197_c0_g1 | 4.75E-18 | 2.51E-14 | Unknown | unknown |
| TRINITY_DN39613_c0_g1 | 4.79E-18 | 2.51E-14 | Alcohol dehydrogenase - oxido reductase activity | B dorsalis |
| TRINITY_DN34577_c0_g1 | 8.08E-18 | 3.98E-14 | Unknown | unknown |
| TRINITY_DN32691_c0_g1 | 2.99E-17 | 1.39E-13 | Unknown | B dorsalis |
| TRINITY_DN23140_c0_g1 | 3.26E-16 | 1.37E-12 | Unknown | unknown |
| TRINITY_DN31576_c0_g1 | 3.55E-16 | 1.42E-12 | Unknown | unknown |
| TRINITY_DN30147_c0_g1 | 4.84E-16 | 1.71E-12 | Unknown | B dorsalis |
| TRINITY_DN39825_c0_g2 | 8.14E-16 | 2.69E-12 | cytosol aminopeptidase | C capitata |
| TRINITY_DN47020_c0_g1 | 1.10E-15 | 3.41E-12 | Unknown | unknown |
| TRINITY_DN40623_c0_g1 | 3.76E-15 | 1.05E-11 | Unknown | unknown |
| TRINITY_DN47122_c0_g1 | 4.70E-15 | 1.27E-11 | Replicase polyprotein | B dorsalis, c. capitata |
| TRINITY_DN31642_c0_g1 | 7.87E-15 | 2.06E-11 | Unknown | B dorsalis |
| TRINITY_DN31574_c0_g1 | 9.48E-15 | 2.41E-11 | Unknown | unknown |
| TRINITY_DN32536_c0_g1 | 1.21E-13 | 2.90E-10 | Unknown | B dorsalis |
| TRINITY_DN47162_c0_g1 | 1.73E-13 | 3.97E-10 | Unknown | B dorsalis |
| TRINITY_DN38781_c0_g1 | 2.66E-13 | 5.88E-10 | chorion protein | B dorsalis, c. capitata |
| TRINITY_DN26915_c0_g1 | 3.38E-13 | 7.27E-10 | Unknown | unknown |
| TRINITY_DN41053_c1_g2 | 3.72E-13 | 7.79E-10 | Unknown | unknown |
| TRINITY_DN33101_c0_g1 | 4.63E-13 | 9.47E-10 | Unknown | unknown |
| TRINITY_DN46012_c0_g1 | 5.66E-13 | 1.13E-09 | H3 acetylation, Histone exchange, DNA repair, peptidase - histone acetyl transferase | D melano |
| TRINITY_DN33970_c0_g1 | 7.49E-13 | 1.42E-09 | Unknown | unknown |
| TRINITY_DN64100_c0_g1 | 7.63E-13 | 1.42E-09 | ATP synthase | D melano |
| TRINITY_DN17139_c0_g1 | 8.02E-13 | 1.46E-09 | Unknown | unknown |
| TRINITY_DN46263_c0_g1 | 9.96E-13 | 1.78E-09 | Titin - ID like | B dorsalis, D melano |
| TRINITY_DN35504_c0_g1 | 1.13E-12 | 1.97E-09 | Unknown | B dorsalis |
| TRINITY_DN35156_c0_g3 | 8.94E-12 | 1.50E-08 | Unknown | unknown |
| TRINITY_DN42906_c0_g8 | 1.21E-11 | 1.99E-08 | Unknown | unknown |
| TRINITY_DN18460_c0_g1 | 2.18E-11 | 3.51E-08 | Unknown | B dorsalos |
| TRINITY_DN46908_c0_g1 | 4.06E-11 | 6.42E-08 | Titin - immunoglobulin domain | B dorsalis, D melano |
| TRINITY_DN21170_c0_g1 | 4.98E-11 | 7.73E-08 | Unknown | unknown |
| TRINITY_DN40151_c0_g2 | 7.85E-11 | 1.18E-07 | Unknown | unknown |
| TRINITY_DN45801_c0_g1 | 1.40E-10 | 2.05E-07 | Unknown | B dorsalis |
| TRINITY_DN32162_c0_g1 | 1.49E-10 | 2.16E-07 | Purine nucleoside phosphorylase | B dorsalis |
| TRINITY_DN44156_c1_g1 | 1.73E-10 | 2.46E-07 | Unknown | B dorsalis, c. capitata |
| TRINITY_DN33239_c0_g1 | 2.69E-10 | 3.64E-07 | Alcohol dehydrogenase transcription | B dorsalis |
| TRINITY_DN33196_c0_g1 | 3.18E-10 | 4.23E-07 | Unknown | C capitata |
| TRINITY_DN40180_c0_g5 | 3.33E-10 | 4.36E-07 | Immune response | Sacrophaga |
| TRINITY_DN22480_c0_g1 | 3.41E-10 | 4.40E-07 | Unknown | B dorsalis |
| TRINITY_DN18872_c0_g1 | 3.62E-10 | 4.60E-07 | Unknown | C capitata |
| TRINITY_DN38331_c0_g1 | 3.91E-10 | 4.90E-07 | chorion protein | C capitata, D grimshawi |
| TRINITY_DN45025_c0_g1 | 4.24E-10 | 5.23E-07 | Unknown | B dorsalis |
| TRINITY_DN66196_c0_g1 | 4.68E-10 | 5.69E-07 | Unknown | unknown |
| TRINITY_DN44600_c0_g1 | 6.13E-10 | 7.24E-07 | Unknown | B dorsalis |
| TRINITY_DN33640_c0_g1 | 6.13E-10 | 7.24E-07 | Unknown | unknown |
| TRINITY_DN45697_c1_g1 | 7.55E-10 | 8.67E-07 | Titin | B dorsalis |
| TRINITY_DN20441_c0_g1 | 1.38E-09 | 1.54E-06 | Unknown | unknown |
| TRINITY_DN44948_c0_g1 | 1.45E-09 | 1.59E-06 | Protein phosphorylation | Caenorhabditis |
| TRINITY_DN72019_c0_g1 | 1.46E-09 | 1.59E-06 | Unknown | B dorsalis |
| TRINITY_DN32401_c0_g1 | 1.59E-09 | 1.71E-06 | chorion family | B dorsalis, D melano |
| TRINITY_DN34372_c0_g1 | 1.79E-09 | 1.90E-06 | Unknown | B dorsalis |
| TRINITY_DN24697_c0_g1 | 2.31E-09 | 2.43E-06 | Unknown | B dorsalis |
| TRINITY_DN28099_c0_g1 | 2.70E-09 | 2.80E-06 | Unknown | B dorsalis |
| TRINITY_DN36289_c0_g4 | 2.81E-09 | 2.88E-06 | Unknown | unknown |
| TRINITY_DN2552_c0_g1 | 3.32E-09 | 3.36E-06 | Unknown | B dorsalis |
| TRINITY_DN31428_c1_g1 | 3.73E-09 | 3.72E-06 | Unknown | unknown |
| TRINITY_DN18047_c0_g1 | 5.32E-09 | 5.19E-06 | Unknown | unknown |
| TRINITY_DN19937_c0_g1 | 7.17E-09 | 6.91E-06 | Unknown | B dorsalis |
| TRINITY_DN44600_c0_g2 | 8.10E-09 | 7.72E-06 | Titin | B dorsalis, D melano |
| TRINITY_DN57009_c0_g1 | 9.84E-09 | 9.28E-06 | Unknown | unknown |
| TRINITY_DN31807_c1_g1 | 1.01E-08 | 9.38E-06 | Unknown | unknown |
| TRINITY_DN31826_c0_g1 | 1.39E-08 | 1.27E-05 | Unknown | unknown |
| TRINITY_DN37787_c0_g1 | 1.43E-08 | 1.29E-05 | Unknown | B dorsalis |
| TRINITY_DN6705_c0_g1 | 1.48E-08 | 1.29E-05 | Spermatogenesis | C capitata, Anthonomous grandis |
| TRINITY_DN34007_c0_g1 | 1.55E-08 | 1.32E-05 | lysozyme activity - defense response to bacterium | B dorsalis, D melano, M domestica |
| TRINITY_DN62012_c0_g1 | 2.02E-08 | 1.71E-05 | Unknown | unknown |
| TRINITY_DN30781_c0_g1 | 2.37E-08 | 1.99E-05 | Unknown | B dorsalis |
| TRINITY_DN46861_c1_g2 | 2.74E-08 | 2.28E-05 | Unknown | B dorsalis |
| TRINITY_DN62542_c0_g1 | 3.01E-08 | 2.47E-05 | Sperm flagellar protein | B dorsalis, Bovinae |
| TRINITY_DN40177_c0_g1 | 3.36E-08 | 2.73E-05 | Unknown | B dorsalis |
| TRINITY_DN47273_c0_g1 | 3.39E-08 | 2.73E-05 | Unknown | unknown |
| TRINITY_DN54510_c0_g1 | 3.84E-08 | 3.01E-05 | Unknown | unknown |
| TRINITY_DN40180_c0_g2 | 5.13E-08 | 3.98E-05 | Unknown | unknown |
| TRINITY_DN41533_c0_g1 | 5.82E-08 | 4.48E-05 | Unknown | C capitata |
| TRINITY_DN32435_c0_g1 | 6.92E-08 | 5.28E-05 | Chorion protein | B dorsalis, D melano |
| TRINITY_DN28122_c0_g1 | 7.76E-08 | 5.86E-05 | Histone H1 | B dorsalis, chronomus |
| TRINITY_DN36373_c0_g1 | 8.04E-08 | 6.02E-05 | Gag polyprotein | B dorsalis, D melano |
| TRINITY_DN46869_c0_g1 | 9.01E-08 | 6.69E-05 | Titin | C capitata, D melano |
| TRINITY_DN44749_c0_g2 | 1.17E-07 | 8.44E-05 | Unknown | B dorsalis |
| TRINITY_DN57008_c0_g1 | 1.22E-07 | 8.73E-05 | Unknown | B dorsalis |
| TRINITY_DN62226_c0_g1 | 1.90E-07 | 0.000135 | Unknown | B dorsalis |
| TRINITY_DN23905_c0_g1 | 1.93E-07 | 0.000136 | Unknown | unknown |
| TRINITY_DN44950_c0_g1 | 1.95E-07 | 0.000136 | Unknown | B dorsalis |
| TRINITY_DN42232_c0_g1 | 1.99E-07 | 0.000138 | Farnesol dehydrogenase | B dorsalis, Aedes agepti |
| TRINITY_DN18565_c0_g1 | 2.14E-07 | 0.000147 | Unknown | unknown |
| TRINITY_DN16881_c0_g1 | 2.54E-07 | 0.000173 | Unknown | B dorsalis |
| TRINITY_DN47197_c1_g1 | 2.88E-07 | 0.000195 | Nesprin 1 - Spectrin - protein binding | B dorsalis, mouse |
| TRINITY_DN70106_c0_g1 | 2.95E-07 | 0.000198 | Unknown | C capitata |
| TRINITY_DN24347_c0_g1 | 3.02E-07 | 0.000201 | Unknown | B dorsalis |
| TRINITY_DN44156_c0_g1 | 3.18E-07 | 0.00021 | Unknown | B dorsalis, C capitata |
| TRINITY_DN45697_c0_g1 | 3.41E-07 | 0.000223 | Titin | B dorsalis, D melano |
| TRINITY_DN45128_c0_g2 | 3.46E-07 | 0.000225 | Unknown | B dorsalis |
| TRINITY_DN45717_c0_g4 | 4.93E-07 | 0.000318 | Unknown | unknown |
| TRINITY_DN17743_c0_g1 | 5.85E-07 | 0.000369 | Unknown | C capitata |
| TRINITY_DN7873_c0_g1 | 5.85E-07 | 0.000369 | Unknown | B dorsalis |
| TRINITY_DN30209_c0_g1 | 6.18E-07 | 0.000387 | Unknown | B dorsalis |
| TRINITY_DN24567_c0_g1 | 6.53E-07 | 0.000406 | Unknown | B dorsalis |
| TRINITY_DN47898_c0_g1 | 6.82E-07 | 0.000417 | Unknown | B dorsalis |
| TRINITY_DN9593_c0_g1 | 7.04E-07 | 0.000428 | Unknown | B dorsalis |
| TRINITY_DN1536_c0_g1 | 7.47E-07 | 0.00045 | Testis expressed | B dorsalis, mouse |
| TRINITY_DN15832_c0_g2 | 7.79E-07 | 0.000466 | P-selectin, lectin c type | B doarsalis, sheep |
| TRINITY_DN29460_c0_g1 | 8.10E-07 | 0.000482 | Unknown | B doarsalis |
| TRINITY_DN9774_c0_g1 | 8.36E-07 | 0.000493 | Unknown | B dorsalis |
| TRINITY_DN39977_c0_g1 | 8.42E-07 | 0.000493 | Unknown | B dorsalis |
| TRINITY_DN46557_c0_g1 | 9.23E-07 | 0.000534 | Titin | B dorsalis, D melano |
| TRINITY_DN47233_c0_g1 | 9.49E-07 | 0.000541 | Titin | D melano |
| TRINITY_DN73333_c0_g1 | 1.01E-06 | 0.000572 | Unknown | B dorsalis |
| TRINITY_DN1374_c0_g1 | 1.09E-06 | 0.000614 | Unknown | unknown |
| TRINITY_DN47197_c1_g2 | 1.33E-06 | 0.000731 | Nesprin1 | B dorsalis, human |
| TRINITY_DN34179_c0_g1 | 1.39E-06 | 0.000764 | Outer dense fibre of sperm tail | B dorsalis, Danio |
| TRINITY_DN46861_c1_g1 | 1.42E-06 | 0.000773 | Unknown | unknown |
| TRINITY_DN66643_c0_g1 | 1.54E-06 | 0.000833 | Unknown | unknown |
| TRINITY_DN27028_c0_g1 | 1.57E-06 | 0.000843 | Uridine phosphorylate | B dorsalis, mouse |
| TRINITY_DN52523_c0_g1 | 1.77E-06 | 0.000937 | Unknown | C capitata |

Table S8. Genes down-regulated in lure-mated females when compared with virgin females

|  | PValue | FDR | function | reference |
| --- | --- | --- | --- | --- |
| TRINITY_DN37216_c0_g1 | 1.35E-29 | 5.66E-25 | Adult cuticle protein | B dorsalis, D melano |
| TRINITY_DN18302_c0_g2 | 5.17E-25 | 7.22E-21 | Adult cuticle protein | C capitata, D melano |
| TRINITY_DN43019_c1_g1 | 4.88E-23 | 4.54E-19 | Unknown | unknown |
| TRINITY_DN39591_c0_g1 | 2.02E-16 | 8.90E-13 | Adult cuticle protein | B dorsalis, D melano |
| TRINITY_DN21589_c0_g1 | 3.91E-16 | 1.49E-12 | Genome polyprotein | C capitata |
| TRINITY_DN30500_c0_g1 | 4.89E-16 | 1.71E-12 | Adult cuticle protein | B dorsalis, D melano |
| TRINITY_DN43052_c0_g1 | 8.33E-16 | 2.69E-12 | Unknown | B dorsalis |
| TRINITY_DN26618_c0_g1 | 1.42E-15 | 4.25E-12 | Unknown | C capitata |
| TRINITY_DN33947_c0_g1 | 3.62E-15 | 1.05E-11 | Adult cuticle protein | B dorsalis, Blaberus craniifer |
| TRINITY_DN8487_c0_g2 | 1.19E-13 | 2.90E-10 | Unknown | C capitata |
| TRINITY_DN40205_c0_g1 | 1.75E-13 | 3.97E-10 | Unknown | B dorsalis |
| TRINITY_DN36289_c0_g1 | 6.90E-13 | 1.35E-09 | Unknown | unknown |
| TRINITY_DN15988_c0_g3 | 5.23E-12 | 8.95E-09 | Unknown | unknown |
| TRINITY_DN19931_c0_g1 | 7.37E-11 | 1.12E-07 | Unknown | unknown |
| TRINITY_DN18302_c0_g1 | 1.99E-10 | 2.78E-07 | Unknown | unknown |
| TRINITY_DN4240_c0_g1 | 2.07E-10 | 2.85E-07 | Unknown | C capitata |
| TRINITY_DN1323_c0_g1 | 6.62E-10 | 7.71E-07 | Unknown | unknown |
| TRINITY_DN20065_c0_g2 | 7.84E-10 | 8.88E-07 | Unknown | unknown |
| TRINITY_DN32739_c0_g1 | 4.06E-09 | 4.00E-06 | Unknown | unknown |
| TRINITY_DN34586_c0_g1 | 1.38E-08 | 1.27E-05 | Zinc finger protein | B dorsalis |
| TRINITY_DN24536_c0_g1 | 1.48E-08 | 1.29E-05 | Unknown | B dorsalis |
| TRINITY_DN36441_c0_g2 | 1.48E-08 | 1.29E-05 | Unknown | unknown |
| TRINITY_DN47468_c0_g1 | 1.53E-08 | 1.32E-05 | Unknown | unknown |
| TRINITY_DN40286_c0_g1 | 3.58E-08 | 2.86E-05 | Unknown | unknown |
| TRINITY_DN26719_c0_g1 | 3.74E-08 | 2.96E-05 | Unknown | unknown |
| TRINITY_DN18515_c0_g1 | 1.09E-07 | 8.00E-05 | L26 60s ribosomal protein | D melano |
| TRINITY_DN45154_c2_g8 | 1.15E-07 | 8.39E-05 | Unknown | unknown |
| TRINITY_DN44705_c0_g2 | 5.23E-07 | 0.000335 | Unknown | unknown |
| TRINITY_DN44797_c0_g1 | 6.71E-07 | 0.000414 | Cytochrome 450 309a2 | B dorsalis, D melano |
| TRINITY_DN39841_c0_g1 | 8.72E-07 | 0.000508 | Unknown | B dorsalis |
| TRINITY_DN25836_c0_g1 | 9.36E-07 | 0.000538 | Transposon Ty3-I Gag-Pol polyprotein | yeast, |
| TRINITY_DN1401_c0_g2 | 1.28E-06 | 0.000714 | Unknown | unknown |
| TRINITY_DN42686_c0_g1 | 1.30E-06 | 0.000719 | Chitin binding | B dorsalis |
| TRINITY_DN41455_c0_g2 | 1.70E-06 | 0.00091 | Unknown | unknown |

Table S9. Biological processes GO terms enriched in lure-mated females when compared with virgin females

| **category** | **term** | **Numbers in category** | **Numbers differentially expressed** | **P value** |
| --- | --- | --- | --- | --- |
| GO:0007522 | visceral muscle development | 19 | 8 | 2.38E-12 |
| GO:0030261 | chromosome condensation | 54 | 9 | 2.06E-11 |
| GO:0006323 | DNA packaging | 55 | 9 | 2.19E-11 |
| GO:0016203 | muscle attachment | 37 | 8 | 1.26E-10 |
| GO:0007062 | sister chromatid cohesion | 35 | 8 | 1.34E-10 |
| GO:0007076 | mitotic chromosome condensation | 37 | 8 | 1.58E-10 |
| GO:0007520 | myoblast fusion | 56 | 8 | 7.51E-10 |
| GO:0007519 | skeletal muscle tissue development | 54 | 8 | 8.58E-10 |
| GO:0035206 | regulation of hemocyte proliferation | 54 | 8 | 1.35E-09 |
| GO:0000768 | syncytium formation by plasma membrane fusion | 65 | 8 | 1.56E-09 |
| GO:0006949 | syncytium formation | 66 | 8 | 1.62E-09 |
| GO:0071103 | DNA conformation change | 114 | 9 | 3.07E-09 |
| GO:0014706 | striated muscle tissue development | 66 | 8 | 3.68E-09 |
| GO:0060537 | muscle tissue development | 74 | 8 | 6.12E-09 |
| GO:0045214 | sarcomere organization | 60 | 8 | 8.33E-09 |
| GO:0007498 | mesoderm development | 94 | 8 | 2.61E-08 |
| GO:0007525 | somatic muscle development | 117 | 8 | 3.40E-08 |
| GO:0031032 | actomyosin structure organization | 101 | 8 | 4.73E-08 |
| GO:0007517 | muscle organ development | 134 | 8 | 9.09E-08 |
| GO:0051276 | chromosome organization | 258 | 9 | 6.11E-07 |
| GO:0061061 | muscle structure development | 225 | 8 | 1.67E-06 |
| GO:0030036 | actin cytoskeleton organization | 227 | 10 | 4.61E-06 |
| GO:0030029 | actin filament-based process | 253 | 10 | 6.55E-06 |
| GO:0002682 | regulation of immune system process | 431 | 9 | 2.91E-05 |
| GO:0007010 | cytoskeleton organization | 597 | 12 | 2.95E-05 |
| GO:0042127 | regulation of cell proliferation | 505 | 9 | 3.75E-05 |
| GO:0043094 | cellular metabolic compound salvage | 38 | 3 | 3.89E-05 |
| GO:0051301 | cell division | 332 | 8 | 9.04E-05 |
| GO:0019835 | cytolysis | 10 | 2 | 0.000108 |
| GO:0048646 | anatomical structure formation involved in morphogenesis | 685 | 11 | 0.000187 |
| GO:0009119 | ribonucleoside metabolic process | 142 | 4 | 0.000254 |
| GO:0043101 | purine-containing compound salvage | 15 | 2 | 0.000323 |
| GO:0009116 | nucleoside metabolic process | 156 | 4 | 0.000342 |
| GO:0043174 | nucleoside salvage | 17 | 2 | 0.000385 |
| GO:1903047 | mitotic cell cycle process | 627 | 9 | 0.000387 |
| GO:1901657 | glycosyl compound metabolic process | 170 | 4 | 0.000439 |
| GO:0006259 | DNA metabolic process | 1506 | 11 | 0.00065 |
| GO:1902589 | single-organism organelle organization | 1348 | 14 | 0.000916 |
| GO:0009888 | tissue development | 524 | 8 | 0.000962 |
| GO:0042455 | ribonucleoside biosynthetic process | 83 | 3 | 0.000989 |
| GO:0009163 | nucleoside biosynthetic process | 85 | 3 | 0.001036 |
| GO:1901659 | glycosyl compound biosynthetic process | 87 | 3 | 0.001084 |
| GO:0009166 | nucleotide catabolic process | 30 | 2 | 0.002165 |
| GO:0046128 | purine ribonucleoside metabolic process | 120 | 3 | 0.002202 |
| GO:1901292 | nucleoside phosphate catabolic process | 32 | 2 | 0.00237 |
| GO:0042278 | purine nucleoside metabolic process | 126 | 3 | 0.002436 |
| GO:0019523 | L-idonate metabolic process | 1 | 1 | 0.002968 |
| GO:0046176 | aldonic acid catabolic process | 1 | 1 | 0.002968 |
| GO:0046183 | L-idonate catabolic process | 1 | 1 | 0.002968 |
| GO:0072522 | purine-containing compound biosynthetic process | 128 | 3 | 0.003016 |
| GO:0022402 | cell cycle process | 800 | 9 | 0.003234 |
| GO:0006738 | nicotinamide riboside catabolic process | 2 | 1 | 0.003538 |
| GO:0046495 | nicotinamide riboside metabolic process | 2 | 1 | 0.003538 |
| GO:0070637 | pyridine nucleoside metabolic process | 2 | 1 | 0.003538 |
| GO:0070638 | pyridine nucleoside catabolic process | 2 | 1 | 0.003538 |
| GO:0040011 | locomotion | 780 | 8 | 0.003914 |
| GO:0019520 | aldonic acid metabolic process | 2 | 1 | 0.005008 |
| GO:0006148 | inosine catabolic process | 3 | 1 | 0.005099 |
| GO:0034356 | NAD biosynthesis via nicotinamide riboside salvage pathway | 3 | 1 | 0.0051 |
| GO:0070970 | interleukin-2 secretion | 3 | 1 | 0.00586 |
| GO:0044710 | single-organism metabolic process | 3344 | 16 | 0.006736 |
| GO:0034418 | urate biosynthetic process | 4 | 1 | 0.006956 |
| GO:0009165 | nucleotide biosynthetic process | 174 | 3 | 0.006981 |
| GO:1901293 | nucleoside phosphate biosynthetic process | 177 | 3 | 0.007236 |
| GO:0055086 | nucleobase-containing small molecule metabolic process | 334 | 4 | 0.007243 |
| GO:0006139 | nucleobase-containing compound metabolic process | 3721 | 17 | 0.007382 |
| GO:0006996 | organelle organization | 1805 | 14 | 0.007719 |
| GO:0043578 | nuclear matrix organization | 6 | 2 | 0.008205 |
| GO:0090286 | cytoskeletal anchoring at nuclear membrane | 6 | 2 | 0.008205 |
| GO:0090292 | nuclear matrix anchoring at nuclear membrane | 6 | 2 | 0.008205 |
| GO:0009156 | ribonucleoside monophosphate biosynthetic process | 65 | 2 | 0.008709 |
| GO:0009124 | nucleoside monophosphate biosynthetic process | 68 | 2 | 0.009309 |
| GO:0046483 | heterocycle metabolic process | 3835 | 17 | 0.009315 |
| GO:1901566 | organonitrogen compound biosynthetic process | 384 | 4 | 0.009477 |
| GO:0042451 | purine nucleoside biosynthetic process | 64 | 2 | 0.009878 |
| GO:0046129 | purine ribonucleoside biosynthetic process | 64 | 2 | 0.009878 |
| GO:0046102 | inosine metabolic process | 6 | 1 | 0.009892 |
| GO:0051457 | maintenance of protein location in nucleus | 8 | 2 | 0.010034 |
| GO:0072526 | pyridine-containing compound catabolic process | 7 | 1 | 0.011374 |
| GO:0019509 | L-methionine biosynthetic process from methylthioadenosine | 7 | 1 | 0.011423 |
| GO:0043102 | amino acid salvage | 7 | 1 | 0.011423 |
| GO:0071265 | L-methionine biosynthetic process | 7 | 1 | 0.011423 |
| GO:0071267 | L-methionine salvage | 7 | 1 | 0.011423 |
| GO:0072595 | maintenance of protein localization in organelle | 12 | 2 | 0.011557 |
| GO:0010138 | pyrimidine ribonucleotide salvage | 7 | 1 | 0.011903 |
| GO:0032262 | pyrimidine nucleotide salvage | 7 | 1 | 0.011903 |
| GO:0044206 | UMP salvage | 7 | 1 | 0.011903 |
| GO:0034641 | cellular nitrogen compound metabolic process | 3982 | 17 | 0.012692 |
| GO:0046434 | organophosphate catabolic process | 77 | 2 | 0.013264 |
| GO:0072521 | purine-containing compound metabolic process | 224 | 3 | 0.013321 |
| GO:0039694 | viral RNA genome replication | 17 | 2 | 0.013364 |
| GO:0039703 | RNA replication | 17 | 2 | 0.013364 |
| GO:0050793 | regulation of developmental process | 1298 | 9 | 0.013694 |
| GO:0006725 | cellular aromatic compound metabolic process | 3918 | 17 | 0.01372 |
| GO:0006166 | purine ribonucleoside salvage | 8 | 1 | 0.014077 |
| GO:0051239 | regulation of multicellular organismal process | 1323 | 9 | 0.014522 |
| GO:0043097 | pyrimidine nucleoside salvage | 9 | 1 | 0.015001 |
| GO:0044033 | multi-organism metabolic process | 20 | 2 | 0.015186 |
| GO:0009161 | ribonucleoside monophosphate metabolic process | 89 | 2 | 0.015414 |
| GO:0009123 | nucleoside monophosphate metabolic process | 93 | 2 | 0.016462 |
| GO:0008655 | pyrimidine-containing compound salvage | 10 | 1 | 0.016546 |
| GO:0019079 | viral genome replication | 26 | 2 | 0.017184 |
| GO:0042742 | defense response to bacterium | 184 | 3 | 0.01788 |
| GO:0050663 | cytokine secretion | 8 | 1 | 0.017916 |
| GO:0009435 | NAD biosynthetic process | 10 | 1 | 0.018801 |
| GO:0046637 | regulation of alpha-beta T cell differentiation | 9 | 1 | 0.018962 |
| GO:0046638 | positive regulation of alpha-beta T cell differentiation | 9 | 1 | 0.018962 |
| GO:0042102 | positive regulation of T cell proliferation | 11 | 1 | 0.019975 |
| GO:0048513 | organ development | 1015 | 8 | 0.020203 |
| GO:0042692 | muscle cell differentiation | 38 | 2 | 0.021065 |
| GO:0009617 | response to bacterium | 200 | 3 | 0.021189 |
| GO:0046634 | regulation of alpha-beta T cell activation | 10 | 1 | 0.021515 |
| GO:0046635 | positive regulation of alpha-beta T cell activation | 10 | 1 | 0.021515 |
| GO:0046130 | purine ribonucleoside catabolic process | 13 | 1 | 0.021648 |
| GO:0032946 | positive regulation of mononuclear cell proliferation | 12 | 1 | 0.022285 |
| GO:0050671 | positive regulation of lymphocyte proliferation | 12 | 1 | 0.022285 |
| GO:0070665 | positive regulation of leukocyte proliferation | 12 | 1 | 0.022285 |
| GO:1901360 | organic cyclic compound metabolic process | 4064 | 17 | 0.022525 |
| GO:0006152 | purine nucleoside catabolic process | 14 | 1 | 0.023182 |
| GO:0045582 | positive regulation of T cell differentiation | 11 | 1 | 0.024009 |
| GO:0006807 | nitrogen compound metabolic process | 4327 | 17 | 0.024755 |
| GO:0019359 | nicotinamide nucleotide biosynthetic process | 12 | 1 | 0.024984 |
| GO:0006716 | juvenile hormone metabolic process | 17 | 1 | 0.026375 |
| GO:0006718 | juvenile hormone biosynthetic process | 17 | 1 | 0.026375 |
| GO:0006555 | methionine metabolic process | 15 | 1 | 0.026685 |
| GO:0009086 | methionine biosynthetic process | 15 | 1 | 0.026685 |
| GO:1901137 | carbohydrate derivative biosynthetic process | 287 | 3 | 0.026694 |
| GO:0009117 | nucleotide metabolic process | 268 | 3 | 0.026873 |
| GO:0019363 | pyridine nucleotide biosynthetic process | 13 | 1 | 0.02689 |
| GO:0006753 | nucleoside phosphate metabolic process | 271 | 3 | 0.027424 |
| GO:0046415 | urate metabolic process | 16 | 1 | 0.027922 |
| GO:0006195 | purine nucleotide catabolic process | 16 | 1 | 0.028231 |
| GO:0045621 | positive regulation of lymphocyte differentiation | 14 | 1 | 0.030129 |
| GO:0045580 | regulation of T cell differentiation | 14 | 1 | 0.030562 |
| GO:0042129 | regulation of T cell proliferation | 16 | 1 | 0.030629 |
| GO:0019674 | NAD metabolic process | 15 | 1 | 0.030651 |
| GO:0043173 | nucleotide salvage | 17 | 1 | 0.030816 |
| GO:0042454 | ribonucleoside catabolic process | 19 | 1 | 0.031002 |
| GO:0006222 | UMP biosynthetic process | 11 | 1 | 0.031624 |
| GO:0009173 | pyrimidine ribonucleoside monophosphate metabolic process | 11 | 1 | 0.031624 |
| GO:0009174 | pyrimidine ribonucleoside monophosphate biosynthetic process | 11 | 1 | 0.031624 |
| GO:0046049 | UMP metabolic process | 11 | 1 | 0.031624 |
| GO:0072525 | pyridine-containing compound biosynthetic process | 17 | 1 | 0.033746 |
| GO:0015771 | trehalose transport | 18 | 1 | 0.034585 |
| GO:0009129 | pyrimidine nucleoside monophosphate metabolic process | 13 | 1 | 0.03489 |
| GO:0009130 | pyrimidine nucleoside monophosphate biosynthetic process | 13 | 1 | 0.03489 |
| GO:1901135 | carbohydrate derivative metabolic process | 572 | 4 | 0.034891 |
| GO:0050870 | positive regulation of T cell activation | 16 | 1 | 0.035075 |
| GO:1903039 | positive regulation of leukocyte cell-cell adhesion | 16 | 1 | 0.035075 |
| GO:0032944 | regulation of mononuclear cell proliferation | 19 | 1 | 0.035945 |
| GO:0050670 | regulation of lymphocyte proliferation | 19 | 1 | 0.035945 |
| GO:0070663 | regulation of leukocyte proliferation | 19 | 1 | 0.035945 |
| GO:0016106 | sesquiterpenoid biosynthetic process | 19 | 1 | 0.036261 |
| GO:0090407 | organophosphate biosynthetic process | 328 | 3 | 0.036428 |
| GO:0034112 | positive regulation of homotypic cell-cell adhesion | 17 | 1 | 0.036588 |
| GO:0045619 | regulation of lymphocyte differentiation | 17 | 1 | 0.036642 |
| GO:0009260 | ribonucleotide biosynthetic process | 128 | 2 | 0.036923 |
| GO:0046390 | ribose phosphate biosynthetic process | 129 | 2 | 0.037301 |
| GO:0044275 | cellular carbohydrate catabolic process | 15 | 1 | 0.038472 |
| GO:0015766 | disaccharide transport | 20 | 1 | 0.039173 |
| GO:0015772 | oligosaccharide transport | 20 | 1 | 0.039173 |
| GO:0000097 | sulfur amino acid biosynthetic process | 22 | 1 | 0.040354 |
| GO:0009164 | nucleoside catabolic process | 24 | 1 | 0.040657 |
| GO:0006714 | sesquiterpenoid metabolic process | 22 | 1 | 0.040793 |
| GO:0015985 | energy coupled proton transport, down electrochemical gradient | 24 | 1 | 0.040882 |
| GO:0015986 | ATP synthesis coupled proton transport | 24 | 1 | 0.040882 |
| GO:0009220 | pyrimidine ribonucleotide biosynthetic process | 16 | 1 | 0.042117 |
| GO:0046132 | pyrimidine ribonucleoside biosynthetic process | 16 | 1 | 0.042117 |
| GO:0009067 | aspartate family amino acid biosynthetic process | 25 | 1 | 0.044053 |
| GO:0009218 | pyrimidine ribonucleotide metabolic process | 17 | 1 | 0.044172 |
| GO:0098542 | defense response to other organism | 241 | 3 | 0.045006 |
| GO:0046134 | pyrimidine nucleoside biosynthetic process | 18 | 1 | 0.045123 |
| GO:0044711 | single-organism biosynthetic process | 903 | 5 | 0.045738 |
| GO:0016114 | terpenoid biosynthetic process | 25 | 1 | 0.04727 |
| GO:0006754 | ATP biosynthetic process | 29 | 1 | 0.048628 |
| GO:0046131 | pyrimidine ribonucleoside metabolic process | 20 | 1 | 0.048662 |
| GO:0044707 | single-multicellular organism process | 2424 | 12 | 0.049006 |
| GO:0032507 | maintenance of protein location in cell | 63 | 2 | 0.049147 |
| GO:0006997 | nucleus organization | 55 | 2 | 0.049373 |

Table S10. Cellular component GO terms enriched in lure-mated females when compared with virgin females

| **category** | **term** | **Numbers in category** | **Numbers differentially expressed** | **P value** |
| --- | --- | --- | --- | --- |
| GO:0005863 | striated muscle myosin thick filament | 17 | 8 | 0 |
| GO:0030017 | sarcomere | 40 | 10 | 0 |
| GO:0005859 | muscle myosin complex | 23 | 8 | 4.85E-11 |
| GO:0031674 | I band | 23 | 8 | 5.07E-11 |
| GO:0032982 | myosin filament | 30 | 8 | 5.75E-11 |
| GO:0036379 | myofilament | 26 | 8 | 7.57E-11 |
| GO:0000794 | condensed nuclear chromosome | 31 | 8 | 1.47E-10 |
| GO:0016460 | myosin II complex | 28 | 8 | 2.15E-10 |
| GO:0000793 | condensed chromosome | 39 | 8 | 4.00E-10 |
| GO:0044449 | contractile fiber part | 122 | 12 | 3.58E-09 |
| GO:0000228 | nuclear chromosome | 70 | 8 | 4.19E-09 |
| GO:0016459 | myosin complex | 70 | 8 | 1.68E-08 |
| GO:0030018 | Z disc | 75 | 8 | 6.20E-08 |
| GO:0005694 | chromosome | 241 | 9 | 2.00E-06 |
| GO:0005875 | microtubule associated complex | 344 | 9 | 7.40E-06 |
| GO:0042600 | chorion | 8 | 2 | 0.000109 |
| GO:0030312 | external encapsulating structure | 15 | 2 | 0.00038 |
| GO:0000786 | nucleosome | 17 | 2 | 0.000994 |
| GO:1990104 | DNA bending complex | 17 | 2 | 0.000994 |
| GO:0044430 | cytoskeletal part | 865 | 10 | 0.001228 |
| GO:0043228 | non-membrane-bounded organelle | 1585 | 14 | 0.001363 |
| GO:0043232 | intracellular non-membrane-bounded organelle | 1585 | 14 | 0.001363 |
| GO:0044815 | DNA packaging complex | 22 | 2 | 0.001958 |
| GO:0032993 | protein-DNA complex | 33 | 2 | 0.002687 |
| GO:0045098 | type III intermediate filament | 3 | 1 | 0.004886 |
| GO:0019028 | viral capsid | 17 | 2 | 0.004985 |
| GO:0034993 | SUN-KASH complex | 6 | 2 | 0.008205 |
| GO:0005640 | nuclear outer membrane | 8 | 2 | 0.00945 |
| GO:0043234 | protein complex | 2695 | 15 | 0.01283 |
| GO:0044423 | virion part | 54 | 2 | 0.013285 |
| GO:0030430 | host cell cytoplasm | 43 | 2 | 0.014011 |
| GO:0033646 | host intracellular part | 56 | 2 | 0.018232 |
| GO:0045261 | proton-transporting ATP synthase complex, catalytic core F(1) | 12 | 1 | 0.020331 |
| GO:0033643 | host cell part | 65 | 2 | 0.025102 |
| GO:0044217 | other organism part | 65 | 2 | 0.025102 |
| GO:0044428 | nuclear part | 1753 | 11 | 0.036276 |
| GO:0019867 | outer membrane | 73 | 2 | 0.036443 |
| GO:0031968 | organelle outer membrane | 73 | 2 | 0.036443 |

Table S11. Molecular function GO terms enriched in lure-mated females when compared with virgin females

| **category** | **term** | **Numbers in category** | **Numbers differentially expressed** | **P value** |
| --- | --- | --- | --- | --- |
| GO:0008307 | structural constituent of muscle | 40 | 8 | 1.57E-08 |
| GO:0003779 | actin binding | 195 | 10 | 1.39E-07 |
| GO:0005198 | structural molecule activity | 520 | 10 | 4.41E-06 |
| GO:0016763 | transferase activity, transferring pentosyl groups | 27 | 3 | 1.73E-05 |
| GO:0008092 | cytoskeletal protein binding | 395 | 10 | 1.91E-05 |
| GO:0003796 | lysozyme activity | 12 | 2 | 0.000157 |
| GO:0004568 | chitinase activity | 29 | 2 | 0.001118 |
| GO:0017061 | S-methyl-5-thioadenosine phosphorylase activity | 3 | 1 | 0.004963 |
| GO:0002060 | purine nucleobase binding | 3 | 1 | 0.005099 |
| GO:0004731 | purine-nucleoside phosphorylase activity | 3 | 1 | 0.005099 |
| GO:0042301 | phosphate ion binding | 4 | 1 | 0.006673 |
| GO:0002054 | nucleobase binding | 4 | 1 | 0.007473 |
| GO:0004850 | uridine phosphorylase activity | 5 | 1 | 0.008001 |
| GO:0016757 | transferase activity, transferring glycosyl groups | 202 | 3 | 0.008232 |
| GO:0005521 | lamin binding | 7 | 2 | 0.008699 |
| GO:0003968 | RNA-directed RNA polymerase activity | 20 | 2 | 0.017921 |
| GO:0047886 | farnesol dehydrogenase activity | 15 | 1 | 0.023304 |
| GO:0046933 | proton-transporting ATP synthase activity, rotational mechanism | 14 | 1 | 0.024246 |
| GO:0003724 | RNA helicase activity | 63 | 2 | 0.02842 |
| GO:0051015 | actin filament binding | 47 | 2 | 0.03184 |
| GO:0004553 | hydrolase activity, hydrolyzing O-glycosyl compounds | 147 | 2 | 0.032244 |
| GO:0015151 | alpha-glucoside transmembrane transporter activity | 18 | 1 | 0.034585 |
| GO:0015574 | trehalose transmembrane transporter activity | 18 | 1 | 0.034585 |
| GO:0042947 | glucoside transmembrane transporter activity | 18 | 1 | 0.034585 |
| GO:0034062 | RNA polymerase activity | 63 | 2 | 0.035873 |
| GO:0015154 | disaccharide transmembrane transporter activity | 20 | 1 | 0.039173 |
| GO:0015157 | oligosaccharide transmembrane transporter activity | 20 | 1 | 0.039173 |
| GO:0016798 | hydrolase activity, acting on glycosyl bonds | 162 | 2 | 0.041521 |

Table S12. GO terms down regulated in lure-mated females when compared with virgin females

| **category** | **term** | **Numbers in category** | **Numbers differentially expressed** | **P value** |
| --- | --- | --- | --- | --- |
| Biological processes | | | | |
| GO:0031323 | regulation of cellular metabolic process | 2804 | 1 | 0.000548 |
| GO:0060255 | regulation of macromolecule metabolic process | 2773 | 1 | 0.000557 |
| GO:0080090 | regulation of primary metabolic process | 2660 | 1 | 0.00085 |
| GO:0007165 | signal transduction | 1883 | 0 | 0.00187 |
| GO:0048519 | negative regulation of biological process | 2116 | 1 | 0.002111 |
| GO:0019222 | regulation of metabolic process | 3216 | 3 | 0.002131 |
| GO:0048523 | negative regulation of cellular process | 1911 | 1 | 0.004281 |
| GO:0010468 | regulation of gene expression | 2204 | 1 | 0.006921 |
| GO:0009653 | anatomical structure morphogenesis | 1407 | 0 | 0.008501 |
| GO:0051171 | regulation of nitrogen compound metabolic process | 2127 | 1 | 0.008567 |
| GO:0009889 | regulation of biosynthetic process | 2100 | 1 | 0.009541 |
| GO:0031326 | regulation of cellular biosynthetic process | 2089 | 1 | 0.009869 |
| GO:0051128 | regulation of cellular component organization | 1252 | 0 | 0.011303 |
| GO:0019219 | regulation of nucleobase-containing compound metabolic process | 2049 | 1 | 0.011459 |
| GO:0010556 | regulation of macromolecule biosynthetic process | 1990 | 1 | 0.012866 |
| GO:2000112 | regulation of cellular macromolecule biosynthetic process | 1969 | 1 | 0.013722 |
| GO:0051252 | regulation of RNA metabolic process | 1866 | 1 | 0.018578 |
| GO:0031325 | positive regulation of cellular metabolic process | 1263 | 0 | 0.021833 |
| GO:0009892 | negative regulation of metabolic process | 1126 | 0 | 0.021975 |
| GO:2001141 | regulation of RNA biosynthetic process | 1801 | 1 | 0.022084 |
| GO:0006355 | regulation of transcription, DNA-templated | 1792 | 1 | 0.022661 |
| GO:0010604 | positive regulation of macromolecule metabolic process | 1150 | 0 | 0.027814 |
| GO:0007166 | cell surface receptor signaling pathway | 1113 | 0 | 0.028326 |
| GO:0048522 | positive regulation of cellular process | 2084 | 2 | 0.028391 |
| GO:0006928 | cellular component movement | 962 | 0 | 0.031253 |
| GO:0010605 | negative regulation of macromolecule metabolic process | 989 | 0 | 0.037512 |
| GO:0051246 | regulation of protein metabolic process | 807 | 0 | 0.037531 |
| GO:0031324 | negative regulation of cellular metabolic process | 933 | 0 | 0.038388 |
| GO:0044267 | cellular protein metabolic process | 1818 | 2 | 0.040692 |
| GO:0019538 | protein metabolic process | 2370 | 3 | 0.041767 |
| GO:0032268 | regulation of cellular protein metabolic process | 756 | 0 | 0.044279 |
| GO:0032879 | regulation of localization | 858 | 0 | 0.0496 |
| Cellular component | | | | |
| GO:0016020 | membrane | 3753 | 4 | 0.006526 |
| GO:0043227 | membrane-bounded organelle | 5567 | 8 | 0.006829 |
| GO:0043231 | intracellular membrane-bounded organelle | 5261 | 8 | 0.016174 |
| GO:0044425 | membrane part | 3809 | 5 | 0.038189 |
| GO:0005886 | plasma membrane | 1655 | 1 | 0.048329 |
| Molecular function | | | | |
| GO:0043169 | cation binding | 3480 | 3 | 0.004204 |
| GO:0046872 | metal ion binding | 3420 | 3 | 0.004734 |
| GO:0043167 | ion binding | 4881 | 8 | 0.019285 |

Table S13. Gene transcripts up-regulated in lure-mated females when compared with control-mated females

| **Gene ID** | **P** | **FDR** | **Functions** | **Reference sp** |
| --- | --- | --- | --- | --- |
| TRINITY_DN36607_c0_g1 | 3.24E-27 | 2.73E-22 | unknown |  |
| TRINITY_DN35577_c0_g1 | 7.10E-24 | 2.99E-19 | unknown | c capitata |
| TRINITY_DN18198_c0_g2 | 6.79E-21 | 1.91E-16 | Membrane transporter | B dorsalis |
| TRINITY_DN31168_c0_g1 | 2.07E-20 | 4.37E-16 | unknown |  |
| TRINITY_DN36898_c0_g1 | 2.71E-20 | 4.56E-16 | unknown | B dorsalis |
| TRINITY_DN26791_c0_g1 | 1.80E-18 | 2.16E-14 | unknown | c capitata |
| TRINITY_DN23355_c0_g1 | 1.88E-17 | 1.76E-13 | Nucleosome metabolic processes | B dorsalis; D melano |
| TRINITY_DN39613_c0_g1 | 3.84E-17 | 3.21E-13 | unknown | B dorsalis |
| TRINITY_DN38387_c0_g1 | 4.19E-17 | 3.21E-13 | Immune response | B dorsalis; D melano |
| TRINITY_DN37197_c0_g1 | 5.34E-17 | 3.75E-13 | unknown |  |
| TRINITY_DN23140_c0_g1 | 8.24E-17 | 5.34E-13 | unknown |  |
| TRINITY_DN31642_c0_g1 | 2.79E-16 | 1.57E-12 | unknown | B dorsalis |
| TRINITY_DN32536_c0_g1 | 3.97E-16 | 2.09E-12 | unknown | B dorsalis |
| TRINITY_DN31576_c0_g1 | 8.78E-16 | 4.35E-12 | unknown |  |
| TRINITY_DN30147_c0_g1 | 1.38E-15 | 6.44E-12 | unknown | B dorsalis |
| TRINITY_DN32691_c0_g1 | 1.00E-14 | 4.21E-11 | unknown | B dorsalis |
| TRINITY_DN31574_c0_g1 | 1.91E-14 | 7.65E-11 | unknown |  |
| TRINITY_DN33970_c0_g1 | 1.12E-13 | 4.29E-10 | unknown |  |
| TRINITY_DN26915_c0_g1 | 7.10E-13 | 2.60E-09 | unknown |  |
| TRINITY_DN64100_c0_g1 | 1.51E-12 | 5.14E-09 | ATP synthase complex | D melano; |
| TRINITY_DN17139_c0_g1 | 1.52E-12 | 5.14E-09 | unknown |  |
| TRINITY_DN33239_c0_g1 | 6.09E-12 | 1.98E-08 | unknown | B dorsalis |
| TRINITY_DN40180_c0_g5 | 1.05E-11 | 3.27E-08 | Sarcotoxin; Cecropin | Sarcophaga peregrina; Hyalophora cecropia |
| TRINITY_DN35504_c0_g1 | 2.06E-11 | 6.21E-08 | unknown | B dorsalis |
| TRINITY_DN18460_c0_g1 | 3.82E-11 | 1.11E-07 | unknown | B dorsalis |
| TRINITY_DN37787_c0_g1 | 6.44E-11 | 1.81E-07 | unknown | B dorsalis |
| TRINITY_DN66196_c0_g1 | 7.36E-11 | 1.94E-07 | unknown |  |
| TRINITY_DN19930_c0_g3 | 7.37E-11 | 1.94E-07 | Oogenesis/ovarian cell development | D melano; B dorsalis |
| TRINITY_DN21170_c0_g1 | 8.19E-11 | 2.09E-07 | unknown |  |
| TRINITY_DN32162_c0_g1 | 2.08E-10 | 5.16E-07 | Response to drug/metabolic processes | B dorsalis; Human |
| TRINITY_DN22480_c0_g1 | 4.55E-10 | 1.10E-06 | unknown | B dorsalis |
| TRINITY_DN28099_c0_g1 | 6.58E-10 | 1.54E-06 | unknown | B dorsalis |
| TRINITY_DN39825_c0_g2 | 7.68E-10 | 1.70E-06 | Cytosol aminopeptidase | c capitata; |
| TRINITY_DN33640_c0_g1 | 7.88E-10 | 1.70E-06 | unknown |  |
| TRINITY_DN19937_c0_g1 | 1.06E-09 | 2.24E-06 | unknown | B dorsalis |
| TRINITY_DN72019_c0_g1 | 1.62E-09 | 3.24E-06 | unknown | B dorsalis |
| TRINITY_DN19930_c0_g2 | 1.96E-09 | 3.75E-06 | DNA binding/metal ion binding | D melano; B dorsalis |
| TRINITY_DN37815_c0_g2 | 2.73E-09 | 5.11E-06 | unknown | B dorsalis |
| TRINITY_DN24697_c0_g1 | 2.79E-09 | 5.12E-06 | unknown | B dorsalis |
| TRINITY_DN25193_c0_g1 | 3.39E-09 | 6.07E-06 | DNA polymerase activity | D melano; C capitata |
| TRINITY_DN18872_c0_g1 | 3.92E-09 | 6.88E-06 | unknown | c capitata |
| TRINITY_DN2552_c0_g1 | 9.11E-09 | 1.45E-05 | unknown | B dorsalis |
| TRINITY_DN22584_c0_g1 | 9.11E-09 | 1.45E-05 | unknown | B dorsalis |
| TRINITY_DN36373_c0_g1 | 1.05E-08 | 1.64E-05 | Reverse transcriptase | D melano |
| TRINITY_DN6705_c0_g1 | 1.73E-08 | 2.60E-05 | Spermatogenesis | C capitata |
| TRINITY_DN36426_c0_g1 | 1.76E-08 | 2.60E-05 | unknown | Danaus plexippus |
| TRINITY_DN17803_c0_g1 | 2.20E-08 | 3.20E-05 | unknown |  |
| TRINITY_DN62012_c0_g1 | 2.32E-08 | 3.31E-05 | unknown |  |
| TRINITY_DN30781_c0_g1 | 2.75E-08 | 3.80E-05 | unknown | B dorsalis |
| TRINITY_DN54510_c0_g1 | 4.20E-08 | 5.71E-05 | unknown |  |
| TRINITY_DN34653_c0_g1 | 4.28E-08 | 5.73E-05 | unknown |  |
| TRINITY_DN17803_c0_g2 | 5.90E-08 | 7.65E-05 | unknown | B dorsalis; C capitata |
| TRINITY_DN28122_c0_g1 | 7.88E-08 | 0.000101 | linker histone H1 and H5 | B dorsalis; Chironomous thummi thummi |
| TRINITY_DN34372_c0_g1 | 8.83E-08 | 0.000111 | unknown | B dorsalis |
| TRINITY_DN11354_c0_g2 | 9.37E-08 | 0.000116 | unknown |  |
| TRINITY_DN9774_c0_g1 | 1.33E-07 | 0.000159 | unknown | B dorsalis |
| TRINITY_DN42232_c0_g1 | 2.24E-07 | 0.000263 | short chain dehydrogenase; Enoyl-(Acyl carrier protein) reductase; Farnesol dehydrogenase | B dorsalis, A. agepti |
| TRINITY_DN70106_c0_g1 | 2.96E-07 | 0.000342 | unknown | C capitata |
| TRINITY_DN29460_c0_g1 | 3.12E-07 | 0.000355 | unknown | B dorsalis |
| TRINITY_DN9593_c0_g1 | 3.77E-07 | 0.000418 | unknown | B dorsalis |
| TRINITY_DN34179_c0_g1 | 5.93E-07 | 0.000641 | Sperm outer dense fibre protein | B dorsalis; Danio rerio |
| TRINITY_DN33196_c0_g1 | 6.33E-07 | 0.000667 | unknown | C capitata |
| TRINITY_DN47898_c0_g1 | 6.41E-07 | 0.000667 | unknown | B dorsalis |
| TRINITY_DN24567_c0_g1 | 6.60E-07 | 0.000677 | unknown | B dorsalis |
| TRINITY_DN6076_c0_g1 | 6.66E-07 | 0.000677 | unknown |  |
| TRINITY_DN30958_c0_g1 | 7.15E-07 | 0.000712 | unknown | B dorsalis |
| TRINITY_DN1536_c0_g1 | 7.18E-07 | 0.000712 | Testis expressed | B dorsalis; Mouse |
| TRINITY_DN15832_c0_g2 | 7.72E-07 | 0.000757 | Lectin C-type domain | B dorsalis; Ovis (sheep) |
| TRINITY_DN57008_c0_g1 | 8.68E-07 | 0.000841 | unknown | B dorsalis |
| TRINITY_DN73333_c0_g1 | 9.78E-07 | 0.000926 | unknown | B dorsalis |

Table S14. Gene transcripts down-regulated in lure-mated females compared with control-mated females

| **Gene ID** | **P** | **FDR** | **Functions** | **Reference sp** |
| --- | --- | --- | --- | --- |
| TRINITY_DN13697_c0_g1 | 1.12E-19 | 1.57E-15 | unknown |  |
| TRINITY_DN8949_c0_g1 | 2.13E-18 | 2.24E-14 | unknown |  |
| TRINITY_DN41896_c0_g2 | 2.16E-16 | 1.30E-12 | unknown | Reductase like protein |
| TRINITY_DN25186_c0_g1 | 4.16E-15 | 1.85E-11 | binding nuclear protein | D melano; A mellifera |
| TRINITY_DN20065_c0_g2 | 7.79E-10 | 1.70E-06 | unknown |  |
| TRINITY_DN27193_c0_g2 | 1.49E-09 | 3.07E-06 | unknown |  |
| TRINITY_DN15988_c0_g3 | 1.83E-09 | 3.60E-06 | Carboxylate reductase activity | B dorsalis; |
| TRINITY_DN21918_c0_g1 | 4.31E-09 | 7.42E-06 | unknown | B dorsalis |
| TRINITY_DN32739_c0_g1 | 4.41E-09 | 7.43E-06 | unknown |  |
| TRINITY_DN35011_c0_g3 | 7.68E-09 | 1.27E-05 | nuclear transport factor | D melano; C capitata |
| TRINITY_DN26719_c0_g1 | 1.16E-08 | 1.79E-05 | unknown |  |
| TRINITY_DN42003_c0_g1 | 2.50E-08 | 3.51E-05 | unknown |  |
| TRINITY_DN1323_c0_g1 | 4.93E-08 | 6.49E-05 | unknown |  |
| TRINITY_DN37965_c0_g1 | 1.15E-07 | 0.00014 | Ribosomal protein | B dorsalis; D melano |
| TRINITY_DN28376_c0_g1 | 1.34E-07 | 0.000159 | Transposable element Tc1 transposase | B dorsalis; C elegans |
| TRINITY_DN69034_c0_g1 | 3.70E-07 | 0.000416 | Occludin homology domain | B dorsalis; D melano |
| TRINITY_DN39130_c0_g1 | 4.68E-07 | 0.000512 | Obp56A - Odorant binding protein | B dorsalis; D mealno |
| TRINITY_DN47468_c0_g1 | 6.13E-07 | 0.000654 | unknown |  |
| TRINITY_DN33113_c0_g1 | 9.34E-07 | 0.000895 | unknown |  |

Table S15. Biological processes GO term up-regulated in lure-mated females when compared with control-mated females

| **GO Category** | **GO Function** | **Numbers in category** | **Number differentially expressed** | **P value** |
| --- | --- | --- | --- | --- |
| GO:0043101 | purine-containing compound salvage | 15 | 2 | 0.00013 |
| GO:0046128 | purine ribonucleoside metabolic process | 120 | 3 | 0.000338 |
| GO:0042278 | purine nucleoside metabolic process | 126 | 3 | 0.000389 |
| GO:0072522 | purine-containing compound biosynthetic process | 128 | 3 | 0.000416 |
| GO:0009119 | ribonucleoside metabolic process | 142 | 3 | 0.000558 |
| GO:0009116 | nucleoside metabolic process | 156 | 3 | 0.00073 |
| GO:0043094 | cellular metabolic compound salvage | 38 | 2 | 0.000847 |
| GO:1901657 | glycosyl compound metabolic process | 170 | 3 | 0.000932 |
| GO:0019523 | L-idonate metabolic process | 1 | 1 | 0.001442 |
| GO:0046176 | aldonic acid catabolic process | 1 | 1 | 0.001442 |
| GO:0046183 | L-idonate catabolic process | 1 | 1 | 0.001442 |
| GO:0072521 | purine-containing compound metabolic process | 224 | 3 | 0.00214 |
| GO:0006738 | nicotinamide riboside catabolic process | 2 | 1 | 0.002257 |
| GO:0046495 | nicotinamide riboside metabolic process | 2 | 1 | 0.002257 |
| GO:0070637 | pyridine nucleoside metabolic process | 2 | 1 | 0.002257 |
| GO:0070638 | pyridine nucleoside catabolic process | 2 | 1 | 0.002257 |
| GO:0042451 | purine nucleoside biosynthetic process | 64 | 2 | 0.002459 |
| GO:0046129 | purine ribonucleoside biosynthetic process | 64 | 2 | 0.002459 |
| GO:0019520 | aldonic acid metabolic process | 2 | 1 | 0.002573 |
| GO:0006148 | inosine catabolic process | 3 | 1 | 0.003383 |
| GO:0034356 | NAD biosynthesis via nicotinamide riboside salvage pathway | 3 | 1 | 0.003383 |
| GO:0070970 | interleukin-2 secretion | 3 | 1 | 0.003422 |
| GO:0042455 | ribonucleoside biosynthetic process | 83 | 2 | 0.004144 |
| GO:0009163 | nucleoside biosynthetic process | 85 | 2 | 0.004334 |
| GO:0034418 | urate biosynthetic process | 4 | 1 | 0.004508 |
| GO:1901659 | glycosyl compound biosynthetic process | 87 | 2 | 0.004528 |
| GO:0055086 | nucleobase-containing small molecule metabolic process | 334 | 3 | 0.006607 |
| GO:0046102 | inosine metabolic process | 6 | 1 | 0.006752 |
| GO:0019509 | L-methionine biosynthetic process from methylthioadenosine | 7 | 1 | 0.00787 |
| GO:0043102 | amino acid salvage | 7 | 1 | 0.00787 |
| GO:0071265 | L-methionine biosynthetic process | 7 | 1 | 0.00787 |
| GO:0071267 | L-methionine salvage | 7 | 1 | 0.00787 |
| GO:0072526 | pyridine-containing compound catabolic process | 7 | 1 | 0.007872 |
| GO:0006166 | purine ribonucleoside salvage | 8 | 1 | 0.009072 |
| GO:1901566 | organonitrogen compound biosynthetic process | 384 | 3 | 0.009443 |
| GO:0050663 | cytokine secretion | 8 | 1 | 0.009787 |
| GO:0046637 | regulation of alpha-beta T cell differentiation | 9 | 1 | 0.010834 |
| GO:0046638 | positive regulation of alpha-beta T cell differentiation | 9 | 1 | 0.010834 |
| GO:0019835 | cytolysis | 10 | 1 | 0.011225 |
| GO:0009435 | NAD biosynthetic process | 10 | 1 | 0.011506 |
| GO:0046634 | regulation of alpha-beta T cell activation | 10 | 1 | 0.012196 |
| GO:0046635 | positive regulation of alpha-beta T cell activation | 10 | 1 | 0.012196 |
| GO:0042102 | positive regulation of T cell proliferation | 11 | 1 | 0.012416 |
| GO:0045582 | positive regulation of T cell differentiation | 11 | 1 | 0.013405 |
| GO:0032946 | positive regulation of mononuclear cell proliferation | 12 | 1 | 0.013587 |
| GO:0050671 | positive regulation of lymphocyte proliferation | 12 | 1 | 0.013587 |
| GO:0070665 | positive regulation of leukocyte proliferation | 12 | 1 | 0.013587 |
| GO:0019359 | nicotinamide nucleotide biosynthetic process | 12 | 1 | 0.014249 |
| GO:0046130 | purine ribonucleoside catabolic process | 13 | 1 | 0.014696 |
| GO:0019363 | pyridine nucleotide biosynthetic process | 13 | 1 | 0.015364 |
| GO:0006152 | purine nucleoside catabolic process | 14 | 1 | 0.015809 |
| GO:0006163 | purine nucleotide metabolic process | 167 | 2 | 0.016299 |
| GO:0045621 | positive regulation of lymphocyte differentiation | 14 | 1 | 0.016948 |
| GO:0006555 | methionine metabolic process | 15 | 1 | 0.016995 |
| GO:0009086 | methionine biosynthetic process | 15 | 1 | 0.016995 |
| GO:0045580 | regulation of T cell differentiation | 14 | 1 | 0.017002 |
| GO:0009165 | nucleotide biosynthetic process | 174 | 2 | 0.01738 |
| GO:0019674 | NAD metabolic process | 15 | 1 | 0.017677 |
| GO:1901293 | nucleoside phosphate biosynthetic process | 177 | 2 | 0.017926 |
| GO:0006195 | purine nucleotide catabolic process | 16 | 1 | 0.018098 |
| GO:0046415 | urate metabolic process | 16 | 1 | 0.018199 |
| GO:0042129 | regulation of T cell proliferation | 16 | 1 | 0.018278 |
| GO:0044275 | cellular carbohydrate catabolic process | 15 | 1 | 0.018456 |
| GO:0044711 | single-organism biosynthetic process | 903 | 4 | 0.018647 |
| GO:0006716 | juvenile hormone metabolic process | 17 | 1 | 0.019012 |
| GO:0006718 | juvenile hormone biosynthetic process | 17 | 1 | 0.019012 |
| GO:0043174 | nucleoside salvage | 17 | 1 | 0.019154 |
| GO:0050870 | positive regulation of T cell activation | 16 | 1 | 0.019278 |
| GO:1903039 | positive regulation of leukocyte cell-cell adhesion | 16 | 1 | 0.019278 |
| GO:0072525 | pyridine-containing compound biosynthetic process | 17 | 1 | 0.019857 |
| GO:0042742 | defense response to bacterium | 184 | 2 | 0.020102 |
| GO:0034112 | positive regulation of homotypic cell-cell adhesion | 17 | 1 | 0.020385 |
| GO:0015771 | trehalose transport | 18 | 1 | 0.0205 |
| GO:0045619 | regulation of lymphocyte differentiation | 17 | 1 | 0.020534 |
| GO:0042454 | ribonucleoside catabolic process | 19 | 1 | 0.021353 |
| GO:0032944 | regulation of mononuclear cell proliferation | 19 | 1 | 0.021656 |
| GO:0050670 | regulation of lymphocyte proliferation | 19 | 1 | 0.021656 |
| GO:0070663 | regulation of leukocyte proliferation | 19 | 1 | 0.021656 |
| GO:0016106 | sesquiterpenoid biosynthetic process | 19 | 1 | 0.022195 |
| GO:0015766 | disaccharide transport | 20 | 1 | 0.022942 |
| GO:0015772 | oligosaccharide transport | 20 | 1 | 0.022942 |
| GO:0009617 | response to bacterium | 200 | 2 | 0.023426 |
| GO:0000097 | sulfur amino acid biosynthetic process | 22 | 1 | 0.024828 |
| GO:0006714 | sesquiterpenoid metabolic process | 22 | 1 | 0.025504 |
| GO:0009164 | nucleoside catabolic process | 24 | 1 | 0.027164 |
| GO:0015985 | energy coupled proton transport, down electrochemical gradient | 24 | 1 | 0.02729 |
| GO:0015986 | ATP synthesis coupled proton transport | 24 | 1 | 0.02729 |
| GO:1901135 | carbohydrate derivative metabolic process | 572 | 3 | 0.02764 |
| GO:0009067 | aspartate family amino acid biosynthetic process | 25 | 1 | 0.028103 |
| GO:0051251 | positive regulation of lymphocyte activation | 24 | 1 | 0.028731 |
| GO:0016114 | terpenoid biosynthetic process | 25 | 1 | 0.028902 |
| GO:0002696 | positive regulation of leukocyte activation | 25 | 1 | 0.029829 |
| GO:0050867 | positive regulation of cell activation | 25 | 1 | 0.029829 |
| GO:0050863 | regulation of T cell activation | 25 | 1 | 0.029909 |
| GO:1903037 | regulation of leukocyte cell-cell adhesion | 25 | 1 | 0.029909 |
| GO:0006754 | ATP biosynthetic process | 29 | 1 | 0.032773 |
| GO:1902107 | positive regulation of leukocyte differentiation | 27 | 1 | 0.032809 |
| GO:0046496 | nicotinamide nucleotide metabolic process | 28 | 1 | 0.033792 |
| GO:0098542 | defense response to other organism | 241 | 2 | 0.03401 |
| GO:0009166 | nucleotide catabolic process | 30 | 1 | 0.034723 |
| GO:0019362 | pyridine nucleotide metabolic process | 29 | 1 | 0.034887 |
| GO:1901658 | glycosyl compound catabolic process | 31 | 1 | 0.035076 |
| GO:0000096 | sulfur amino acid metabolic process | 32 | 1 | 0.035823 |
| GO:1901292 | nucleoside phosphate catabolic process | 32 | 1 | 0.036904 |
| GO:0009206 | purine ribonucleoside triphosphate biosynthetic process | 34 | 1 | 0.038266 |
| GO:0006082 | organic acid metabolic process | 653 | 3 | 0.039178 |
| GO:0009145 | purine nucleoside triphosphate biosynthetic process | 35 | 1 | 0.039361 |
| GO:0009117 | nucleotide metabolic process | 268 | 2 | 0.039425 |
| GO:0044283 | small molecule biosynthetic process | 273 | 2 | 0.039693 |
| GO:0006753 | nucleoside phosphate metabolic process | 271 | 2 | 0.040191 |
| GO:0009201 | ribonucleoside triphosphate biosynthetic process | 36 | 1 | 0.040788 |
| GO:0006334 | nucleosome assembly | 31 | 1 | 0.040837 |
| GO:0051249 | regulation of lymphocyte activation | 35 | 1 | 0.041597 |
| GO:0006144 | purine nucleobase metabolic process | 36 | 1 | 0.042703 |
| GO:0009306 | protein secretion | 36 | 1 | 0.043665 |
| GO:1901137 | carbohydrate derivative biosynthetic process | 287 | 2 | 0.044164 |
| GO:0008299 | isoprenoid biosynthetic process | 39 | 1 | 0.044197 |
| GO:0072524 | pyridine-containing compound metabolic process | 38 | 1 | 0.044715 |
| GO:0072523 | purine-containing compound catabolic process | 40 | 1 | 0.046283 |
| GO:0022409 | positive regulation of cell-cell adhesion | 39 | 1 | 0.046597 |
| GO:1902105 | regulation of leukocyte differentiation | 39 | 1 | 0.047442 |
| GO:0009066 | aspartate family amino acid metabolic process | 43 | 1 | 0.047834 |
| GO:0034110 | regulation of homotypic cell-cell adhesion | 40 | 1 | 0.047843 |
| GO:0006733 | oxidoreduction coenzyme metabolic process | 42 | 1 | 0.049018 |
| GO:0009142 | nucleoside triphosphate biosynthetic process | 44 | 1 | 0.049459 |

Table S16. Molecular function GO term up-regulated in lure-mated females when compared with control-mated females

| **GO Category** | **GO Function** | **Numbers in category** | **Number differentially expressed** | **P value** |
| --- | --- | --- | --- | --- |
| GO:0016763 | transferase activity, transferring pentosyl groups | 27 | 2 | 0.000447 |
| GO:0017061 | S-methyl-5-thioadenosine phosphorylase activity | 3 | 1 | 0.00338 |
| GO:0002060 | purine nucleobase binding | 3 | 1 | 0.003383 |
| GO:0004731 | purine-nucleoside phosphorylase activity | 3 | 1 | 0.003383 |
| GO:0042301 | phosphate ion binding | 4 | 1 | 0.004507 |
| GO:0002054 | nucleobase binding | 4 | 1 | 0.004648 |
| GO:0008422 | beta-glucosidase activity | 9 | 1 | 0.010515 |
| GO:0004553 | hydrolase activity, hydrolyzing O-glycosyl compounds | 147 | 2 | 0.012497 |
| GO:0003796 | lysozyme activity | 12 | 1 | 0.013456 |
| GO:0016798 | hydrolase activity, acting on glycosyl bonds | 162 | 2 | 0.015102 |
| GO:0046933 | proton-transporting ATP synthase activity, rotational mechanism | 14 | 1 | 0.015848 |
| GO:0047886 | farnesol dehydrogenase activity | 15 | 1 | 0.016793 |
| GO:0015151 | alpha-glucoside transmembrane transporter activity | 18 | 1 | 0.0205 |
| GO:0015574 | trehalose transmembrane transporter activity | 18 | 1 | 0.0205 |
| GO:0042947 | glucoside transmembrane transporter activity | 18 | 1 | 0.0205 |
| GO:0015154 | disaccharide transmembrane transporter activity | 20 | 1 | 0.022942 |
| GO:0015157 | oligosaccharide transmembrane transporter activity | 20 | 1 | 0.022942 |
| GO:0016757 | transferase activity, transferring glycosyl groups | 202 | 2 | 0.02329 |
| GO:0004568 | chitinase activity | 29 | 1 | 0.032335 |
| GO:0030145 | manganese ion binding | 30 | 1 | 0.034791 |
| GO:0008144 | drug binding | 30 | 1 | 0.035744 |
| GO:0044769 | ATPase activity, coupled to transmembrane movement of ions, rotational mechanism | 35 | 1 | 0.039486 |
| GO:0015926 | glucosidase activity | 37 | 1 | 0.042641 |
| GO:0051119 | sugar transmembrane transporter activity | 43 | 1 | 0.048434 |
